# High Myopia as a Risk Factor for Severe Liver Disease in Individuals with Liver Dysfunction: Evidence from a Prospective Cohort

**DOI:** 10.3390/jcm14165860

**Published:** 2025-08-19

**Authors:** Linge Jian, Zhiqian Huang, Yu Du, Xiangjia Zhu

**Affiliations:** 1 Eye Institute and Department of Ophthalmology, Eye & ENT Hospital, Fudan University, Shanghai 200031, China; 18307110308@fudan.edu.cn (L.J.); 23111260009@m.fudan.edu.cn (Z.H.); 17111260004@fudan.edu.cn (Y.D.); 2 NHC Key Laboratory of Myopia, Fudan University, Shanghai 200031, China; 3 Key Laboratory of Myopia, Chinese Academy of Medical Science, Shanghai 200031, China; 4 Shanghai Key Laboratory of Visual Impairment and Restoration, Shanghai 200031, China

**Keywords:** high myopia, aspartate aminotransferase, liver fibrosis, C-reactive protein, cirrhosis

## Abstract

**Background/Objectives**: Although high myopia primarily affects the eyes, emerging evidence suggests that it is also associated with systemic inflammation and metabolic dysfunction. The liver plays a key role in metabolism and inflammation, and it may share pathological pathways with high myopia. However, no population studies have examined the relationship between high myopia and liver disease progression. This study used UK Biobank data to analyze the relationship between myopia severity and severe liver disease, as well as to determine whether inflammatory markers or metabolites mediate this link. **Methods**: A prospective cohort of 70,774 UK Biobank participants without severe liver disease at baseline was followed for 14.1 years. Myopia was categorized as emmetropia, low-to-moderate, or high based on refractive error. Cox proportional hazards models, stratified by aspartate aminotransferase (AST) level (≥40 vs. <40 U/L), were used to assess liver disease risk, and mediation analyses were used to evaluate inflammatory markers and metabolites. **Results**: Among participants with AST levels of at least 40 U/L, high myopia significantly increased liver fibrosis and cirrhosis risk (hazard ratio [HR] = 2.64, 95% confidence interval [CI] = 1.44–4.85, *p* = 0.002), exhibiting a dose-dependent trend (*p*trend = 0.004). No association existed for AST < 40 U/L. C-reactive protein (CRP) partially mediated this link; no metabolites survived correction. **Conclusions**: High myopia is independently associated with an increased risk of liver fibrosis and cirrhosis in individuals with elevated AST, partially mediated by CRP-related inflammation. Refractive assessment may stratify liver disease risk in subclinical injury, warranting anti-inflammatory intervention research.

## 1. Introduction

The global prevalence of myopia is projected to reach 50% by 2050, including 10% with high myopia [1,2]. High myopia is traditionally considered an ocular disease, but emerging evidence suggests systemic manifestations, including neuropsychiatric comorbidities [3], gut microbiota dysbiosis [4], and systemic inflammatory or metabolic dysregulation [5,6]. Recent studies have linked progressive myopia to higher circulating inflammatory markers and metabolic dysregulation [7,8]. High myopia is associated with systemic inflammation and oxidative stress [9,10,11], and the liver is a central organ in metabolic and inflammatory regulation [12,13]. These overlapping pathways suggest plausible biological links between high myopia and liver disease progression. Retinal oxidative stress associated with high myopia may interfere with mitochondrial function in distal organs through circulating diffusion of proinflammatory factors and systemic redox imbalance [11]. Mitochondria in the liver, a highly metabolic organ, are particularly sensitive to circulating oxidizing factors. Preclinical studies suggest that systemic inflammation and metabolic dysregulation exacerbate liver injury through oxidative stress and fibrosis pathways [14,15]. However, the role of myopia in liver disease remains unexplored, with no population-level evidence linking myopia to the progression of liver disease.

To investigate this interaction in humans, we used the prospective design of the UK Biobank to examine the relationship between liver and eye, stratifying participants by aspartate aminotransferase (AST) level (≥40 vs. <40 U/L). This cut-off was chosen because in a prior population-based cohort (*n* = 446,000), individuals with AST ≥ 40 IU/L experienced a 10.2-year reduction in life expectancy and a doubling of liver-related mortality compared with those with elevated ALT, establishing AST ≥ 40 IU/L as a robust marker of subclinical liver injury [16].

We hypothesize that high myopia-related systemic inflammation contributes to liver dysfunction. To address this gap, we investigated the underlying biological mechanisms contributing to this association, providing new epidemiological evidence linking ocular and hepatic pathophysiology to inform risk stratification strategies.

## 2. Materials and Methods

The UK Biobank (UKB) is a large prospective cohort study that aims to identify determinants of complex diseases in middle-aged and older Europeans. Conducted between 2006 and 2010, it involved more than 500,000 participants aged 37 to 73 who completed questionnaires, underwent physical examinations, and provided biological samples at 22 centers in England, Scotland, and Wales. The cohort links hospitalization and mortality records for continuous monitoring of morbidity and mortality. Data collection included touch-screen surveys, interviews, examinations, samples, and imaging. Detailed protocols are available on the UK Biobank website [17,18].

**Figure 1:**
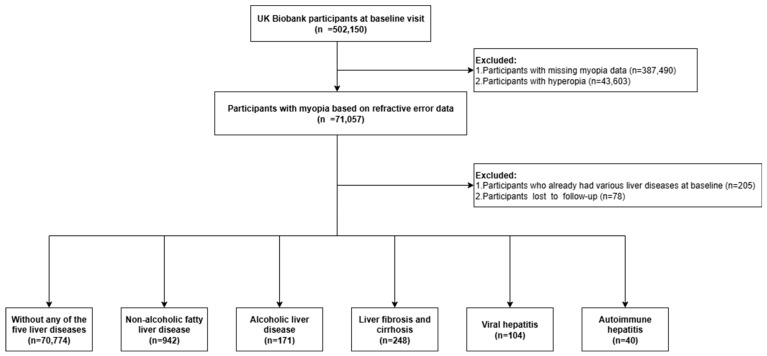
A flowchart illustrating the criteria for the study group derived from participants in the UK Biobank.

For our analysis, we excluded participants without refractive error measurements (n = 387,490). Hyperopic participants (spherical equivalent > +0.75 D, n = 43,603) were excluded because hyperopia represents a distinct axial-length pattern and metabolic profile that could dilute or reverse the association under investigation [19,20]. We also excluded individuals with severe liver disease at baseline, including hepatic fibrosis and cirrhosis, non-alcoholic fatty liver disease (NAFLD), alcoholic liver disease (ALD), viral hepatitis, and autoimmune hepatitis (AIH) (n = 205). Participants lost to follow-up during the median 14.1 years of follow-up were excluded (n = 78). This left 70,774 participants for the final analysis (Figure 1).

**Ethics**

The research ethics committee of the North West Multi-Center granted approval for the UKB study. All participants signed an electronic consent form at recruitment. This research was approved under application number 93,118.

**Assessment of exposure**

In the UKB, refractive error was assessed utilizing the RC-5000 device (Tomey Corporation, Nagoya, Japan). Myopia severity was classified with spherical equivalent (SE) because SE is the standard metric in large-scale epidemiology and correlates strongly with axial length [21]. The mean spherical equivalent refractive error (avMSE) was calculated as the average between the fellow eyes and expressed in diopters (UKB data field 20261). The spherical equivalent was determined by summing the spherical power and half the cylinder power, with values averaged between the fellow eyes. Emmetropia was defined as a spherical equivalent refractive error of −0.75 D to +0.75 D; hyperopia was defined as ≥+0.75 D [22]. To ensure the quality of the autorefraction data, the UKB protocol excluded participants who reported a history of cataract, cataract surgery, corneal transplantation, laser eye surgery, or significant ocular trauma, or if the autorefraction readings had “low reliability” or “lower reliability” error messages. Myopia diagnoses (UKB data field 20262) were categorized into three groups: emmetropia, low to moderate myopia (≤−0.75D), and high myopia (≤−6.00 D) [22]. The latter is the IMI consensus threshold that marks a steep rise in posterior-segment pathologies [21] and has been consistently applied in recent UK Biobank analyses.

**Assessment of outcome**

Liver disease outcomes were ascertained through linkage with hospitalization records using International Classification of Diseases (ICD-9 and ICD-10) codes (Supplementary Table S1), consistent with prior UK Biobank studies [23,24]. Specifically, we defined incident severe liver diseases as first hospitalization events meeting diagnostic criteria for liver fibrosis and cirrhosis, NAFLD, ALD, viral hepatitis, or AIH. For consistency and specificity, only participants with confirmed hospitalizations meeting these coding criteria were included in the primary analysis.

Mortality data for England and Wales were obtained from NHS Digital and for Scotland from the NHS Central Register. Follow-up was from the date of recruitment to the earliest of liver disease diagnosis, death, or censoring. Censoring dates were based on hospital admission data from the Hospital Episode Statistics for England (HES; up to 31 October 2022), Scottish Morbidity Record (SMR; up to 31 August 2022), and Patient Episode Database for Wales (PEDW; up to 31 May 2022), and death registry records from the National Health Service (NHS) Information Centre (England and Wales, up to 30 November 2022) and the NHS Central Register, National Records of Scotland (Scotland, up to 30 November 2022).

**Assessment of potential mediators**

Based on potential pathways identified in previous studies [3,9,10,25], we selected 6 inflammatory blood biomarkers, 5 indicators of complex inflammation, and 249 plasma metabolites as potential mediators. During the baseline recruitment phase of the UKB, blood samples were collected from consenting participants. Approximately 4 ml of blood was collected, processed to separate components, stored at −80 °C, and analyzed within 24 h using a Beckman Coulter LH750 instrument (Brea, CA, United States) [17]. Blood biomarkers underwent rigorous quality control and external validation [17]. Plasma samples from approximately one-fifth of the baseline participants were analyzed for metabolites using Nightingale Health’s NMR-based high-throughput metabolic biomarker analysis platform. According to the UKB’s online documentation [26], 249 metabolites were quantified in molar concentration units, and these data can be used directly for epidemiological analyses without pre-processing.

Inflammation-related biomarkers included leukocyte count, neutrophil count, monocyte count, lymphocyte count, platelet count, and C-reactive protein (CRP). The complex inflammation indicators included the neutrophil-to-lymphocyte ratio (NLR), platelet-to-lymphocyte ratio (PLR), monocyte-to-high-density-lipoprotein-cholesterol ratio (MHR), lymphocyte-to-monocyte ratio (LMR), and the low-grade chronic inflammation (INFLA) score. The INFLA score was calculated using CRP, white blood cell (WBC) count, platelet count, and NLR according to established methods. Each biomarker was assigned a score from −4 to +4 based on population deciles: lower deciles (1st–4th) received negative weights (−4 to −1), higher deciles (7th–10th) received positive weights (+1 to +4), and intermediate deciles (5th–6th) were assigned zero. The composite score, ranging from −16 to +16, reflects the overall inflammatory status, with positive values indicating proinflammatory dominance.

**Assessment of covariates**

A touch-screen questionnaire collected self-reported data on participants’ smoking status, frequency of alcohol consumption, sex, ethnicity, age at enrollment, and year of birth. The Townsend deprivation index (TDI) was calculated based on the participants’ postcodes. Alcohol frequency was categorized into four distinct groups: never, 1–5 drinks/month, 5–10 drinks/month, and more than 10 drinks/month. Smoking status was categorized as never smoking or ever/current smoker. Physical activity was assessed using the International Physical Activity Questionnaire (IPAQ) to determine whether participants engaged in moderate, vigorous, or walking-based activities, defined as ≥150 minutes/week of walking or moderate activity, or ≥75 min/week of vigorous activity, according to the 2017 UK Physical Activity Guidelines. Trained nurses manually measured waist circumference. Body mass index (BMI) was calculated from participants’ height and weight. Educational attainment was categorized into three levels: college and above, high school, and below high school. Blood samples were analyzed for concentrations of triglycerides, AST, alanine aminotransferase (ALT), and high-density lipoprotein (HDL) (Supplementary Table S2). A trained nurse conducted verbal interviews to obtain participants’ history of hypertension and diabetes, with comprehensive definitions (Supplementary Table S3).

**Statistical analyses**

Baseline demographic data were reported as medians (interquartile range) for continuous variables and frequencies (percentages) for categorical variables. Continuous variables were compared using the Kruskal–Wallis test, and categorical variables were compared using Pearson’s chi-squared test. Missing covariate data were imputed using multiple imputation by chained equations (MICE) with 20 imputations and 10 iterations using the “mice” package (v3.15.0) in R software.

To assess the association between myopia severity and risk of liver disease, we used Cox proportional hazards models stratified by baseline AST levels (≥40 U/L vs. <40 U/L), adjusting for covariates in Models 1–4. The 40 U/L AST threshold was used because it is supported by historical studies and has predictive ability for liver-related mortality. The proportional hazards assumption was validated using Schoenfeld residuals (*p* > 0.05). Four models were constructed: Model 1 (crude), Model 2 (adjusted for age, sex, ethnicity, Townsend deprivation index), Model 3 (further adjusted for alcohol, smoking, physical activity), and Model 4 (further adjusted for education, waist circumference, hypertension, diabetes, ALT, HDL).

Subgroup analyses were performed by age, sex, race, waist circumference, education, smoking, alcohol consumption, physical activity, and history of hypertension or diabetes. Interaction effects were assessed using likelihood ratio tests comparing Cox models with and without interaction terms. In sensitivity analyses, individuals who developed liver disease within six months of myopia diagnosis were reclassified as unexposed to account for reverse causality. Additionally, a sensitivity analysis using 35 U/L as the AST threshold was performed.

Mediation analyses were performed in two steps: (1) linear regression (high myopia → metabolite) and Cox regression (metabolite → liver disease) with Benjamini–Hochberg FDR correction at 5%, retaining metabolites that passed both corrections; (2) further FDR correction of indirect effect *p*-values from the mediation analysis. This two-step methodology ensured stringent control over type I error inflation. The proportion mediated (PM) was estimated using the “mediation” package, with non-parametric bootstrapping (5000 draws) for 95% CIs.

All analyses were conducted using R software (version 4.0.2), and a two-sided *p*-value of less than 0.05 was deemed statistically significant.

**Role of the funding source**

The sponsors of the research were not involved in the study’s design, data collection, data analysis, data interpretation, report writing, or the decision to submit the manuscript for publication.

## 3. Results

### 3.1. Baseline Characteristics

Our analysis included 70,774 participants from the UKB with complete data, consisting of 32,968 males (46.6%) and 37,806 females (53.4%), with a mean enrollment age of 56 years. At baseline, 30,022 individuals had low-to-moderate myopia, and 4572 had high myopia.

Baseline characteristics stratified by myopia status revealed that high myopia was associated with a higher proportion of females, greater socioeconomic deprivation, and elevated inflammatory markers (NLR and PLR) (Table 1).

During a median follow-up of 14.1 years, 248 cases of liver fibrosis and cirrhosis, 171 cases of ALD, 942 cases of NAFLD, 104 cases of viral hepatitis, and 40 cases of AIH were recorded.

### 3.2. Myopia Status and Risk of Incident Severe Liver Diseases

In multivariable Cox models (Supplementary Table S4), high myopia showed a moderate association with incident liver fibrosis and cirrhosis (HR = 1.85, 95% CI = 1.14–3.00, *p* = 0.012) compared to emmetropia. No significant associations were found for other liver outcomes. We stratified participants by baseline AST level (≥40 vs. <40 U/L) to assess effect modification. Baseline AST did not differ significantly by myopia severity (*p* > 0.05) (Supplementary Table S5).

**Figure 2:**
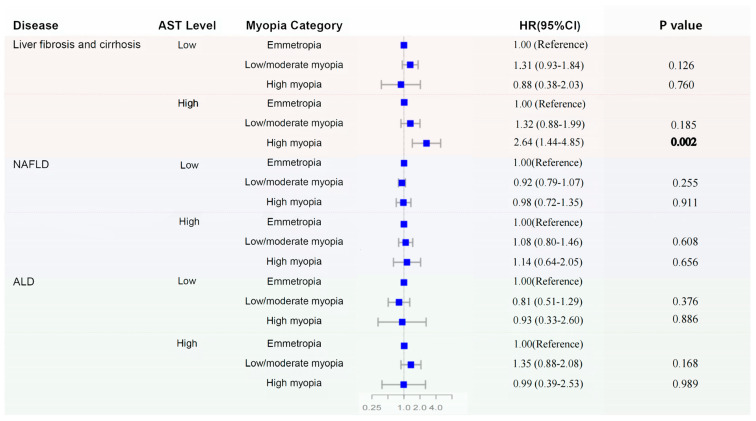
Association between myopia status and liver diseases stratified by AST level. Adjusted model includes age, sex, ethnicity, Townsend deprivation index, smoking status, alcohol consumption, physical activity, education, waist circumference, hypertension status, diabetes status, ALT, and HDL levels. HR, hazard ratio; CI, confidence interval; NAFLD, non-alcoholic fatty liver disease; ALD, alcoholic liver disease; AIH, autoimmune hepatitis; ALT, alanine aminotransferase; AST, aspartate aminotransferase; HDL, high-density lipoprotein. Bolded values indicate statistical significance (*p* < 0.05).

Stratified analyses by AST level showed a significant association only in participants with AST ≥ 40 U/L: in the fully adjusted model (Model 4), high myopia was associated with an increased risk of liver fibrosis and cirrhosis (HR = 2.64, 95% CI = 1.44–4.85, *p* = 0.002), with a clear dose-dependent trend (*p*trend = 0.004). No significant association was observed in participants with AST < 40 U/L (all *p* > 0.05) (Table 2, Figure 2). Furthermore, the risk of liver fibrosis and cirrhosis increased linearly with decreasing avMSE. On average, for each per-unit (D) increase in avMSE, the risk of liver fibrosis and cirrhosis was reduced by 10% (HR = 0.90, 95% CI = 0.85–0.97, *p* = 0.003). To further illustrate the absolute risk of liver fibrosis and cirrhosis across different myopia statuses, Kaplan–Meier curves visually demonstrating the cumulative incidence of this outcome in the study population were generated (Supplementary Figure S1).

Preliminary analyses for viral hepatitis and AIH suggested potential associations, but were underpowered due to limited event counts (Supplementary Table S6). Sensitivity analyses supported the robustness of the primary findings (Supplementary Tables S7 and S8).

### 3.3. Subgroup Analyses in High-Risk Subgroups

Subgroup analyses within the AST ≥ 40 U/L stratum revealed significant effect modification for liver fibrosis and cirrhosis by ethnicity (non-white vs. white, Pinteraction = 0.007) and by alcohol consumption (5–10 drinks per month; HR = 5.73, 95% CI 1.87–17.53, *p* = 0.002). A borderline interaction was noted for diabetes status (Pinteraction = 0.078), with the elevated risk confined to non-diabetic individuals (HR = 2.78, 95% CI 1.45–5.35, *p* = 0.002).

No significant interactions were observed for age, sex, waist circumference, education, smoking, physical activity, or hypertension status (all *p*interaction > 0.05; Supplementary Tables S9–S11).

### 3.4. Mediation Effects of Inflammatory and Metabolic Biomarkers

**Figure 3:**
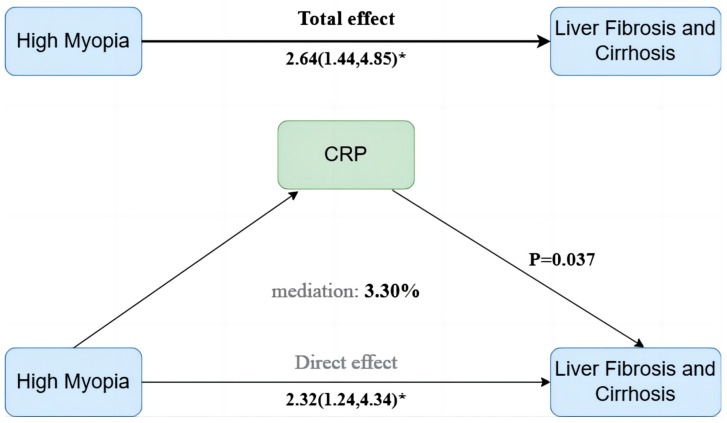
Mediation analyses of CRP in the association between high myopia and liver fibrosis and cirrhosis within the elevated AST subgroup. AST, aspartate aminotransferase; CRP, C-reactive protein. * indicates statistical significance with a *p*-value < 0.05.

We hypothesized that inflammatory and metabolic biomarkers might mediate the association between high myopia and liver pathophysiology. In the AST ≥ 40 U/L subgroup, mediation analyses assessed 6 inflammatory biomarkers, 5 composite indices, and 249 metabolites. After rigorous FDR correction, only CRP showed a small but statistically significant mediation effect, explaining 3.3% of the total association (95% CI: 0.23–7.70%, *p* = 0.037) (Figure 3). No other biomarkers or indices retained significance after adjustment (all FDR ≥ 0.05).

Plasma metabolomics did not identify any mediators that survived FDR correction. However, exploratory analyses suggested preliminary associations with changes in very-low-density lipoprotein (VLDL) and HDL subfractions (Supplementary Tables S12–S15).

## 4. Discussion

This large prospective cohort study shows a significant association between high myopia and progression to liver fibrosis and cirrhosis in individuals with baseline AST ≥ 40 U/L. The dose-dependent risk gradient with increasing myopia severity (*p*trend = 0.004) suggests a cumulative biological mechanism, probably driven by systemic inflammation and oxidative stress. This continuous relationship implies that high myopia should be used as a continuous predictor for risk assessment rather than a binary classification. Our results suggest that refractive error may be considered as an adjunctive indicator in liver disease risk stratification for individuals with elevated AST. Although CRP only mediated a small proportion of the associations, the results are biologically plausible. The partially mediated effect of CRP suggests that systemic inflammation may be involved in the co-morbid mechanism of high myopia and liver fibrosis and cirrhosis. However, the low mediator ratio suggests that other mechanisms, such as intestinal flora dysbiosis or mitochondrial dysfunction, may also be involved. This needs to be verified by subsequent studies that integrate multi-omics data.

AST levels significantly influenced the association between high myopia and liver disease, with an increased risk only in the AST ≥ 40 U/L subgroup. In this subgroup, high myopia was a significant risk factor for liver fibrosis and cirrhosis, with a clear dose-dependent relationship. The differential effects of AST in different subgroups may stratify liver susceptibility. This threshold is clinically pragmatic and has previously predicted liver-related mortality in population studies [16]. Preclinical studies have shown that elevated AST indicates mitochondrial fragility, which in turn leads to ATP depletion and oxidative stress, impairing the ability of hepatocytes to repair damage [27,28]. Chronic inflammation associated with high myopia may exacerbate mitochondrial dysfunction and overwhelm hepatic compensatory mechanisms [3,7,27,29]. Beyond systemic spill-over of inflammatory cytokines, recent pre-clinical evidence suggests a mechanistic pathway linking axial elongation to hepatic fibrosis. Biomechanical stretching of the posterior sclera up-regulates HIF-1α, triggering the release of extracellular-vesicle-packaged microRNA-21 into the circulation [30,31,32]; these vesicles are subsequently internalized by hepatic stellate cells, augmenting TGF-β/Smad-mediated collagen deposition [33,34]. Although this pathway remains to be validated in vivo, it provides a potential molecular bridge between ocular biomechanical strain and hepatic fibrogenesis. Notably, the non-significant variation in baseline AST levels across myopia status (*p* = 0.34) supports the role of AST as an effect modifier rather than a mediator. This effect modification framework opens the way to investigate potential mediators within this pathway.

The modest mediation effect of CRP suggests that systemic inflammation has a limited but discernible effect on the relationship between myopia and liver fibrosis and cirrhosis. The high-sensitivity CRP/high-density lipoprotein cholesterol (hs-CRP/HDL-C) index was shown to correlate positively with both metabolic dysfunction-associated steatotic liver disease (MASLD) and advanced liver fibrosis [35]. Even though CRP showed statistically significant mediation, it accounted for only 3.3% (95% CI 0.23–7.70 %) of the total association, underscoring that systemic inflammation is, at most, a minor contributor. Large proportions of the effect remain unexplained, and several alternative pathways should be considered: (i) metabolic-syndrome-related factors that associate with both myopia and liver fibrosis; (ii) pleiotropic genetic variants or shared heritability influencing ocular and hepatic extracellular-matrix biology; and (iii) residual confounding by socioeconomic status or unmeasured comorbidities [36,37,38]. Future multi-omics and Mendelian-randomization studies are needed to disentangle these possibilities. Exploratory metabolomic analyses identified changes in VLDL/HDL subfractions, but these associations did not survive FDR correction, highlighting the need for mechanistic validation in co-culture systems integrating retinal and hepatic tissues [39,40,41].

The different associations between liver disease subtypes may reflect different underlying mechanisms. The lack of a significant association between high myopia and NAFLD after metabolic adjustment suggests a different etiological pathway than insulin resistance [42,43,44]. The direct hepatotoxic effects of alcohol may mask the contribution of systemic inflammation in ALD [45,46,47]. Although high myopia is associated with an increased risk of viral and autoimmune hepatitis, the small number of cases and wide confidence intervals make definitive conclusions difficult.

The strengths of this study include its population-based design, prospective approach with registry linkage, and extensive adjustment for confounding variables. It represents the first epidemiological demonstration that high myopia is independently associated with accelerated liver fibrosis and cirrhosis progression in individuals with elevated AST. By using AST-stratified Cox models in conjunction with CRP mediation analyses, this study advances the mechanistic understanding of the relationship between myopia and liver disease and identifies systemic inflammation as a potential biological pathway. These contributions—quantifying the epidemiological risk associated with high myopia and elucidating its pathological mechanisms—fill critical gaps in our knowledge of its multisystemic effects and support the inclusion of refractive assessment in liver disease monitoring protocols.

Major limitations include possible residual confounding from undiagnosed liver disease or unmeasured inflammatory conditions, and the absence of liver biopsy data, which may lead to misclassification of disease severity. The predominance of participants of European ancestry in the UK Biobank limits generalizability to other populations. Finally, exploratory analyses of viral and autoimmune hepatitis were underpowered and should be interpreted with caution.

Future research should focus on three areas. First, the role of CRP-mediated inflammatory pathways in the association of myopia with liver fibrosis and cirrhosis requires mechanistic validation using in vitro and in vivo models. Second, predictive models integrating refractive error, AST pathways, and inflammatory biomarkers such as CRP for early risk stratification in patients with liver impairment should be developed. Third, there is a need to conduct clinical trials targeting systemic inflammation to assess the therapeutic potential in reducing the progression of myopia-associated liver disease. Future preclinical models should focus on CRP-related systemic inflammation to explore its causative role.

## 5. Conclusions

In conclusion, high myopia is independently associated with an increased risk of liver fibrosis and cirrhosis in individuals with elevated AST (≥40 U/L), with a small portion of this association being mediated by CRP. These findings provide preliminary epidemiological evidence for a link between high myopia and liver disease progression, warranting further mechanistic and clinical studies to validate the observed associations.

## Figures and Tables

**Table 1:** Baseline characteristics by myopia status.

Variables	Total (n = 70,774)	Myopia Status			Statistic	*p*
		Emmetropia (n = 36,180)	Low/Moderate Myopia (n = 30,022)	High Myopia (n = 4572)		
Age, M (Q_1_, Q_3_)	56.00 (48.00, 62.00)	54.00 (47.00, 62.00)	57.00 (49.00, 63.00)	56.00 (49.00, 62.00)	χ^2^ = 536.03 #	<0.001
Sex, n (%)					χ^2^ = 50.94	<0.001
Male	32,968 (46.58)	1917 (41.93)	13,896 (46.29)	17,155 (47.42)		
Female	37,806 (53.42)	2655 (58.07)	16,126 (53.71)	19,025 (52.58)		
Ethnicity, n (%)					χ^2^ = 82.88	<0.001
Non-white	13,944 (19.70)	879 (19.23)	5463 (18.20)	7602 (21.01)		
White	56,830 (80.30)	3693 (80.77)	24,559 (81.80)	28,578 (78.99)		
Townsend deprivation index, M (Q_1_, Q_3_)	−1.52 (−3.25, 1.11)	−1.45 (−3.23, 1.30)	−1.62 (−3.31, 0.95)	−1.45 (−3.20, 0.98)	χ^2^ = 63.84 #	<0.001
Education, n (%)					χ^2^ = 1418.00	<0.001
Below high school	27,715 (39.16)	2446 (53.50)	13,146 (43.79)	12,123 (33.51)		
High school	8767 (12.39)	610 (13.34)	3916 (13.04)	4241 (11.72)		
College or above	34,292 (48.45)	1516 (33.16)	12,960 (43.17)	19,816 (54.77)		
Physical activity, n (%)					χ^2^ = 166.82	<0.001
Low	12,174 (17.20)	802 (17.54)	5342 (17.79)	6030 (16.67)		
Moderate	29,440 (41.60)	2064 (45.14)	12,958 (43.16)	14,418 (39.85)		
High	29,160 (41.20)	1706 (37.31)	11,722 (39.04)	15,732 (43.48)		
BMI, M (Q_1_, Q_3_)	26.60 (23.99, 29.77)	26.74 (24.11, 29.91)	26.53 (23.92, 29.66)	26.03 (23.50, 29.19)	χ^2^ = 104.29 #	<0.001
BMI group, n (%)					χ^2^ = 96.62	<0.001
Low	405 (0.57)	47 (1.03)	168 (0.56)	190 (0.53)		
Normal	23,943 (33.83)	1727 (37.77)	10,402 (34.65)	11,814 (32.65)		
Overweight	29,632 (41.87)	1857 (40.62)	12,486 (41.59)	15,289 (42.26)		
Obesity	16,794 (23.73)	941 (20.58)	6966 (23.20)	8887 (24.56)		
Waist, M (Q_1_, Q_3_)	90.00 (80.00, 99.00)	90.00 (81.00, 99.00)	90.00 (80.00, 99.00)	88.00 (79.00, 97.00)	χ^2^ = 75.24 #	<0.001
Alcohol Group, n (%)					χ^2^ = 105.30	<0.001
1–5 drinks/month	17,066 (24.11)	1141 (24.96)	7163 (23.86)	8762 (24.22)		
10+ drinks/month	29,900 (42.25)	1994 (43.61)	13,169 (43.86)	14,737 (40.73)		
5–10 drinks/month	17,575 (24.83)	1084 (23.71)	7274 (24.23)	9217 (25.48)		
Non-drinker	6233 (8.81)	353 (7.72)	2416 (8.05)	3464 (9.57)		
Smoke Group, n (%)					χ^2^ = 223.80	<0.001
No	41,177 (58.18)	3019 (66.03)	17,936 (59.74)	20,222 (55.89)		
Yes	29,597 (41.82)	1553 (33.97)	12,086 (40.26)	15,958 (44.11)		
Hypertension Status, n (%)					χ^2^ = 29.39	<0.001
No	53,305 (75.32)	3510 (76.77)	22,312 (74.32)	27,483 (75.96)		
Yes	17,469 (24.68)	1062 (23.23)	7710 (25.68)	8697 (24.04)		
Diabetes status, n (%)					χ^2^ = 19.83	<0.001
No	66,874 (94.49)	4376 (95.71)	28,272 (94.17)	34,226 (94.60)		
Yes	3900 (5.51)	196 (4.29)	1750 (5.83)	1954 (5.40)		
HDL, M (Q_1_, Q_3_)	1.30 (1.10, 1.54)	1.29 (1.09, 1.54)	1.30 (1.10, 1.55)	1.32 (1.12, 1.57)	χ^2^ = 24.91 #	<0.001
AST, M (Q_1_, Q_3_)	24.50 (21.00, 29.00)	24.50 (21.00, 29.10)	24.50 (21.10, 29.00)	24.20 (20.90, 28.50)	χ^2^ = 9.79 #	0.007
ALT, M (Q_1_, Q_3_)	20.10 (15.27, 27.39)	20.16 (15.24, 27.58)	20.10 (15.35, 27.33)	19.52 (14.98, 26.32)	χ^2^ = 19.02 #	<0.001
Lymphocyte, M (Q_1_, Q_3_)	1.93 (1.56, 2.36)	1.94 (1.58, 2.38)	1.91 (1.55, 2.34)	1.89 (1.53, 2.31)	χ^2^ = 68.02 #	<0.001
Neutrophil, M (Q_1_, Q_3_)	4.09 (3.30, 5.02)	4.10 (3.29, 5.03)	4.08 (3.31, 5.01)	4.07 (3.31, 5.03)	χ^2^ = 0.90 #	0.637
Platelet, M (Q_1_, Q_3_)	236.30 (203.10, 272.60)	236.80 (203.30, 273.20)	235.90 (202.90, 271.90)	235.25 (203.40, 272.70)	χ^2^ = 4.58 #	0.101
CRP, M (Q_1_, Q_3_)	1.24 (0.61, 2.60)	1.26 (0.62, 2.62)	1.24 (0.61, 2.60)	1.20 (0.58, 2.48)	χ^2^ = 12.25 #	0.002
Monocyte, M (Q_1_, Q_3_)	0.46 (0.36, 0.57)	0.45 (0.36, 0.57)	0.46 (0.36, 0.57)	0.45 (0.36, 0.55)	χ^2^ = 15.30 #	<0.001
Leukocyte, M (Q_1_, Q_3_)	6.77 (5.73, 7.98)	6.79 (5.74, 8.02)	6.75 (5.73, 7.95)	6.74 (5.71, 7.92)	χ^2^ = 13.25 #	0.001
INFLA, M (Q_1_, Q_3_)	−1.00 (−5.00, 4.00)	−1.00 (−5.00, 4.00)	−1.00 (−5.00, 4.00)	−1.00 (−5.00, 4.00)	χ^2^ = 2.39 #	0.303
MHR, M (Q_1_, Q_3_)	0.35 (0.26, 0.47)	0.35 (0.26, 0.47)	0.35 (0.26, 0.47)	0.34 (0.25, 0.45)	χ^2^ = 23.29 #	<0.001
NLR, M (Q_1_, Q_3_)	2.11 (1.63, 2.73)	2.10 (1.62, 2.72)	2.12 (1.65, 2.75)	2.14 (1.67, 2.79)	χ^2^ = 33.88 #	<0.001
PLR, M (Q_1_, Q_3_)	122.10 (97.37, 153.67)	121.19 (96.65, 152.61)	122.94 (98.06, 154.44)	123.88 (99.30, 157.20)	χ^2^ = 43.01 #	<0.001
LMR, M (Q_1_, Q_3_)	4.25 (3.31, 5.45)	4.30 (3.35, 5.51)	4.20 (3.27, 5.39)	4.23 (3.29, 5.47)	χ^2^ = 56.96 #	<0.001

Abbreviations: BMI, body mass index; HDL, high-density lipoprotein; AST, aspartate aminotransferase; ALT, alanine aminotransferase; CRP, C-reactive protein; INFLA, low-grade chronic inflammation score; MHR, monocyte-to-high-density-lipoprotein-cholesterol ratio; NLR, neutrophil-to-lymphocyte ratio; PLR, platelet-to-lymphocyte ratio; LMR, lymphocyte-to-monocyte ratio. #, Kruskal–Wallis test; χ^2^, chi-squared test; M, median; Q_1_, 1st quartile; Q_3_, 3rd quartile.

**Table 2:** Univariate and multivariate analyses by AST-stratified Cox regression model.

Liver Diseases		AST Level	Myopia Status						avMSE	
			Emmetropia	Low/Moderate Myopia		High Myopia		*p* for Trend		
			HR (95%CI)	HR (95%CI)	*p*	HR (95%CI)	*p*		HR (95%CI)	*p*
Liver fibrosis and cirrhosis	Model 1 ^a^	<40	1 (Reference)	1.24 (0.88~1.74)	0.213	0.72 (0.31~1.65)	0.483	0.291	1.01 (0.94~1.08)	0.936
		≥40	1 (Reference)	1.28 (0.85~1.92)	0.233	2.29 (1.26~4.15)	0.006	0.014	0.92 (0.86~0.98)	0.013
	Model 2 ^b^	<40	1 (Reference)	1.25 (0.89~1.76)	0.195	0.74 (0.32~1.71)	0.483	0.325	1.00 (0.94~1.08)	0.936
		≥40	1 (Reference)	1.29 (0.86~1.93)	0.219	2.19 (1.21~3.98)	0.010	0.022	0.93 (0.87~0.99)	0.021
	Model 3 ^c^	<40	1 (Reference)	1.30 (0.93~1.83)	0.130	0.80 (0.35~1.86)	0.607	0.401	0.99 (0.93~1.06)	0.848
		≥40	1 (Reference)	1.21 (0.81~1.82)	0.353	2.20 (1.21~4.01)	0.010	0.016	0.92 (0.87~0.99)	0.019
	Model 4 ^d^	<40	1 (Reference)	1.31 (0.93~1.84)	0.126	0.88 (0.38~2.03)	0.760	0.527	0.98 (0.92~1.06)	0.669
		≥40	1 (Reference)	1.32 (0.88~1.99)	0.185	2.64 (1.44~4.85)	0.002	0.004	0.90 (0.85~0.97)	0.003
Non-alcoholic fatty liver disease	Model 1 ^a^	<40	1 (Reference)	0.86 (0.74~1.00)	0.055	0.82 (0.60~1.12)	0.215	0.423	1.04 (1.01~1.07)	0.024
		≥40	1 (Reference)	1.09 (0.81~1.47)	0.572	1.03 (0.58~1.84)	0.911	0.973	0.99 (0.93~1.05)	0.704
	Model 2 ^b^	<40	1 (Reference)	0.88 (0.76~1.02)	0.085	0.83 (0.61~1.14)	0.257	0.461	1.04 (1.00~1.07)	0.034
		≥40	1 (Reference)	1.10 (0.81~1.48)	0.541	1.00 (0.56~1.79)	0.997	0.874	0.99 (0.94~1.05)	0.802
	Model 3 ^c^	<40	1 (Reference)	0.90 (0.77~1.05)	0.168	0.88 (0.64~1.21)	0.433	0.641	1.03 (1.00~1.07)	0.091
		≥40	1 (Reference)	1.01 (0.75~1.36)	0.963	0.98 (0.55~1.76)	0.954	0.943	1.00 (0.94~1.06)	0.909
	Model 4 ^d^	<40	1 (Reference)	0.92 (0.79~1.07)	0.255	0.98 (0.72~1.35)	0.911	0.871	1.02 (0.98~1.05)	0.338
		≥40	1 (Reference)	1.08 (0.80~1.46)	0.608	1.14 (0.64~2.05)	0.656	0.746	0.98 (0.92~1.04)	0.459
Alcoholic liver disease	Model 1 ^a^	<40	1 (Reference)	0.74 (0.47~1.18)	0.206	0.67 (0.24~1.86)	0.444	0.628	1.08 (0.97~1.21)	0.153
		≥40	1 (Reference)	1.21 (0.79~1.85)	0.370	0.86 (0.34~2.17)	0.749	0.59	1.02 (0.94~1.12)	0.598
	Model 2 ^b^	<40	1 (Reference)	0.75 (0.47~1.20)	0.232	0.72 (0.26~2.01)	0.536	0.725	1.07 (0.96~1.20)	0.209
		≥40	1 (Reference)	1.25 (0.82~1.91)	0.304	0.86 (0.34~2.18)	0.758	0.577	1.02 (0.93~1.12)	0.630
	Model 3 ^c^	<40	1 (Reference)	0.76 (0.48~1.21)	0.251	0.78 (0.28~2.16)	0.627	0.819	1.06 (0.95~1.19)	0.263
		≥40	1 (Reference)	1.25 (0.82~1.92)	0.302	0.84 (0.33~2.11)	0.704	0.527	1.02 (0.93~1.12)	0.626
	Model 4 ^d^	<40	1 (Reference)	0.81 (0.51~1.29)	0.376	0.93 (0.33~2.60)	0.886	0.953	1.04 (0.93~1.16)	0.478
		≥40	1 (Reference)	1.35 (0.88~2.08)	0.168	0.99 (0.39~2.53)	0.989	0.733	1.00 (0.92~1.10)	0.933

^a^ Model 1 unadjusted (crude). ^b^ Model 2 adjusted for age at baseline, sex, ethnicity, and Townsend deprivation index. ^c^ Model 3 adjusted for Model 2 plus smoking status, alcohol consumption, and physical activity. ^d^ Model 4 adjusted for Model 3 plus education, waist circumference, hypertension status, diabetes status, ALT, and HDL. Abbreviations: HR, hazard ratio; CI, confidence interval; avMSE, mean spherical equivalent refractive error; AST, aspartate aminotransferase; ALT, alanine aminotransferase; HDL, high-density lipoprotein.

## Data Availability

All data relevant to the study were acquired from the UK Biobank Resource under application number 93118. Data can be accessed through applications on the UK Biobank website (https://www.ukbiobank.ac.uk/, accessed on 17 August 2024).

## Supplementary Materials

The following supporting information can be downloaded at https://www.mdpi.com/article/10.3390/jcm14165860/s1: Table S1. ICD codes for incident severe liver diseases in the UK Biobank; Table S2. Field codes for covariates and biomarkers; Table S3. Field codes for illness history of interest in the UK Biobank; Table S4. Association between myopia status and incident liver diseases; Table S5. Multivariable-adjusted AST values stratified by myopia status; Table S6. AST-stratified Cox regression analysis of viral and autoimmune hepatitis; Table S7. Sensitivity analyses of myopia–liver disease associations after excluding early-onset cases (6-month exclusion period); Table S8. Association Between Myopia Status and Liver Disease Incidence Using AST ≥ 35 U/L as the Threshold; Table S9. Subgroup analyses of the associations between myopia status and the risk of liver fibrosis and cirrhosis in the UKB cohort; Table S10. Subgroup analyses of the associations between myopia status and the risk of viral hepatitis in the UKB cohort; Table S11. Subgroup analyses of the associations between myopia status and the risk of autoimmune hepatitis in the UKB cohort; Table S12. Selection of blood biomarkers as potential mediators between myopia status and incident liver diseases; Table S13. Selection of metabolites as potential mediators between high myopia and incident liver fibrosis and cirrhosis; Table S14. Selection of metabolites as potential mediators between high myopia and incident viral hepatitis; Table S15. Selection of metabolites as potential mediators between high myopia and incident autoimmune hepatitis. Figure S1. Kaplan–Meier curves for cumulative incidence of liver fibrosis and cirrhosis according to myopia status.

## Abbreviations

The following abbreviations are used in this manuscript:

UKB	UK Biobank
AST	Aspartate aminotransferase
NAFLD	Non-alcoholic fatty liver disease
ALD	Alcoholic liver disease
AIH	Autoimmune hepatitis
avMSE	Mean spherical equivalent refractive error
CRP	C-reactive protein
NLR	Neutrophil-to-lymphocyte ratio
PLR	Platelet-to-lymphocyte ratio
MHR	Monocyte-to-high-density-lipoprotein-cholesterol ratio
LMR	Lymphocyte-to-monocyte ratio
WBC	White blood cell
INFLA	Low-grade chronic inflammation
TDI	Townsend deprivation index
IPAQ	International Physical Activity Questionnaire
BMI	Body mass index
ALT	Alanine aminotransferase
HDL	High-density lipoprotein
VLDL	Very-low-density lipoprotein
