# Supplementary material for: High Myopia as a Risk Factor for Severe Liver Disease in Individuals with Liver Dysfunction: Evidence from a Prospective Cohort
Source: J Clin Med. 2025 Aug 19;14(16):5860. doi: 10.3390/jcm14165860 (PMC12387578; doi:10.3390/jcm14165860)
Supplement: Supplementary file 1 [file jcm-14-05860-s001.zip › jcm-3790887-supplementary.pdf]

High Myopia Exacerbates Severe Liver Disease Risk in Individuals with Liver Dysfunction: Evidence from a Prospective Cohort

Table of contents

Table S1. ICD codes for incident severe liver diseases in the UK Biobank.

Table S2. Field codes for covariates and biomarkers.

Table S3. Field codes for illness history of interest in the UK Biobank.

Table S4. Association Between Myopia Status and Incident Liver Diseases.

Table S5. Multivariable-adjusted AST values stratified by myopia status.

Table S6. AST-stratified Cox regression analysis of viral and autoimmune hepatitis.

Table S7. Sensitivity Analyses of Myopia-Liver Disease Associations After Excluding Early-Onset Cases (6-Month Exclusion Period).

Table S8. Association Between Myopia Status and Liver Disease Incidence Using AST ≥35 U/L as the Threshold

Table S9. Subgroup analyses of the associations between myopia status and the risk of liver fibrosis and cirrhosis in the UKB cohort.

Table S10. Subgroup analyses of the associations between myopia status and the risk of viral hepatitis in the UKB cohort.

Table S11. Subgroup analyses of the associations between myopia status and the risk of autoimmune hepatitis in the UKB cohort.

Table S12. Selection of blood biomarkers as potential mediators between myopia status and incident liver diseases.

Table S13. Selection of metabolites as potential mediators between high myopia and incident liver fibrosis and cirrhosis.

Table S14. Selection of metabolites as potential mediators between high myopia and incident viral hepatitis.

Table S15. Selection of metabolites as potential mediators between high myopia and incident autoimmune hepatitis.

Figure S1. Kaplan–Meier curves for cumulative incidence of liver fibrosis and cirrhosis according to myopia status

This supplementary material has been provided by the authors to give readers additional information about their work.

Table S1. ICD codes for incident severe liver diseases in the UK Biobank.

Liver diseases	Code type	Codes
Non-alcoholic fatty liver disease	ICD-9	/
	ICD-10	K760, K758
Alcoholic liver disease	ICD-9	5710, 5711, 5712, 5713
	ICD-10	K700, K701, K702, K703, K704, K709
Viral hepatitis	ICD-9	0701, 0703, 0705, 0709, 5731
	ICD-10	B159, B160, B169, B170, B171, B172, B178, B179, B180, B181, B182, B188, B189, B199
Liver fibrosis and cirrhosis	ICD-9	5715, 5716
	ICD-10	K740, K741, K742, K743, K744, K745, K746
Autoimmune hepatitis	ICD-9	5714, 5733
	ICD-10	K732, K738, K754

Table S2. Field codes for covariates and biomarkers

Field	Code
Sex	31
Age	21022
Education	6138
Physical activity	22032
Townsend deprivation index	22189
Ethnicity	21000
Alcohol intake frequency	1558
Smoke	20116
Body mass index	21001
Waist circumference	48
Aspartate aminotransferase	30650
Alanine aminotransferase	30620
High-density lipoprotein	23406
Lymphocyte	30120
Neutrophil	30140
Platelet	30080
C-reactive protein	30710
Monocyte	30130
Leukocyte	30000

Table S3. Field codes for illness history of interest in the UK Biobank.

Illness	Components	Field codes
Hypertension	Hypertension	1065
	Essential hypertension	1072
Diabetes	Diabetes	1220
	Type 1 diabetes	1222
	Type 2 diabetes	1223
	Diabetic eye disease	1276
	Diabetic neuropathy/ulcers	1468
	Diabetic nephropathy	1607

Table S4. Association Between Myopia Status and Incident Liver Diseases

Liver diseases	Myopia Status	HR	95%CI	P
Liver fibrosis and cirrhosis	Emmetropia	1.00	Reference	NA
	Low/moderate myopia	1.40	1.07 ~ 1.83	0.015
	High myopia	1.85	1.14 ~ 3.00	0.012
Non-alcoholic fatty liver disease	Emmetropia	1.00	Reference	NA
	Low/moderate myopia	0.96	0.84 ~ 1.09	0.515
	High myopia	1.03	0.78 ~ 1.36	0.821
Alcoholic liver disease	Emmetropia	1.00	Reference	NA
	Low/moderate myopia	1.10	0.80 ~ 1.51	0.566
	High myopia	1.00	0.50 ~ 1.99	0.991
Viral hepatitis	Emmetropia	1.00	Reference	NA
	Low/moderate myopia	0.91	0.61 ~ 1.37	0.662
	High myopia	1.05	0.48 ~ 2.32	0.901
Autoimmune hepatitis	Emmetropia	1.00	Reference	NA
	Low/moderate myopia	1.60	0.86 ~ 2.97	0.137
	High myopia	2.00	0.71 ~ 5.61	0.190

Model adjusted for age, sex, ethnicity, Townsend deprivation index, smoking status, alcohol consumption, physical activity, education, waist circumference, hypertension status, diabetes status, ALT, AST and HDL.

HR Hazards ratio, CI Confidence interval, NA Not applicable, ALT Alanine aminotransferase, AST Aspartate aminotransferase, HDL High-density lipoprotein.

Table S5. Multivariable-adjusted AST values stratified by myopia status

Myopia Status	n	Crude AST (Mean±SD)	Adjusted AST[a] (95% CI)	P-value[b]
Emmetropia	36180	26.4±10.6	26.8 (26.5 ~ 27.0)	Ref
Low/Moderate myopia	30022	26.2±9.8	26.6 (26.4 ~ 26.9)	0.155
High myopia	4572	26.1±9.8	26.7 (26.3 ~ 27.0)	0.679

[a]Adjusted for age, sex, ethnicity, Townsend deprivation index, smoking status, alcohol consumption, physical activity, education, waist circumference, diabetes status, and hypertension status.

[b]P-value for comparison with emmetropia using ANCOVA.

SD Standard deviation, CI Confidence interval, AST Aspartate aminotransferase.

Table S6. AST-stratified Cox regression analysis of viral and autoimmune hepatitis

Liver diseases		AST level	Myopia Status						avMSE	
			Emmetropia	Low/moderate myopia		High myopia		p for trend		
			HR (95%CI)	HR (95%CI)	P	HR (95%CI)	P		HR (95%CI)	P
Viral hepatitis	Model 1[a]	<40	1（Reference)	0.80(0.51 ~ 1.25)	0.332	0.66(0.24 ~ 1.82)	0.421	0.548	1.07(0.96 ~ 1.18)	0.222
		≥40	1（Reference)	1.49(0.58 ~ 3.86)	0.411	2.92(0.77 ~ 11.01)	0.113	0.164	0.91(0.79 ~ 1.04)	0.170
	Model 2[b]	<40	1（Reference)	0.84(0.54 ~ 1.32)	0.453	0.69(0.25 ~ 1.92)	0.478	0.580	1.06(0.95 ~ 1.17)	0.287
		≥40	1（Reference)	1.59(0.61 ~ 4.15)	0.339	3.16(0.83 ~ 11.99)	0.091	0.145	0.90(0.78 ~ 1.04)	0.143
	Model 3[c]	<40	1（Reference)	0.84(0.54 ~ 1.32)	0.452	0.71(0.26 ~ 1.97)	0.508	0.614	1.05(0.95 ~ 1.17)	0.304
		≥40	1（Reference)	1.66(0.64 ~ 4.35)	0.300	3.66(0.96 ~ 13.94)	0.057	0.098	0.89(0.77 ~ 1.02)	0.087
	Model 4[d]	<40	1（Reference)	0.80(0.51 ~ 1.26)	0.341	0.66(0.24 ~ 1.85)	0.433	0.559	1.07(0.96 ~ 1.18)	0.222
		≥40	1（Reference)	1.60(0.59 ~ 4.30)	0.355	4.03(1.02 ~ 15.92)	0.046	0.071	0.88(0.76 ~ 1.02)	0.096
Autoimmune hepatitis	Model 1[a]	<40	1（Reference)	1.11(0.51 ~ 2.43)	0.797	0.61(0.08 ~ 4.64)	0.631	0.590	0.97(0.85 ~ 1.13)	0.696
		≥40	1（Reference)	3.54(0.94 ~ 13.33)	0.062	7.79(1.57 ~ 38.58)	0.012	0.034	0.88(0.75 ~ 1.03)	0.107
	Model 2[b]	<40	1（Reference)	1.07(0.49 ~ 2.35)	0.862	0.59(0.08 ~ 4.51)	0.611	0.582	0.98(0.84 ~ 1.13)	0.746
		≥40	1（Reference)	3.48(0.92 ~ 13.21)	0.066	6.77(1.36 ~ 33.61)	0.019	0.055	0.89(0.76 ~ 1.05)	0.166
	Model 3[c]	<40	1（Reference)	1.04(0.47 ~ 2.28)	0.924	0.56(0.07 ~ 4.27)	0.574	0.555	0.98(0.85 ~ 1.14)	0.818
		≥40	1（Reference)	3.42(0.90 ~ 13.05)	0.072	6.36(1.26 ~ 32.16)	0.025	0.070	0.90(0.76 ~ 1.06)	0.197
	Model 4[d]	<40	1（Reference)	1.06(0.48 ~ 2.34)	0.886	0.58(0.08 ~ 4.47)	0.602	0.575	0.98(0.84 ~ 1.13)	0.758
		≥40	1（Reference)	3.06(0.79 ~ 11.88)	0.107	6.08(1.17 ~ 31.67)	0.032	0.074	0.89(0.75 ~ 1.07)	0.221

[a]Model 1 unadjusted(crude).

[b]Model 2 adjusted for age at baseline, sex, ethnicity, and Townsend deprivation index.

[c]Model 3 adjusted for model 2 plus smoking status, alcohol consumption, and physical activity.

[d]Model 4 adjusted for model 3 plus education, waist circumference, hypertension status, diabetes status, ALT, and HDL.

Abbreviations: HR Hazards ratio, CI Confidence interval, avMSE Mean spherical equivalent refractive error, AST Aspartate aminotransferase, ALT Alanine aminotransferase, HDL High-density lipoprotein.

Table S7. Sensitivity Analyses of Myopia-Liver Disease Associations After Excluding Early-Onset Cases (6-Month Exclusion Period)

Liver diseases		AST level	Myopia Status						avMSE	
			Emmetropia	Low/moderate myopia		High myopia		p for trend		
			HR (95%CI)	HR (95%CI)	P	HR (95%CI)	P		HR (95%CI)	P
Liver fibrosis and cirrhosis	Model 1[a]	<40	1（Reference)	1.22(0.87 ~ 1.72)	0.248	0.72(0.31 ~ 1.65)	0.435	0.291	1.01(0.94 ~ 1.08)	0.869
		≥40	1（Reference)	1.25(0.83 ~ 1.88)	0.279	2.13(1.15 ~ 3.92)	0.016	0.014	0.93(0.87 ~ 0.99)	0.026
	Model 2[b]	<40	1（Reference)	1.23(0.88 ~ 1.73)	0.227	0.74(0.32 ~ 1.71)	0.482	0.325	1.00(0.93 ~ 1.07)	0.953
		≥40	1（Reference)	1.26(0.84 ~ 1.89)	0.268	2.04(1.10 ~ 3.76）	0.023	0.022	0.93(0.87 ~ 1.00)	0.039
	Model 3[c]	<40	1（Reference)	1.28(0.91 ~ 1.80)	1.280	0.80(0.35 ~ 1.86)	0.607	0.401	0.99(0.93 ~ 1.06)	0.828
		≥40	1（Reference)	1.18(0.78 ~ 1.78)	0.427	2.04(1.10 ~ 3.78)	0.023	0.016	0.93(0.87 ~ 1.00)	0.037
	Model 4[d]	<40	1（Reference)	1.29(0.92 ~ 1.82)	0.145	0.88(0.38 ~ 2.04)	0.767	0.527	0.98(0.92 ~ 1.05)	0.637
		≥40	1（Reference)	1.28(0.85 ~ 1.93)	0.244	2.43(1.30 ~ 4.54)	0.005	0.004	0.98(0.92 ~ 1.05)	0.009
Non-alcoholic fatty liver disease	Model 1[a]	<40	1（Reference)	0.86(0.74 ~ 1.00)	0.057	0.83(0.60 ~ 1.13)	0.232	0.423	1.04(1.01 ~ 1.07)	0.023
		≥40	1（Reference)	1.05(0.78 ~ 1.42)	0.759	1.03(0.58 ~ 1.84)	0.911	0.973	0.99(0.93 ~ 1.05)	0.723
	Model 2[b]	<40	1（Reference)	0.88(0.76 ~ 1.02)	0.086	0.84(0.61 ~ 1.15)	0.275	0.461	1.04(1.00 ~ 1.07)	0.033
		≥40	1（Reference)	1.06(0.78 ~ 1.43)	0.725	1.00(0.56 ~ 1.79)	1.000	0.874	0.99(0.94 ~ 1.05)	0.823
	Model 3[c]	<40	1（Reference)	0.90(0.77 ~ 1.05)	0.171	0.89(0.65 ~ 1.22)	0.459	0.641	1.03(1.00 ~ 1.06)	0.088
		≥40	1（Reference)	0.97(0.72 ~ 1.31)	0.836	0.98(0.55 ~ 1.76)	0.951	0.943	1.00(0.94 ~ 1.06)	0.933
	Model 4[d]	<40	1（Reference)	0.92(0.79 ~ 1.07)	0.258	0.99(0.72 ~ 1.36)	0.944	0.871	1.02(0.98 ~ 1.05)	0.329
		≥40	1（Reference)	1.04(0.77 ~ 1.41)	0.794	1.14(0.64 ~ 2.06)	0.652	0.746	0.98(0.92 ~ 1.04)	0.475
Alcoholic liver disease	Model 1[a]	<40	1（Reference)	0.72(0.45 ~ 1.14)	0.162	0.67(0.24 ~ 1.86)	0.444	0.628	1.09(0.97 ~ 1.22)	0.133
		≥40	1（Reference)	1.24(0.81 ~ 1.91)	0.328	0.90(0.36 ~ 2.27)	0.823	0.590	1.03(0.94 ~ 1.13)	0.586

	Model 2[b]	<40	1（Reference)	0.73(0.46 ~ 1.16)	0.184	0.72(0.26 ~ 2.01)	0.537	0.725	1.08(0.97 ~ 1.21)	0.182
		≥40	1（Reference)	1.28(0.83 ~ 1.97)	0.266	0.91(0.36 ~ 2.30)	0.841	0.577	1.02(0.93 ~ 1.12)	0.627
	Model 3[c]	<40	1（Reference)	0.74(0.46 ~ 1.18)	0.263	0.78(0.28 ~ 2.16)	0.627	0.819	1.07(0.96 ~ 1.20)	0.231
		≥40	1（Reference)	1.28(0.83 ~ 1.98)	0.263	0.88(0.35 ~ 2.23)	0.791	0.527	1.02(0.93 ~ 1.12)	0.628
	Model 4[d]	<40	1（Reference)	0.79(0.49 ~ 1.26)	0.319	0.94(0.33 ~ 2.63)	0.902	0.953	1.04(0.93 ~ 1.17)	0.442
		≥40	1（Reference)	1.41(0.91 ~ 2.18)	0.129	1.06(0.42 ~ 2.71)	0.901	0.733	1.00(0.91 ~ 1.10)	0.961
Viral hepatitis	Model 1[a]	<40	1（Reference)	0.78(0.49 ~ 1.22)	0.272	0.66(0.24 ~ 1.82)	0.421	0.548	1.07(0.96 ~ 1.19)	0.207
		≥40	1（Reference)	1.70(0.63 ~ 4.58)	0.290	3.34(0.86 ~ 12.91)	0.081	0.164	0.90(0.78 ~ 1.03)	0.125
	Model 2[b]	<40	1（Reference)	0.82(0.52 ~ 1.28)	0.382	0.69(0.25 ~ 1.92)	0.482	0.580	1.06(0.96 ~ 1.17)	0.271
		≥40	1（Reference)	1.82(0.68 ~ 4.91)	0.236	3.59(0.92 ~ 13.99)	0.065	0.145	0.89(0.78 ~ 1.03)	0.110
	Model 3[c]	<40	1（Reference)	0.82(0.52 ~ 1.29)	0.382	0.71(0.26 ~ 1.97)	0.512	0.614	1.06(0.95 ~ 1.17)	0.288
		≥40	1（Reference)	1.96(0.72 ~ 5.33)	0.186	4.39(1.12 ~ 17.18)	0.034	0.098	0.88(0.76 ~ 1.00)	0.056
	Model 4[d]	<40	1（Reference)	0.78(0.49 ~ 1.23)	0.287	0.67(0.24 ~ 1.86)	0.441	0.559	1.07(0.96 ~ 1.19)	0.214
		≥40	1（Reference)	1.92(0.68 ~ 5.40)	0.219	5.31(1.29 ~ 21.85)	0.021	0.071	0.86(0.74 ~ 1.00)	0.050
Autoimmune hepatitis Autoimmune hepatitis	Model 1[a]	<40	1（Reference)	1.11(0.51 ~ 2.43)	0.797	0.61(0.08 ~ 4.64)	0.631	0.590	0.97(0.84 ~ 1.13)	0.696
		≥40	1（Reference)	3.54(0.94 ~ 13.33)	0.062	7.79(1.57 ~ 38.58)	0.012	0.034	0.88(0.75 ~ 1.03)	0.107
	Model 2[b]	<40	1（Reference)	1.07(0.49 ~ 2.35)	0.862	0.59(0.08 ~ 4.51)	0.611	0.582	0.98(0.84 ~ 1.13)	0.746
		≥40	1（Reference)	3.48(0.92 ~ 13.21)	0.066	6.77(1.36 ~ 33.61)	0.019	0.055	0.89(0.76 ~ 1.05)	0.166
	Model 3[c]	<40	1（Reference)	1.04(0.47 ~ 2.28)	0.924	0.56(0.07 ~ 4.27)	0.574	0.555	0.98(0.85 ~ 1.14)	0.818
		≥40	1（Reference)	3.42(0.90 ~ 13.05)	0.072	6.36(1.26 ~ 32.16)	0.025	0.070	0.90(0.76 ~ 1.06)	0.197
	Model 4[d]	<40	1（Reference)	1.06(0.48 ~ 2.34)	0.886	0.58(0.08 ~ 4.47)	0.602	0.575	0.98(0.84 ~ 1.13)	0.758
		≥40	1（Reference)	3.06(0.79 ~ 11.88)	0.107	6.08(1.17 ~ 31.67)	0.032	0.074	0.89(0.75 ~ 1.07)	0.221

[a]Model 1 unadjusted(crude).

[b]Model 2 adjusted for age at baseline, sex, ethnicity, and Townsend deprivation index.

[c]Model 3 adjusted for model 2 plus smoking status, alcohol consumption, and physical activity.

[d]Model 4 adjusted for model 3 plus education, waist circumference, hypertension status, diabetes status, ALT, and HDL.

HR Hazards ratio, CI Confidence interval, avMSE Mean spherical equivalent refractive error, AST Aspartate aminotransferase, ALT Alanine aminotransferase, HDL High-density lipoprotein.

Table S8. Association Between Myopia Status and Liver Disease Incidence Using AST ≥35 U/L as the Threshold

Liver diseases		AST level	Myopia Status				
			Emmetropia	Low/moderate myopia		High myopia	
			HR (95%CI)	HR (95%CI)	P	HR (95%CI)	P
Liver fibrosis and cirrhosis	Model 1	≤35	1（Reference)	1.26(0.88-1.81)	0.210	0.55(0.20-1.50)	0.241
		>35	1（Reference)	1.25(0.86-1.82)	0.233	2.54(1.46-4.42)	0.001
	Model 2	≤35	1（Reference)	1.19(0.83-1.72)	0.341	0.54(0.19-1.48)	0.227
		>35	1（Reference)	1.21(0.83-1.76)	0.315	2.47(1.42-4.31)	0.001
	Model 3	≤35	1（Reference)	1.25(0.87-1.80)	0.229	0.59(0.22-1.64)	0.315
		>35	1（Reference)	1.20(0.82-1.74)	0.348	2.56(1.47-4.48)	0.001
	Model 4	≤35	1（Reference)	1.28(0.89-1.85)	0.187	0.65(0.23-1.79)	0.402
		>35	1（Reference)	1.30(0.89-1.89)	0.178	2.96(1.68-5.20)	<.001
Non-alcoholic fatty liver disease	Model 1	≤35	1（Reference)	0.87(0.74-1.01)	0.073	0.83(0.60-1.15)	0.262
		>35	1（Reference)	1.02(0.79-1.32)	0.858	1.05(0.63-1.77)	0.842
	Model 2	≤35	1（Reference)	0.86(0.74-1.01)	0.070	0.83(0.60-1.15)	0.253
		>35	1（Reference)	1.02(0.79-1.32)	0.885	1.02(0.61-1.72)	0.938
	Model 3	≤35	1（Reference)	0.89(0.76-1.04)	0.145	0.88(0.63-1.22)	0.440
		>35	1（Reference)	0.99(0.76-1.27)	0.916	1.00(0.60-1.69)	0.987
	Model 4	≤35	1（Reference)	0.93(0.79-1.08)	0.337	0.99(0.71-1.37)	0.932
		>35	1（Reference)	1.06(0.82-1.37)	0.678	1.13(0.67-1.91)	0.646
Alcoholic liver disease	Model 1	≤35	1（Reference)	0.76(0.45-1.26)	0.283	0.61(0.19-1.99)	0.416
		>35	1（Reference)	1.12(0.76-1.67)	0.559	1.00(0.43-2.32)	0.993
	Model 2	≤35	1（Reference)	0.75(0.45-1.26)	0.278	0.65(0.20-2.11)	0.476
		>35	1（Reference)	1.12(0.75-1.66)	0.578	1.01(0.43-2.34)	0.990
	Model 3	≤35	1（Reference)	0.78(0.47-1.31)	0.354	0.72(0.22-2.35)	0.592
		>35	1（Reference)	1.10(0.74-1.63)	0.652	1.02(0.44-2.37)	0.967
	Model 4	≤35	1（Reference)	0.83(0.49-1.40)	0.486	0.83(0.25-2.70)	0.751
		>35	1（Reference)	1.23(0.83-1.84)	0.308	1.14(0.49-2.69)	0.758

Model 1 unadjusted(crude).

Model 2 adjusted for age at baseline, sex, ethnicity, and Townsend deprivation index.

Model 3 adjusted for model 2 plus smoking status, alcohol consumption, and physical activity.

Model 4 adjusted for model 3 plus education, waist circumference, hypertension status, diabetes status, ALT, and HDL.

HR Hazards ratio, CI Confidence interval, avMSE Mean spherical equivalent refractive error, AST Aspartate aminotransferase, ALT Alanine aminotransferase, HDL High-density lipoprotein.

Table S9. Subgroup analyses of the associations between myopia status and the risk of liver fibrosis and cirrhosis in the UKB cohort

Variable	Level	Count	Percent	Myopia	HR	CI_95_Lower	CI_95_Upper	P_Value	P for interaction
Overall		3538	100	Low/moderate myopia	1.32	0.88	1.99	0.185	
				High myopia	2.64	1.44	4.85	0.002	
Age	≤65 years	3160	89.3	Low/moderate myopia	1.48	0.94	2.32	0.088	0.319
				High myopia	3.03	1.55	5.89	0.001	
	＞65 years	378	10.7	Low/moderate myopia	0.86	0.27	2.73	0.803	
				High myopia	1.58	0.31	7.92	0.580	
Sex	Male	2355	66.6	Low/moderate myopia	1.25	0.76	2.08	0.381	0.933
				High myopia	2.63	1.23	5.61	0.013	
	Female	1183	33.4	Low/moderate myopia	1.22	0.60	2.48	0.580	
				High myopia	2.37	0.84	6.71	0.104	
Waist	>80 cm	3086	87.2	Low/moderate myopia	1.43	0.94	2.18	0.099	0.199
				High myopia	2.55	1.33	4.91	0.005	
	≤80 cm	452	12.8	Low/moderate myopia	0.42	0.04	4.10	0.458	
				High myopia	9.38	1.21	72.52	0.032	
Education	Below high school	1256	35.5	Low/moderate myopia	1.02	0.48	2.19	0.953	0.954
				High myopia	2.41	0.90	6.47	0.080	
	High school	417	11.8	Low/moderate myopia	1.63	0.29	9.20	0.583	
				High myopia	2.59	0.20	34.10	0.469	
	College or above	1865	52.7	Low/moderate myopia	1.40	0.83	2.35	0.204	
				High myopia	2.69	1.17	6.19	0.020	
Ethnicity	White	2789	78.8	Low/moderate myopia	1.00	0.64	1.57	0.991	0.007
				High myopia	2.10	1.10	4.03	0.025	
	Non-white	749	21.2	Low/moderate myopia	6.93	1.46	32.79	0.015	
				High myopia	22.42	2.63	190.88	0.004	
Smoke group	No	1873	52.9	Low/moderate myopia	1.62	0.81	3.25	0.171	0.518
				High myopia	1.93	0.62	5.98	0.257	

	Yes	1665	47.1	Low/moderate myopia	1.17	0.70	1.96	0.558	
				High myopia	3.09	1.50	6.38	0.002	
Alcohol group	Non-drinker	313	8.8	Low/moderate myopia	9.49	2.08	43.27	0.004	0.007
				High myopia	5.21	0.42	64.97	0.200	
	1-5 drinks/month	800	22.6	Low/moderate myopia	1.20	0.47	3.08	0.699	
				High myopia	2.10	0.44	10.00	0.352	
	5-10 drinks/month	746	21.1	Low/moderate myopia	1.34	0.52	3.45	0.549	
				High myopia	5.73	1.87	17.53	0.002	
	10+ drinks/month	1679	47.5	Low/moderate myopia	0.72	0.38	1.37	0.321	
				High myopia	1.48	0.56	3.89	0.426	
Townsend deprivation index	Low	800	22.6	Low/moderate myopia	1.34	0.56	3.16	0.511	0.129
				High myopia	NA	NA	NA	NA	
	Moderate	1723	48.7	Low/moderate myopia	1.18	0.64	2.18	0.587	
				High myopia	2.85	1.24	6.54	0.013	
	High	1015	28.7	Low/moderate myopia	1.57	0.74	3.32	0.235	
				High myopia	4.59	1.64	12.84	0.004	
Physical activity	Low	647	18.3	Low/moderate myopia	1.15	0.53	2.50	0.721	0.929
				High myopia	3.80	1.25	11.52	0.019	
	Moderate	1365	38.6	Low/moderate myopia	1.47	0.79	2.75	0.222	
				High myopia	2.22	0.87	5.67	0.096	
	High	1526	43.1	Low/moderate myopia	1.12	0.50	2.48	0.788	
				High myopia	2.04	0.58	7.20	0.268	
Diabetes status	No	3174	89.7	Low/moderate myopia	0.94	0.56	1.58	0.826	0.078
				High myopia	2.78	1.45	5.35	0.002	
	Yes	364	10.3	Low/moderate myopia	2.05	1.00	4.20	0.051	
				High myopia	0.77	0.10	6.18	0.809	
Hypertension status	No	2286	64.6	Low/moderate myopia	0.95	0.52	1.76	0.875	0.604
				High myopia	2.28	1.02	5.13	0.045	
	Yes	1252	35.4	Low/moderate myopia	1.58	0.90	2.78	0.112	
				High myopia	3.08	1.22	7.80	0.018	

HR Hazards ratio, CI Confidence interval, NA not applicable.

Table S10. Subgroup analyses of the associations between myopia status and the risk of viral hepatitis in the UKB cohort

Variable	Level	Count	Percent	Myopia Status	HR	CI_95_Lower	CI_95_Upper	P_Value	P for interaction
Overall		3538	100	Low/moderate myopia	1.60	0.59	4.30	0.355	
				High myopia	4.03	1.02	15.92	0.046	
Age	≤65 years	3160	89.3	Low/moderate myopia	1.60	0.57	4.50	0.372	0.667
				High myopia	4.63	1.15	18.67	0.031	
	＞65 years	378	10.7	Low/moderate myopia	NA	NA	NA	NA	
				High myopia	NA	NA	NA	NA	
Sex	Male	2355	66.6	Low/moderate myopia	0.91	0.26	3.12	0.878	0.546
				High myopia	1.91	0.21	17.03	0.562	
	Female	1183	33.4	Low/moderate myopia	4.78	0.72	31.49	0.104	
				High myopia	15.43	1.53	155.22	0.020	
Waist	>80 cm	3086	87.2	Low/moderate myopia	1.10	0.39	3.12	0.852	0.041
				High myopia	1.14	0.14	9.34	0.906	
	≤80 cm	452	12.8	Low/moderate myopia	NA	NA	NA	NA	
				High myopia	NA	NA	NA	NA	
Education	Below high school	1256	35.5	Low/moderate myopia	1.93	0.47	8.00	0.363	0.837
				High myopia	4.14	0.67	25.60	0.126	
	High school	417	11.8	Low/moderate myopia	NA	NA	NA	NA	
				High myopia	NA	NA	NA	NA	
	College or above	1865	52.7	Low/moderate myopia	1.02	0.18	5.93	0.979	
				High myopia	3.31	0.33	32.77	0.306	
Ethnicity	White	2789	78.8	Low/moderate myopia	0.90	0.25	3.27	0.871	0.187
				High myopia	1.21	0.14	10.34	0.864	
	Non-white	749	21.2	Low/moderate myopia	3.17	0.85	11.80	0.086	
				High myopia	24.94	5.15	120.91	<0.001	
Smoke group	No	1873	52.9	Low/moderate myopia	1.34	0.17	10.81	0.785	0.421
				High myopia	NA	NA	NA	NA	

	Yes	1665	47.1	Low/moderate myopia	1.65	0.54	5.04	0.379	
				High myopia	5.33	1.27	22.40	0.022	
Alcohol group	Non-drinker	313	8.8	Low/moderate myopia	0.89	0.12	6.60	0.909	0.278
				High myopia	NA	NA	NA	NA	
	1-5 drinks/month	800	22.6	Low/moderate myopia	2.68	0.24	29.60	0.420	
				High myopia	2.82	0.19	40.76	0.447	
	5-10 drinks/month	746	21.1	Low/moderate myopia	NA	NA	NA	NA	
				High myopia	NA	NA	NA	NA	
	10+ drinks/month	1679	47.5	Low/moderate myopia	0.49	0.09	2.62	0.401	
				High myopia	1.37	0.15	12.30	0.778	
Townsend deprivation index	Low	800	22.6	Low/moderate myopia	NA	NA	NA	NA	0.247
				High myopia	NA	NA	NA	NA	
	Moderate	1723	48.7	Low/moderate myopia	0.74	0.16	3.31	0.691	
				High myopia	2.50	0.27	22.69	0.416	
	High	1015	28.7	Low/moderate myopia	4.20	0.82	21.53	0.085	
				High myopia	9.30	1.18	73.53	0.035	
Physical activity	Low	647	18.3	Low/moderate myopia	NA	NA	NA	NA	0.879
				High myopia	NA	NA	NA	NA	
	Moderate	1365	38.6	Low/moderate myopia	1.60	0.26	9.98	0.615	
				High myopia	3.60	0.31	41.77	0.306	
	High	1526	43.1	Low/moderate myopia	1.33	0.35	5.09	0.681	
				High myopia	5.73	1.01	32.56	0.049	
Diabetes status	No	3174	89.7	Low/moderate myopia	1.29	0.42	3.91	0.657	0.371
				High myopia	3.87	0.96	15.53	0.057	
	Yes	364	10.3	Low/moderate myopia	NA	NA	NA	NA	
				High myopia	NA	NA	NA	NA	
Hypertension status	No	2286	64.6	Low/moderate myopia	1.02	0.24	4.40	0.977	0.115
				High myopia	4.87	1.11	21.44	0.036	
	Yes	1252	35.4	Low/moderate myopia	1.92	0.46	8.00	0.371	
				High myopia	NA	NA	NA	NA	

HR Hazards ratio, CI Confidence interval, NA = Not available due to insufficient event numbers (<5 cases) for stable model estimation.

Table S11. Subgroup analyses of the associations between myopia status and the risk of autoimmune hepatitis in the UKB cohort

Variable	Level	Count	Percent	Myopia Status	HR	CI_95_Lower	CI_95_Upper	P_Value	P for interaction
Overall		3538	100	Low/moderate myopia	3.06	0.79	11.88	0.107	
				High myopia	6.08	1.17	31.67	0.032	
Age	≤65 years	3160	89.3	Low/moderate myopia	3.36	0.65	17.24	0.147	0.370
				High myopia	10.37	1.63	65.85	0.013	
	＞65 years	378	10.7	Low/moderate myopia	NA	NA	NA	NA	
				High myopia	NA	NA	NA	NA	
Sex	Male	2355	66.6	Low/moderate myopia	NA	NA	NA	NA	0.017
				High myopia	NA	NA	NA	NA	
	Female	1183	33.4	Low/moderate myopia	3.02	0.77	11.84	0.113	
				High myopia	2.12	0.22	20.83	0.520	
Waist	>80 cm	3086	87.2	Low/moderate myopia	2.54	0.62	10.40	0.195	0.430
				High myopia	3.75	0.60	23.67	0.159	
	≤80 cm	452	12.8	Low/moderate myopia	NA	NA	NA	0.946	
				High myopia	NA	NA	NA	0.880	
Education	Below high school	1256	35.5	Low/moderate myopia	NA	NA	NA	NA	0.181
				High myopia	NA	NA	NA	NA	
	High school	417	11.8	Low/moderate myopia	1.05	0.85	1.29	0.641	
				High myopia	1.00	0.68	1.47	0.997	
	College or above	1865	52.7	Low/moderate myopia	3.21	0.82	12.51	0.092	
				High myopia	2.17	0.22	21.29	0.507	
Ethnicity	White	2789	78.8	Low/moderate myopia	4.99	1.03	24.05	0.045	0.126
				High myopia	6.38	0.87	46.61	0.068	
	Non-white	749	21.2	Low/moderate myopia	NA	NA	NA	0.999	
				High myopia	NA	NA	NA	0.814	
Smoke group	No	1873	52.9	Low/moderate myopia	5.30	0.57	49.57	0.143	0.148
				High myopia	19.98	1.92	208.29	0.012	
	Yes	1665	47.1	Low/moderate myopia	5.53	1.01	30.28	0.049	

				High myopia	NA	NA	NA	NA	
Alcohol group	Non-drinker	313	8.8	Low/moderate myopia	NA	NA	NA	NA	0.208
				High myopia	NA	NA	NA	NA	
	1-5 drinks/month	800	22.6	Low/moderate myopia	NA	NA	NA	NA	
				High myopia	NA	NA	NA	NA	
	5-10 drinks/month	746	21.1	Low/moderate myopia	0.35	0.02	5.00	0.437	
				High myopia	2.56	0.18	35.91	0.486	
	10+ drinks/month	1679	47.5	Low/moderate myopia	NA	NA	NA	NA	
				High myopia	NA	NA	NA	NA	
Townsend deprivation index	Low	800	22.6	Low/moderate myopia	NA	NA	NA	NA	0.607
				High myopia	NA	NA	NA	NA	
	Moderate	1723	48.7	Low/moderate myopia	1.67	0.27	10.41	0.584	
				High myopia	5.47	0.72	41.37	0.100	
	High	1015	28.7	Low/moderate myopia	5.77	0.96	34.80	0.056	
				High myopia	5.13	0.57	46.40	0.146	
Physical activity	Low	647	18.3	Low/moderate myopia	0.22	0.02	1.93	0.171	0.069
				High myopia	5.78	0.96	34.60	0.055	
	Moderate	1365	38.6	Low/moderate myopia	8.16	0.84	79.08	0.070	
				High myopia	NA	NA	NA	NA	
	High	1526	43.1	Low/moderate myopia	NA	NA	NA	NA	
				High myopia	NA	NA	NA	NA	
Diabetes status	No	3174	89.7	Low/moderate myopia	3.18	0.82	12.24	0.093	1.000
				High myopia	6.69	1.31	34.30	0.023	
	Yes	364	10.3	Low/moderate myopia	1.12	0.89	1.40	0.321	
				High myopia	0.71	0.40	1.27	0.251	
Hypertension status	No	2286	64.6	Low/moderate myopia	1.16	0.22	5.98	0.860	0.071
				High myopia	10.87	2.43	48.57	0.002	
	Yes	1252	35.4	Low/moderate myopia	9.98	1.20	82.92	0.033	
				High myopia	NA	NA	NA	NA	

HR Hazards ratio, CI Confidence interval.

NA denotes not available due to insufficient event numbers (<5 cases) for stable model estimation.

Table S12. Selection of blood biomarkers as potential mediators between myopia status and incident liver diseases

Blood Biomarkers	Associations between myopia status and biomarkers			Associations between biomarkers and incident liver fibrosis and cirrhosis			Associations between biomarkers and incident viral hepatitis			Associations between biomarkers and incident autoimmune hepatitis		
	Beta[a] (95% CI)	P	FDR	HR[b] (95% CI)	P	FDR	HR[b] (95% CI)	P	FDR	HR[b] (95% CI)	P	FDR
Inflammatory-related biomarkers												
Leukocyte count, 10^9 cells/L	0.62(0.25 ~ 0.99)	0.001	0.008	1.01(0.95 ~ 1.07)	0.722	0.916	1.02(0.90 ~ 1.16)	0.709	0.709	0.78(0.56 ~ 1.09)	0.146	0.327
Neutrophil count, 10^9 cells/L	0.17(-0.02 ~ 0.37)	0.076	0.196	0.99(0.87 ~ 1.13)	0.918	0.918	0.85(0.61 ~ 1.20)	0.364	0.481	0.73(0.46 ~ 1.14)	0.163	0.327
Monocyte count, 10^9 cells/L	0.01(-0.03 ~ 0.05)	0.574	0.730	1.24(0.82 ~ 1.87)	0.307	0.613	1.27(0.64 ~ 3.40)	0.368	0.481	0.96(0.13 ~ 6.84)	0.965	0.965
Lymphocyte count, 10^9 cells/L	0.42(0.14 ~ 0.71)	0.004	0.916	1.01 (0.94 ~ 1.09)	0.764	0.916	1.04(0.97 ~ 1.12)	0.281	0.481	0.65(0.27 ~ 1.56)	0.333	0.499
C reactive protein, mg/L	0.89(-0.64 ~ 0.40)	0.007	0.020	1.04 (1.02 ~ 1.05)	4.10E-05	0.0001	1.02 (0.97 ~ 1.07)	0.401	0.481	0.94(0.79 ~ 1.10)	0.432	0.519
Platelet count, 10^9 cells/L	-6.90(-14.37 ~ 0.56)	0.070	0.191	0.99 (0.98 ~ 0.99)	1.51E-12	9.06E-12	0.99 (0.98 ~ 1.00)	0.075	0.452	0.99 (0.98 ~ 1.00)	0.130	0.327
Complex Inflammation Indicators												
INFLA	0.20(-0.57 ~ 0.96)	0.618	0.844	0.99(0.95 ~ 1.02)	0.421	0.590	0.94(0.86 ~ 1.02)	0.121	0.214	0.95(0.86 ~ 1.05)	0.295	0.517
MHR	0.03(-0.01 ~ 0.06)	0.133	0.297	1.36(1.01 ~ 1.85)	0.044	0.102	1.75(1.02 ~ 2.99)	0.043	0.214	0.92(0.08 ~ 10.13)	0.948	0.948
NLR	0.18(0.01 ~ 0.38)	0.068	0.181	1.02(0.90 ~ 1.15)	0.778	0.778	1.05(0.85 ~ 1.30)	0.655	0.655	0.87(0.47 ~ 1.62)	0.663	0.861
PLR	1.95(-5.34 ~ 9.24)	0.600	0.838	0.99(0.98 ~ 1.00)	<0.001	0.002	0.99(0.98 ~ 1.00)	0.122	0.214	1.00(0.99 ~ 1.01)	0.738	0.861
LMR	0.11(-0.23 ~ 0.44)	0.528	0.769	0.97(0.87 ~ 1.07)	0.512	0.597	1.05(0.91 ~ 1.21)	0.521	0.608	0.81(0.56 ~ 1.17)	0.269	0.517

[a]Models were adjusted for age, sex, education, Townsend deprivation index, waist, smoking status, drinking status, physical activity, hypertension, diabetes, ALT, HDL.

[b]Models were adjusted for age, sex, education, Townsend deprivation index, waist, smoking status, drinking status, physical activity, hypertension, diabetes, ALT, HDL, and myopia status.

Bolded indicated that biomarkers may be potential mediators in the association between high myopia and the risk of liver diseases.

FDR False discovery rate, PM Proportion mediated, HR Hazards ratio, INFLA The low-grade chronic inflammation score, MHR Monocyte to high-density lipoprotein cholesterol ratio, NLR Neutrophil-to-lymphocyte ratio, PLR Platelet-to-lymphocyte ratio, LMR Lymphocyte-to-monocyte ratio, CI Confidence interval, ALT Alanine aminotransferase, HDL High-density lipoprotein.

Table S13. Selection of metabolites as potential mediators between high myopia and incident liver fibrosis and cirrhosis

Metabolites	HR[a]	CI_lower	CI_upper	P	FDR	Beta[b]	CI_lower	CI_upper	P	FDR
Total Cholesterol	0.6421	0.4575	0.9012	0.0104	0.0189	0.0607	-0.0928	0.2143	0.4380	0.7859
Total Cholesterol Minus HDL-C	0.6421	0.4575	0.9012	0.0104	0.0189	0.0607	-0.0928	0.2143	0.4380	0.7859
Remnant Cholesterol (Non-HDL, Non-LDL -Cholesterol)	0.6340	0.3301	1.2178	0.1712	0.2061	0.0399	-0.0364	0.1163	0.3052	0.7818
VLDL Cholesterol	0.3806	0.1265	1.1456	0.0858	0.1125	0.0149	-0.0313	0.0611	0.5262	0.8082
Clinical LDL Cholesterol	0.5486	0.3681	0.8176	0.0032	0.0069	0.0625	-0.0702	0.1951	0.3558	0.7818
LDL Cholesterol	0.2921	0.1502	0.5680	0.0003	0.0009	0.0208	-0.0593	0.1009	0.6108	0.8311
HDL Cholesterol	NA	NA	NA	NA	NA	3.61E-17	-2.29E-17	9.51E-17	0.2306	0.7030
Total Triglycerides	0.5061	0.3132	0.8179	0.0054	0.0102	-0.0415	-0.1638	0.0808	0.5062	0.7995
Triglycerides in VLDL	0.3254	0.1737	0.6096	0.0005	0.0014	-0.0407	-0.1414	0.0600	0.4283	0.7859
Triglycerides in LDL	0.9673	0.0075	125.3955	0.9893	0.9893	0.0015	-0.0078	0.0107	0.7550	0.8786
Triglycerides in HDL	1.2351	0.0173	87.9291	0.9227	0.9378	-0.0038	-0.0142	0.0065	0.4651	0.7859
Total Phospholipids in Lipoprotein Particles	0.4351	0.2218	0.8533	0.0155	0.0263	0.0033	-0.0715	0.0781	0.9318	0.9628
Phospholipids in VLDL	0.1643	0.0363	0.7434	0.0190	0.0317	0.0014	-0.0338	0.0367	0.9375	0.9646
Phospholipids in LDL	0.0271	0.0035	0.2082	0.0005	0.0015	0.0103	-0.0157	0.0363	0.4360	0.7859
Phospholipids in HDL	0.4327	0.0551	3.3954	0.4255	0.4753	-0.0158	-0.0385	0.0069	0.1713	0.6370
Total Esterified Cholesterol	0.4951	0.3033	0.8081	0.0049	0.0093	0.0401	-0.0669	0.1471	0.4624	0.7859
Cholesteryl Esters in VLDL	0.3361	0.0519	2.1782	0.2528	0.2957	0.0121	-0.0144	0.0386	0.3720	0.7818
Cholesteryl Esters in LDL	0.1744	0.0714	0.4262	0.0001	0.0005	0.0114	-0.0482	0.0710	0.7085	0.8606
Cholesteryl Esters in HDL	2.94E-08	9.57E-13	0.0009	0.0010	0.0026	-0.0006	-0.0041	0.0029	0.7468	0.8786

Total Free Cholesterol	0.3361	0.1134	0.9959	0.0491	0.0685	0.0206	-0.0264	0.0676	0.3897	0.7844
Free Cholesterol in VLDL	0.0417	0.0031	0.5595	0.0165	0.0278	0.0029	-0.0176	0.0233	0.7847	0.8963
Free Cholesterol in LDL	0.0280	0.0024	0.3235	0.0042	0.0083	0.0094	-0.0121	0.0310	0.3910	0.7844
Free Cholesterol in HDL	3.39E+07	1.10E+03	1.04E+12	0.0010	0.0026	0.0006	-0.0029	0.0041	0.7477	0.8786
Total Lipids in Lipoprotein Particles	0.7758	0.6551	0.9188	0.0033	0.0069	0.0225	-0.2875	0.3326	0.8866	0.9574
Total Lipids in VLDL	0.6036	0.4338	0.8400	0.0027	0.0060	-0.0243	-0.1963	0.1477	0.7814	0.8963
Total Lipids in LDL	0.4348	0.2699	0.7004	0.0006	0.0017	0.0326	-0.0781	0.1432	0.5636	0.8181
Total Lipids in HDL	0.6827	0.1648	2.8278	0.5986	0.6455	-0.0197	-0.0520	0.0127	0.2329	0.7030
Total Concentration of Lipoprotein Particles	7.14E-250	1.50e-321	3.40E-178	9.58E-12	1.25E-10	-0.0001	-0.0004	0.0001	0.3390	0.7818
Concentration of VLDL Particles	0.00E+00	0.00E+00	1.40E+306	0.0848	0.1125	2.08E-06	-6.61E-06	1.08E-05	0.6392	0.8421
Concentration of LDL Particles	0.00E+00	0.00E+00	5.48E-89	0.0179	0.0300	2.43E-05	-2.91E-05	0.0001	0.3730	0.7818
Concentration of HDL Particles	4.28E-288	0.00E+00	1.99E-210	4.11E-13	7.28E-12	-0.0002	-0.0004	0.0001	0.1531	0.6370
Average Diameter for VLDL Particles	0.5262	0.4225	0.6554	9.94E-09	7.95E-08	-0.2104	-0.4186	-0.0022	0.0476	0.5392
Average Diameter for LDL Particles	1.8324	0.0890	37.7405	0.6948	0.7395	0.0060	-0.0099	0.0219	0.4597	0.7859
Average Diameter for HDL Particles	564.6632	148.3930	2148.6494	1.50E-20	3.72E-18	0.0163	-0.0064	0.0390	0.1589	0.6370
Phosphoglycerides	0.3832	0.1704	0.8617	0.0203	0.0336	-0.0114	-0.0724	0.0496	0.7145	0.8637
Triglycerides to Phosphoglycerides ratio	0.1084	0.0239	0.4921	0.0040	0.0080	-0.0201	-0.0560	0.0158	0.2720	0.7529
Total Cholines	0.3777	0.1623	0.8789	0.0238	0.0386	-0.0041	-0.0625	0.0543	0.8908	0.9574
Phosphatidylcholines	0.4420	0.1843	1.0604	0.0675	0.0924	-0.0088	-0.0637	0.0461	0.7533	0.8786
Sphingomyelins	0.0203	0.0001	3.3360	0.1344	0.1683	0.0066	-0.0032	0.0164	0.1875	0.6370

Apolipoprotein B	0.2497	0.0640	0.9748	0.0459	0.0665	0.0194	-0.0181	0.0569	0.3106	0.7818
Apolipoprotein A1	0.0045	0.0002	0.0927	0.0005	0.0014	-0.0142	-0.0311	0.0027	0.1000	0.6370
Apolipoprotein B to Apolipoprotein A1 ratio	2.3021	0.4166	12.7215	0.3391	0.3875	0.0360	0.0066	0.0654	0.0165	0.4563
Total Fatty Acids	0.8558	0.7681	0.9534	0.0047	0.0092	-0.0883	-0.5916	0.4149	0.7307	0.8747
Degree of Unsaturation	0.0063	0.0002	0.1661	0.0024	0.0056	0.0046	-0.0100	0.0191	0.5374	0.8109
Omega-3 Fatty Acids	0.0205	0.0042	0.0993	1.39E-06	0.0000	0.0014	-0.0416	0.0444	0.9480	0.9691
Omega-6 Fatty Acids	0.4703	0.2956	0.7482	0.0014	0.0035	-0.0039	-0.1238	0.1160	0.9497	0.9691
Polyunsaturated Fatty Acids	0.4375	0.2942	0.6506	4.45E-05	0.0002	-0.0024	-0.1450	0.1401	0.9733	0.9812
Monounsaturated Fatty Acids	0.7092	0.5269	0.9546	0.0234	0.0382	-0.0289	-0.2109	0.1530	0.7551	0.8786
Saturated Fatty Acids	0.7577	0.5871	0.9778	0.0329	0.0498	-0.0570	-0.2638	0.1499	0.5893	0.8311
Linoleic Acid	0.6089	0.3914	0.9474	0.0278	0.0437	0.0059	-0.1171	0.1290	0.9250	0.9597
Docosahexaenoic Acid	0.0002	3.31E-06	0.0178	0.0001	0.0005	0.0020	-0.0134	0.0175	0.7985	0.9079
Omega-3 Fatty Acids to Total Fatty Acids percentage	0.5815	0.4631	0.7300	3.00E-06	1.69E-05	0.0167	-0.2773	0.3107	0.9112	0.9597
Omega-6 Fatty Acids to Total Fatty Acids percentage	1.0487	0.9798	1.1225	0.1705	0.2061	0.2870	-0.4182	0.9921	0.4248	0.7859
Polyunsaturated Fatty Acids to Total Fatty Acids percentage	0.9900	0.9291	1.0550	0.7575	0.7927	0.3037	-0.4158	1.0231	0.4078	0.7859
Monounsaturated Fatty Acids to Total Fatty Acids percentage	0.9632	0.8704	1.0659	0.4687	0.5166	-0.1320	-0.6069	0.3429	0.5857	0.8311
Saturated Fatty Acids to Total Fatty Acids percentage	1.0808	0.9697	1.2047	0.1604	0.1959	-0.1717	-0.5526	0.2092	0.3768	0.7818
Linoleic Acid to Total Fatty Acids percentage	1.0604	0.9814	1.1458	0.1378	0.1701	0.3193	-0.2947	0.9334	0.3079	0.7818

Docosahexaenoic Acid to Total Fatty Acids percentage	0.5571	0.3488	0.8898	0.0143	0.0247	0.0204	-0.1073	0.1481	0.7541	0.8786
Polyunsaturated Fatty Acids to Monounsaturated Fatty Acids ratio	1.0129	0.4400	2.3316	0.9760	0.9800	0.0256	-0.0328	0.0839	0.3902	0.7844
Omega-6 Fatty Acids to Omega-3 Fatty Acids ratio	1.0925	1.0634	1.1225	1.35E-10	0.0000	0.3354	-0.5167	1.1875	0.4402	0.7859
Alanine	0.5671	0.0293	10.9738	0.7075	0.7498	-0.0152	-0.0302	-0.0002	0.0466	0.5392
Glutamine	0.1176	0.0056	2.4600	0.1677	0.2038	0.0036	-0.0127	0.0200	0.6619	0.8532
Glycine	0.5957	0.0039	91.6009	0.8402	0.8646	-0.0011	-0.0108	0.0086	0.8210	0.9209
Histidine	2.34E-07	7.02E-18	7829.2626	0.2169	0.2574	-0.0005	-0.0024	0.0014	0.6283	0.8366
Total Concentration of Branched-Chain Amino Acids (Leucine + Isoleucine + Valine)	0.0341	0.0012	0.9593	0.0472	0.0669	-0.0044	-0.0191	0.0103	0.5561	0.8145
Isoleucine	0.0001	1.07E-11	302.9959	0.2161	0.2574	0.0002	-0.0028	0.0033	0.8928	0.9574
Leucine	4.96E-05	2.01E-09	1.2267	0.0548	0.0755	-0.0011	-0.0059	0.0038	0.6599	0.8532
Valine	0.0008	1.14E-06	0.5097	0.0305	0.0467	-0.0035	-0.0111	0.0040	0.3558	0.7818
Phenylalanine	1.44E+07	5.57E+02	3.74E+11	0.0015	0.0035	-0.0015	-0.0038	0.0007	0.1883	0.6370
Tyrosine	2.02E+15	3.39E+09	1.21E+21	2.06E-07	1.42E-06	0.0003	-0.0025	0.0031	0.8454	0.9398
Glucose	1.0136	0.8759	1.1728	0.8563	0.8775	0.1021	-0.1151	0.3194	0.3565	0.7818
Lactate	0.7712	0.6258	0.9503	0.0148	0.0253	-0.1872	-0.4267	0.0523	0.1255	0.6370
Pyruvate	0.6280	0.0006	712.6321	0.8969	0.9153	-0.0004	-0.0070	0.0063	0.9159	0.9597
Citrate	1.57E+14	3.74E+06	6.57E+21	0.0003	0.0009	0.0005	-0.0020	0.0030	0.6991	0.8606
3-Hydroxybutyrate	23.3653	2.0173	270.6311	0.0117	0.0207	0.0071	-0.0051	0.0193	0.2529	0.7322

Acetate	269.0292	0.4195	1.73E+05	0.0898	0.1166	-0.0007	-0.0031	0.0017	0.5531	0.8145
Acetoacetate	1.43E+07	1.3994	1.47E+14	0.0454	0.0663	0.0011	-0.0011	0.0032	0.3221	0.7818
Acetone	2.06E+23	8.85E+10	4.79E+35	0.0002	0.0008	0.0001	-0.0010	0.0012	0.9169	0.9597
Creatinine	0.0000	0.0000	0.0019	0.0123	0.0215	-0.0016	-0.0039	0.0006	0.1604	0.6370
Albumin	0.8313	0.7751	0.8916	2.31E-07	0.0000	-0.4367	-1.0891	0.2157	0.1894	0.6370
Glycoprotein Acetyls	0.0069	0.0005	0.0882	0.0001	0.0005	0.0011	-0.0207	0.0229	0.9234	0.9597
Concentration of Chylomicrons and Extremely Large VLDL Particles	NA	NA	NA	NA	0.0602	0.0000	0.0000	0.0000	0.4815	0.7888
Total Lipids in Chylomicrons and Extremely Large VLDL	0.2269	0.0578	0.8907	0.0335	0.0504	-0.0164	-0.0586	0.0258	0.4451	0.7859
Phospholipids in Chylomicrons and Extremely Large VLDL	0.0001	2.16E-08	0.8975	0.0472	0.0669	-0.0022	-0.0086	0.0042	0.4942	0.7939
Cholesterol in Chylomicrons and Extremely Large VLDL	0.0012	1.50E-06	0.8933	0.0462	0.0667	-0.0021	-0.0104	0.0063	0.6250	0.8366
Cholesteryl Esters in Chylomicrons and Extremely Large VLDL	4.89E-06	2.70E-11	0.8864	0.0478	0.0669	-0.0009	-0.0055	0.0036	0.6932	0.8606
Free Cholesterol in Chylomicrons and Extremely Large VLDL	4.48E-07	2.35E-13	0.8524	0.0475	0.0669	-0.0012	-0.0050	0.0027	0.5531	0.8145
Triglycerides in Chylomicrons and Extremely Large VLDL	0.0967	0.0117	0.7990	0.0301	0.0464	-0.0121	-0.0400	0.0158	0.3938	0.7844
Concentration of Very Large VLDL Particles	NA	NA	NA	NA	0.0030	0.0000	0.0000	0.0000	0.5134	0.8006

Total Lipids in Very Large VLDL	0.0188	0.0019	0.1914	0.0008	0.0021	-0.0098	-0.0365	0.0170	0.4743	0.7873
Phospholipids in Very Large VLDL	2.28E-08	1.80E-13	0.0029	0.0033	0.0069	-0.0013	-0.0064	0.0037	0.6104	0.8311
Cholesterol in Very Large VLDL	6.04E-08	1.25E-12	0.0029	0.0025	0.0057	-0.0005	-0.0058	0.0048	0.8536	0.9444
Cholesteryl Esters in Very Large VLDL	7.95E-15	5.29E-24	1.20E-05	0.0026	0.0058	3.82E-05	-0.0026	0.0027	0.9772	0.9812
Free Cholesterol in Very Large VLDL	2.12E-14	1.31E-23	3.43E-05	0.0036	0.0074	-0.0005	-0.0033	0.0022	0.7032	0.8606
Triglycerides in Very Large VLDL	0.0011	2.26E-05	0.0488	0.0005	0.0014	-0.0080	-0.0248	0.0089	0.3546	0.7818
Concentration of Large VLDL Particles	NA	NA	NA	NA	0.0009	-3.17E-07	-1.41E-06	7.74E-07	0.5684	0.8181
Total Lipids in Large VLDL	0.0203	0.0033	0.1267	2.98E-05	0.0001	-0.0122	-0.0472	0.0228	0.4939	0.7939
Phospholipids in Large VLDL	6.56E-07	2.33E-10	0.0018	0.0004	0.0014	-0.0021	-0.0097	0.0055	0.5828	0.8311
Cholesterol in Large VLDL	9.31E-06	1.29E-08	0.0067	0.0006	0.0016	-0.0007	-0.0094	0.0081	0.8811	0.9574
Cholesteryl Esters in Large VLDL	2.73E-09	5.20E-15	0.0014	0.0033	0.0069	0.0004	-0.0038	0.0046	0.8572	0.9444
Free Cholesterol in Large VLDL	1.31E-11	3.18E-17	5.43E-06	0.0001	0.0005	-0.0011	-0.0057	0.0036	0.6585	0.8532
Triglycerides in Large VLDL	0.0002	5.80E-06	0.0076	3.69E-06	1.95E-05	-0.0094	-0.0287	0.0099	0.3381	0.7818
Concentration of Medium VLDL Particles	NA	NA	NA	NA	0.0029	0.0000	0.0000	0.0000	0.6952	0.8606
Total Lipids in Medium VLDL	0.0566	0.0137	0.2346	0.0001	0.0003	0.0007	-0.0394	0.0407	0.9744	0.9812
Phospholipids in Medium VLDL	0.0001	3.34E-07	0.0511	0.0033	0.0069	0.0022	-0.0068	0.0112	0.6316	0.8366
Cholesterol in Medium VLDL	0.0212	0.0003	1.5789	0.0797	0.1063	0.0067	-0.0053	0.0187	0.2732	0.7529
Cholesteryl Esters in Medium VLDL	0.0248	1.93E-05	31.8499	0.3113	0.3591	0.0047	-0.0023	0.0117	0.1877	0.6370
Free Cholesterol in Medium VLDL	3.11E-06	1.81E-10	0.0536	0.0108	0.0195	0.0020	-0.0035	0.0075	0.4703	0.7859

Triglycerides in Medium VLDL	0.0017	0.0001	0.0248	3.12E-06	1.72E-05	-0.0083	-0.0321	0.0156	0.4974	0.7939
Concentration of Small VLDL Particles	NA	NA	NA	NA	0.0222	3.01E-07	-2.22E-06	2.82E-06	0.8149	0.9182
Total Lipids in Small VLDL	0.0634	0.0080	0.5002	0.0089	0.0164	0.0029	-0.0228	0.0285	0.8267	0.9230
Phospholipids in Small VLDL	6.75E-06	7.67E-10	0.0595	0.0102	0.0187	0.0018	-0.0039	0.0075	0.5361	0.8109
Cholesterol in Small VLDL	0.0099	0.0001	1.9328	0.0864	0.1127	0.0044	-0.0052	0.0140	0.3686	0.7818
Cholesteryl Esters in Small VLDL	0.0035	1.03E-06	11.8920	0.1729	0.2071	0.0028	-0.0033	0.0089	0.3702	0.7818
Free Cholesterol in Small VLDL	3.18E-08	1.32E-14	0.0766	0.0213	0.0350	0.0016	-0.0019	0.0051	0.3735	0.7818
Triglycerides in Small VLDL	0.0017	1.96E-05	0.1423	0.0048	0.0092	-0.0033	-0.0161	0.0094	0.6078	0.8311
Concentration of Very Small VLDL Particles	NA	NA	NA	NA	0.1244	0.0000	0.0000	0.0000	0.1660	0.6370
Total Lipids in Very Small VLDL	9.5284	0.8281	1.10E+02	0.0705	0.0961	0.0105	-0.0068	0.0279	0.2343	0.7030
Phospholipids in Very Small VLDL	5.75E+03	2.3029	1.44E+07	0.0301	0.0464	0.0031	-0.0023	0.0084	0.2592	0.7420
Cholesterol in Very Small VLDL	2.06E+02	1.7962	2.36E+04	0.0277	0.0437	0.0071	-0.0019	0.0160	0.1237	0.6370
Cholesteryl Esters in Very Small VLDL	2.10E+03	2.2596	1.95E+06	0.0283	0.0441	0.0051	-0.0012	0.0113	0.1111	0.6370
Free Cholesterol in Very Small VLDL	1.08E+07	3.8714	3.00E+13	0.0325	0.0494	0.0020	-0.0009	0.0048	0.1743	0.6370
Triglycerides in Very Small VLDL	1.5313	0.0001	19982.5624	0.9298	0.9409	0.0004	-0.0043	0.0051	0.8642	0.9480
Concentration of IDL Particles	NA	NA	NA	NA	0.7524	0.0000	0.0000	0.0000	0.1011	0.6370
Total Lipids in IDL	0.8834	0.3333	2.3409	0.8030	0.8333	0.0340	-0.0149	0.0828	0.1732	0.6370
Phospholipids in IDL	2.5919	0.0473	142.0581	0.6411	0.6882	0.0074	-0.0041	0.0188	0.2094	0.6771
Cholesterol in IDL	0.6056	0.1517	2.4177	0.4776	0.5241	0.0250	-0.0104	0.0604	0.1664	0.6370
Cholesteryl Esters in IDL	0.3603	0.0536	2.4220	0.2937	0.3419	0.0174	-0.0087	0.0435	0.1908	0.6370

Free Cholesterol in IDL	1.7854	0.0130	244.8101	0.8174	0.8447	0.0076	-0.0020	0.0172	0.1212	0.6370
Triglycerides in IDL	72.9675	0.0589	90422.7361	0.2378	0.2795	0.0016	-0.0042	0.0074	0.5918	0.8311
Concentration of Large LDL Particles	NA	NA	NA	NA	0.0560	1.56E-05	-1.75E-05	0.0000	0.3546	0.7818
Total Lipids in Large LDL	0.3178	0.1467	0.6886	0.0037	0.0074	0.0217	-0.0459	0.0893	0.5291	0.8082
Phospholipids in Large LDL	0.0061	0.0002	0.2057	0.0045	0.0089	0.0069	-0.0079	0.0217	0.3605	0.7818
Cholesterol in Large LDL	0.1806	0.0629	0.5185	0.0015	0.0035	0.0133	-0.0369	0.0635	0.6046	0.8311
Cholesteryl Esters in Large LDL	0.0809	0.0196	0.3336	0.0005	0.0015	0.0072	-0.0302	0.0445	0.7061	0.8606
Free Cholesterol in Large LDL	0.0118	0.0002	0.6018	0.0269	0.0427	0.0061	-0.0073	0.0194	0.3715	0.7818
Triglycerides in Large LDL	12.1979	0.0069	2.16E+04	0.5123	0.5572	0.0015	-0.0043	0.0073	0.6054	0.8311
Concentration of Medium LDL Particles	NA	NA	NA	NA	0.0079	5.60E-06	-8.82E-06	2.00E-05	0.4463	0.7859
Total Lipids in Medium LDL	0.0232	0.0042	0.1271	1.44E-05	0.0001	0.0066	-0.0248	0.0380	0.6784	0.8532
Phospholipids in Medium LDL	1.27E-07	1.48E-10	0.0001	4.13E-06	2.14E-05	0.0018	-0.0062	0.0097	0.6636	0.8532
Cholesterol in Medium LDL	0.0042	0.0004	0.0475	9.44E-06	4.42E-05	0.0047	-0.0175	0.0269	0.6752	0.8532
Cholesteryl Esters in Medium LDL	0.0006	2.52E-05	0.0162	8.26E-06	3.94E-05	0.0026	-0.0141	0.0193	0.7616	0.8820
Free Cholesterol in Medium LDL	1.36E-08	1.89E-12	0.0001	0.0001	0.0003	0.0022	-0.0038	0.0081	0.4780	0.7882
Triglycerides in Medium LDL	0.0004	4.62E-13	301243.5044	0.4506	0.5005	0.0001	-0.0022	0.0025	0.9087	0.9597
Concentration of Small LDL Particles	NA	NA	NA	NA	0.0393	3.04E-06	-4.08E-06	1.02E-05	0.4024	0.7859
Total Lipids in Small LDL	0.0004	5.00E-06	0.0251	0.0003	0.0009	0.0043	-0.0083	0.0168	0.5073	0.7995
Phospholipids in Small LDL	1.11E-10	6.99E-17	0.0002	0.0016	0.0039	0.0016	-0.0020	0.0053	0.3764	0.7818
Cholesterol in Small LDL	3.10E-06	4.99E-09	0.0019	0.0001	0.0004	0.0028	-0.0056	0.0112	0.5144	0.8006

Cholesteryl Esters in Small LDL	5.22E-08	9.57E-12	0.0003	0.0001	0.0005	0.0016	-0.0047	0.0079	0.6195	0.8366
Free Cholesterol in Small LDL	1.41E-16	1.31E-25	1.52E-07	0.0006	0.0016	0.0012	-0.0012	0.0036	0.3295	0.7818
Triglycerides in Small LDL	1.84E-16	1.12E-34	302.9650	0.0905	0.1168	-0.0002	-0.0014	0.0011	0.7754	0.8939
Concentration of Very Large HDL Particles	NA	NA	NA	NA	1.05E-11	9.05E-06	-2.75E-06	2.09E-05	0.1326	0.6370
Total Lipids in Very Large HDL	7.19E+05	2.20E+04	2.35E+07	3.51E-14	7.26E-13	0.0075	-0.0023	0.0173	0.1328	0.6370
Phospholipids in Very Large HDL	1.12E+11	1.98E+08	6.29E+13	3.57E-15	8.85E-14	0.0040	-0.0015	0.0095	0.1584	0.6370
Cholesterol in Very Large HDL	4.10E+12	1.32E+09	1.27E+16	1.47E-12	2.15E-11	0.0036	-0.0007	0.0079	0.1035	0.6370
Cholesteryl Esters in Very Large HDL	1.33E+17	1.76E+12	1.01E+22	6.00E-12	8.27E-11	0.0027	-0.0005	0.0058	0.0963	0.6370
Free Cholesterol in Very Large HDL	3.66E+46	2.03E+34	6.61E+58	9.60E-14	1.83E-12	0.0009	-0.0003	0.0020	0.1324	0.6370
Triglycerides in Very Large HDL	1.92E+25	0.0066	5.58E+52	0.0712	0.0964	-2.68E-06	-0.0006	0.0006	0.9930	0.9930
Concentration of Large HDL Particles	NA	NA	NA	NA	1.80E-15	3.77E-05	0.0000	0.0001	0.1620	0.6370
Total Lipids in Large HDL	2.38E+03	3.99E+02	1.42E+04	1.34E-17	8.33E-16	0.0135	-0.0081	0.0351	0.2214	0.6979
Phospholipids in Large HDL	1.00E+08	1.30E+06	7.79E+09	1.04E-16	4.29E-15	0.0043	-0.0054	0.0140	0.3812	0.7844
Cholesterol in Large HDL	3.07E+05	1.32E+04	7.12E+06	3.49E-15	8.85E-14	0.0097	-0.0027	0.0221	0.1253	0.6370
Cholesteryl Esters in Large HDL	1.10E+07	1.69E+05	7.09E+08	2.59E-14	5.83E-13	0.0077	-0.0020	0.0173	0.1192	0.6370
Free Cholesterol in Large HDL	6.29E+22	1.87E+17	2.11E+28	6.22E-16	1.93E-14	0.0020	-0.0009	0.0050	0.1771	0.6370
Triglycerides in Large HDL	7.64E+10	2.14E+04	2.73E+17	0.0011	0.0029	-0.0005	-0.0029	0.0019	0.6740	0.8532
Concentration of Medium HDL Particles	NA	NA	NA	NA	0.0006	-0.0001	-0.0001	0.0000	0.1147	0.6370
Total Lipids in Medium HDL	0.0134	0.0013	0.1399	0.0003	0.0010	-0.0174	-0.0384	0.0037	0.1053	0.6370

Phospholipids in Medium HDL	0.0005	6.26E-06	0.0473	0.0010	0.0025	-0.0093	-0.0206	0.0021	0.1085	0.6370
Cholesterol in Medium HDL	2.00E-07	7.09E-10	0.0001	8.30E-08	6.05E-07	-0.0062	-0.0134	0.0009	0.0877	0.6370
Cholesteryl Esters in Medium HDL	1.06E-08	1.62E-11	6.85E-06	2.72E-08	2.04E-07	-0.0053	-0.0114	0.0007	0.0840	0.6370
Free Cholesterol in Medium HDL	1.05E-20	6.95E-35	1.59E-06	0.0058	0.0107	-0.0009	-0.0025	0.0006	0.2422	0.7095
Triglycerides in Medium HDL	0.2161	3.47E-06	13464.0626	0.7856	0.8186	-0.0019	-0.0059	0.0022	0.3723	0.7818
Concentration of Small HDL Particles	NA	NA	NA	NA	5.62E-15	-0.0002	-0.0004	0.0001	0.1868	0.6370
Total Lipids in Small HDL	4.52E-03	0.0010	0.0200	1.18E-12	1.82E-11	-0.0233	-0.0524	0.0058	0.1160	0.6370
Phospholipids in Small HDL	0.0002	1.24E-05	0.0026	2.30E-10	2.48E-09	-0.0148	-0.0318	0.0022	0.0871	0.6370
Cholesterol in Small HDL	9.00E-08	2.19E-09	3.70E-06	1.20E-17	8.33E-16	-0.0070	-0.0176	0.0035	0.1915	0.6370
Cholesteryl Esters in Small HDL	2.42E-09	2.77E-11	2.11E-07	3.25E-18	4.03E-16	-0.0056	-0.0140	0.0028	0.1919	0.6370
Free Cholesterol in Small HDL	2.60E-24	2.99E-32	2.27E-16	5.84E-09	5.17E-08	-0.0014	-0.0042	0.0013	0.3088	0.7818
Triglycerides in Small HDL	3.90E-07	1.82E-13	0.8400	0.0473	0.0669	-0.0015	-0.0050	0.0021	0.4201	0.7859
Phospholipids to Total Lipids in Chylomicrons and Extremely Large VLDL percentage	1.0545	0.9832	1.1309	0.1371	0.1700	-0.0850	-0.7863	0.6162	0.8121	0.9182
Cholesterol to Total Lipids in Chylomicrons and Extremely Large VLDL percentage	1.0080	0.9873	1.0291	0.4521	0.5005	1.4288	-0.8375	3.6950	0.2164	0.6909

Cholesteryl Esters to Total Lipids in Chylomicrons and Extremely Large VLDL percentage	1.0116	0.9842	1.0397	0.4114	0.4617	0.6834	-1.0025	2.3693	0.4267	0.7859
Free Cholesterol to Total Lipids in Chylomicrons and Extremely Large VLDL percentage	1.0126	0.9560	1.0725	0.6695	0.7157	0.7453	-0.0370	1.5277	0.0619	0.6065
Triglycerides to Total Lipids in Chylomicrons and Extremely Large VLDL percentage	0.9906	0.9732	1.0083	0.2971	0.3443	-1.343 7	-3.9197	1.2323	0.3064	0.7818
Phospholipids to Total Lipids in Very Large VLDL percentage	1.4861	1.3022	1.6960	4.14E-09	3.80E-08	0.1941	-0.2737	0.6619	0.4159	0.7859
Cholesterol to Total Lipids in Very Large VLDL percentage	1.0656	1.0320	1.1002	0.0001	0.0004	1.2754	0.0735	2.4774	0.0376	0.5066
Cholesteryl Esters to Total Lipids in Very Large VLDL percentage	1.0671	1.0231	1.1130	0.0025	0.0057	0.9459	-0.0098	1.9016	0.0524	0.5672
Free Cholesterol to Total Lipids in Very Large VLDL percentage	1.4065	1.2528	1.5790	7.53E-09	6.23E-08	0.3295	0.0236	0.6354	0.0347	0.5066
Triglycerides to Total Lipids in Very Large VLDL percentage	0.9335	0.9083	0.9595	8.99E-07	5.44E-06	-1.469 6	-2.8624	-0.0768	0.0387	0.5066
Phospholipids to Total Lipids in Large VLDL percentage	1.2015	1.0798	1.3368	0.0008	0.0020	-0.033 5	-0.5354	0.4684	0.8958	0.9574

Cholesterol to Total Lipids in Large VLDL percentage	1.1362	1.0904	1.1840	1.20E-09	1.18E-08	0.8795	0.0239	1.7351	0.0439	0.5392
Cholesteryl Esters to Total Lipids in Large VLDL percentage	1.1902	1.1261	1.2579	6.94E-10	7.17E-09	0.6956	0.0528	1.3385	0.0339	0.5066
Free Cholesterol to Total Lipids in Large VLDL percentage	1.4784	1.2661	1.7263	7.70E-07	4.90E-06	0.1838	-0.0733	0.4409	0.1611	0.6370
Triglycerides to Total Lipids in Large VLDL percentage	0.9085	0.8808	0.9371	1.23E-09	1.18E-08	-0.8460	-1.9895	0.2975	0.1469	0.6370
Phospholipids to Total Lipids in Medium VLDL percentage	1.3660	1.1913	1.5664	7.96E-06	3.94E-05	0.5058	0.1559	0.8556	0.0046	0.3294
Cholesterol to Total Lipids in Medium VLDL percentage	1.0714	1.0287	1.1159	0.0009	0.0024	1.6176	0.4107	2.8246	0.0086	0.3589
Cholesteryl Esters to Total Lipids in Medium VLDL percentage	1.0892	1.0310	1.1508	0.0023	0.0053	1.1614	0.2685	2.0543	0.0108	0.3851
Free Cholesterol to Total Lipids in Medium VLDL percentage	1.3575	1.1683	1.5773	0.0001	0.0003	0.4563	0.1308	0.7817	0.0060	0.3294
Triglycerides to Total Lipids in Medium VLDL percentage	0.9427	0.9130	0.9732	0.0003	0.0009	-2.1234	-3.6551	-0.5917	0.0066	0.3294
Phospholipids to Total Lipids in Small VLDL percentage	0.9790	0.8583	1.1167	0.7521	0.7903	0.4089	0.0512	0.7665	0.0251	0.4784
Cholesterol to Total Lipids in Small VLDL percentage	1.0499	0.9946	1.1082	0.0780	0.1045	1.0407	0.1532	1.9283	0.0216	0.4784

Cholesteryl Esters to Total Lipids in Small VLDL percentage	1.1445	1.0459	1.2524	0.0033	0.0069	0.6093	0.0698	1.1488	0.0269	0.4784
Free Cholesterol to Total Lipids in Small VLDL percentage	0.9947	0.8792	1.1255	0.9333	0.9409	0.4315	0.0511	0.8120	0.0262	0.4784
Triglycerides to Total Lipids in Small VLDL percentage	0.9769	0.9397	1.0154	0.2360	0.2787	-1.4496	-2.6798	-0.2195	0.0209	0.4784
Phospholipids to Total Lipids in Very Small VLDL percentage	1.6540	1.1994	2.2810	0.0022	0.0050	-0.0712	-0.2134	0.0711	0.3268	0.7818
Cholesterol to Total Lipids in Very Small VLDL percentage	1.0480	0.9884	1.1113	0.1166	0.1468	0.7955	-0.0442	1.6352	0.0633	0.6065
Cholesteryl Esters to Total Lipids in Very Small VLDL percentage	1.0523	0.9839	1.1255	0.1369	0.1700	0.6966	-0.0346	1.4278	0.0618	0.6065
Free Cholesterol to Total Lipids in Very Small VLDL percentage	1.3903	0.9550	2.0240	0.0855	0.1125	0.0989	-0.0326	0.2304	0.1404	0.6370
Triglycerides to Total Lipids in Very Small VLDL percentage	0.9293	0.8710	0.9916	0.0268	0.0427	-0.7244	-1.5046	0.0559	0.0688	0.6118
Phospholipids to Total Lipids in IDL percentage	1.8992	1.4149	2.5493	1.95E-05	0.0001	-0.0766	-0.2412	0.0880	0.3615	0.7818
Cholesterol to Total Lipids in IDL percentage	0.9286	0.8647	0.9971	0.0413	0.0607	0.2748	-0.3134	0.8631	0.3596	0.7818
Cholesteryl Esters to Total Lipids in IDL percentage	0.8490	0.7749	0.9302	0.0004	0.0014	0.1222	-0.3386	0.5830	0.6032	0.8311

Free Cholesterol to Total Lipids in IDL percentage	1.1761	0.9631	1.4361	0.1116	0.1419	0.1527	-0.0840	0.3893	0.2060	0.6748
Triglycerides to Total Lipids in IDL percentage	1.0422	0.9606	1.1307	0.3204	0.3678	-0.198 3	-0.7166	0.3201	0.4533	0.7859
Phospholipids to Total Lipids in Large LDL percentage	1.1478	0.8616	1.5291	0.3461	0.3938	0.1239	-0.0264	0.2741	0.1060	0.6370
Cholesterol to Total Lipids in Large LDL percentage	0.8761	0.8195	0.9366	0.0001	0.0004	-0.112 6	-0.4766	0.2514	0.5442	0.8145
Cholesteryl Esters to Total Lipids in Large LDL percentage	0.7698	0.7112	0.8333	9.51E-11	1.18E-09	-0.292 5	-0.5652	-0.0199	0.0355	0.5066
Free Cholesterol to Total Lipids in Large LDL percentage	1.2009	0.9817	1.4690	0.0750	0.1011	0.1800	-0.0784	0.4384	0.1721	0.6370
Triglycerides to Total Lipids in Large LDL percentage	1.1456	1.0596	1.2386	0.0006	0.0018	-0.011 3	-0.4089	0.3864	0.9558	0.9714
Phospholipids to Total Lipids in Medium LDL percentage	0.7695	0.5592	1.0588	0.1076	0.1375	0.0288	-0.1188	0.1763	0.7020	0.8606
Cholesterol to Total Lipids in Medium LDL percentage	0.8384	0.7601	0.9247	0.0004	0.0013	0.0726	-0.2655	0.4106	0.6738	0.8532
Cholesteryl Esters to Total Lipids in Medium LDL percentage	0.7725	0.6898	0.8653	0.0000	0.0000	-0.141 0	-0.4930	0.2111	0.4324	0.7859
Free Cholesterol to Total Lipids in Medium LDL percentage	1.0368	0.9137	1.1766	0.5752	0.6229	0.2134	-0.1625	0.5893	0.2656	0.7515
Triglycerides to Total Lipids in Medium LDL percentage	1.2330	1.1185	1.3592	2.52E-05	0.0001	-0.101 3	-0.4375	0.2349	0.5545	0.8145

Phospholipids to Total Lipids in Small LDL percentage	1.2491	1.0953	1.4245	0.0009	0.0024	0.1441	-0.1894	0.4775	0.3969	0.7844
Cholesterol to Total Lipids in Small LDL percentage	0.8361	0.7586	0.9216	0.0003	0.0010	0.0830	-0.3079	0.4740	0.6771	0.8532
Cholesteryl Esters to Total Lipids in Small LDL percentage	0.8357	0.7433	0.9397	0.0027	0.0060	-0.1513	-0.5450	0.2425	0.4512	0.7859
Free Cholesterol to Total Lipids in Small LDL percentage	0.9657	0.8744	1.0666	0.4916	0.5371	0.2343	-0.1867	0.6553	0.2751	0.7529
Triglycerides to Total Lipids in Small LDL percentage	1.0518	0.9447	1.1711	0.3569	0.4023	-0.2270	-0.6044	0.1503	0.2382	0.7060
Phospholipids to Total Lipids in Very Large HDL percentage	1.2088	1.1208	1.3037	0.0000	5.43E-06	0.9033	-0.4050	2.2116	0.1758	0.6370
Cholesterol to Total Lipids in Very Large HDL percentage	0.9465	0.9152	0.9789	0.0014	0.0034	-0.4034	-1.4946	0.6879	0.4686	0.7859
Cholesteryl Esters to Total Lipids in Very Large HDL percentage	0.9528	0.9098	0.9978	0.0399	0.0592	-0.0568	-0.8283	0.7147	0.8851	0.9574
Free Cholesterol to Total Lipids in Very Large HDL percentage	0.8552	0.7672	0.9533	0.0048	0.0092	-0.3465	-0.9132	0.2202	0.2306	0.7030
Triglycerides to Total Lipids in Very Large HDL percentage	0.8653	0.7824	0.9571	0.0049	0.0093	-0.5000	-1.6022	0.6023	0.3738	0.7818
Phospholipids to Total Lipids in Large HDL percentage	0.8578	0.8144	0.9035	6.82E-09	5.84E-08	-0.9285	-1.5542	-0.3029	0.0037	0.3294

Cholesterol to Total Lipids in Large HDL percentage	1.0538	1.0000	1.1105	0.0500	0.0692	0.4078	-0.6182	1.4338	0.4357	0.7859
Cholesteryl Esters to Total Lipids in Large HDL percentage	1.0420	0.9862	1.1008	0.1427	0.1752	0.3207	-0.5807	1.2222	0.4854	0.7900
Free Cholesterol to Total Lipids in Large HDL percentage	1.4696	1.1389	1.8964	0.0031	0.0067	0.0871	-0.1474	0.3215	0.4665	0.7859
Triglycerides to Total Lipids in Large HDL percentage	1.0452	0.9897	1.1037	0.1125	0.1423	0.5207	-0.1487	1.1901	0.1273	0.6370
Phospholipids to Total Lipids in Medium HDL percentage	0.8600	0.8091	0.9141	1.26E-06	7.41E-06	-0.4988	-0.7874	-0.2101	0.0007	0.1786
Cholesterol to Total Lipids in Medium HDL percentage	0.8988	0.8268	0.9771	0.0123	0.0215	-0.1207	-0.6117	0.3702	0.6296	0.8366
Cholesteryl Esters to Total Lipids in Medium HDL percentage	0.8954	0.8220	0.9753	0.0113	0.0201	-0.1424	-0.6294	0.3446	0.5665	0.8181
Free Cholesterol to Total Lipids in Medium HDL percentage	1.4333	0.6713	3.0605	0.3523	0.3989	0.0216	-0.0443	0.0875	0.5209	0.8057
Triglycerides to Total Lipids in Medium HDL percentage	1.0913	1.0546	1.1293	5.57E-07	3.64E-06	0.6195	0.1186	1.1205	0.0154	0.4563
Phospholipids to Total Lipids in Small HDL percentage	1.6958	1.3577	2.1181	3.24E-06	1.75E-05	-0.1499	-0.3692	0.0694	0.1801	0.6370
Cholesterol to Total Lipids in Small HDL percentage	0.7693	0.6986	0.8471	9.52E-08	6.74E-07	0.0200	-0.3700	0.4100	0.9199	0.9597

Cholesteryl Esters to Total Lipids in Small HDL percentage	0.7663	0.7066	0.8311	1.29E-10	1.52E-09	-0.0737	-0.4869	0.3396	0.7267	0.8742
Free Cholesterol to Total Lipids in Small HDL percentage	2.2516	1.6997	2.9827	1.54E-08	1.19E-07	0.0937	-0.0066	0.1940	0.0671	0.6118
Triglycerides to Total Lipids in Small HDL percentage	1.2722	1.1452	1.4134	7.30E-06	3.69E-05	0.1299	-0.1222	0.3821	0.3123	0.7818

[a]Models were adjusted for age, sex, ethnicity, Townsend deprivation index, smoking status, alcohol consumption, physical activity, education, waist circumference, hypertension status, diabetes status, ALT， HDL, and myopia status.

[b]Models were adjusted for age, sex, ethnicity, Townsend deprivation index, smoking status, alcohol consumption, physical activity, education, waist circumference, hypertension status, diabetes status, ALT and HDL.

HR Hazards ratio, CI Confidence interval, ALT Alanine aminotransferase, LDL Low-density lipoprotein, VLDL Very low-density lipoprotein, IDL Intermediate-density lipoprotein, HDL High-density lipoprotein, HDL-C High-density lipoprotein cholesterol.

NA denotes statistical models with convergence failure or sparse data.

Table S14. Selection of metabolites as potential mediators between high myopia and incident viral hepatitis

Metabolites	HR[a]	CI_lower	CI_upper	P	FDR	Beta[b]	CI_lower	CI_upper	P	FDR
Total Cholesterol	0.5741	0.2517	1.3093	0.1871	0.4902	0.0607	-0.0928	0.2143	0.4380	0.7859
Total Cholesterol Minus HDL-C	0.5740	0.2517	1.3093	0.1870	0.4902	0.0607	-0.0928	0.2143	0.4380	0.7859
Remnant Cholesterol (Non-HDL, Non-LDL -Cholesterol)	0.4192	0.0804	2.1867	0.3023	0.5046	0.0399	-0.0364	0.1163	0.3052	0.7818
VLDL Cholesterol	0.1847	0.0102	3.3415	0.2529	0.4973	0.0149	-0.0313	0.0611	0.5262	0.8082
Clinical LDL Cholesterol	0.4999	0.1921	1.3007	0.1553	0.4755	0.0625	-0.0702	0.1951	0.3558	0.7818
LDL Cholesterol	0.2842	0.0729	1.1071	0.0698	0.3605	0.0208	-0.0593	0.1009	0.6108	0.8311
HDL Cholesterol	NA	NA	NA	NA	NA	3.61E-17	-2.29E-17	9.51E-17	0.2306	0.7030
Total Triglycerides	0.4657	0.1165	1.8608	0.2796	0.5045	-0.0415	-0.1638	0.0808	0.5062	0.7995
Triglycerides in VLDL	0.3276	0.0543	1.9756	0.2235	0.4923	-0.0407	-0.1414	0.0600	0.4283	0.7859
Triglycerides in LDL	0.0140	1.52E-08	1.28E+04	0.5421	0.7267	0.0015	-0.0078	0.0107	0.7550	0.8786
Triglycerides in HDL	0.1273	4.13E-07	3.92E+04	0.7492	0.8602	-0.0038	-0.0142	0.0065	0.4651	0.7859
Total Phospholipids in Lipoprotein Particles	0.2387	0.0359	1.5870	0.1383	0.4710	0.0033	-0.0715	0.0781	0.9318	0.9628
Phospholipids in VLDL	0.0730	0.0011	4.9688	0.2242	0.4923	0.0014	-0.0338	0.0367	0.9375	0.9646
Phospholipids in LDL	0.0199	0.0003	1.2679	0.0646	0.3409	0.0103	-0.0157	0.0363	0.4360	0.7859
Phospholipids in HDL	0.0823	0.0002	37.5364	0.4241	0.6114	-0.0158	-0.0385	0.0069	0.1713	0.6370
Total Esterified Cholesterol	0.4345	0.1325	1.4248	0.1689	0.4881	0.0401	-0.0669	0.1471	0.4624	0.7859
Cholesteryl Esters in VLDL	0.0785	0.0006	9.9653	0.3032	0.5046	0.0121	-0.0144	0.0386	0.3720	0.7818

Cholesteryl Esters in HDL	6.80E-07	5.51E-18	8.39E+04	0.2758	0.5045	-0.0006	-0.0041	0.0029	0.7468	0.8786
Total Free Cholesterol	0.2016	0.0140	2.9111	0.2398	0.4923	0.0206	-0.0264	0.0676	0.3897	0.7844
Free Cholesterol in VLDL	0.0109	9.32E-06	12.6562	0.2093	0.4902	0.0029	-0.0176	0.0233	0.7847	0.8963
Free Cholesterol in LDL	0.0261	0.0001	8.7341	0.2190	0.4923	0.0094	-0.0121	0.0310	0.3910	0.7844
Free Cholesterol in HDL	1.45E+06	1.16E-05	1.79E+17	0.2765	0.5045	0.0006	-0.0029	0.0041	0.7477	0.8786
Total Lipids in Lipoprotein Particles	0.7148	0.4529	1.1281	0.1492	0.4755	0.0225	-0.2875	0.3326	0.8866	0.9574
Total Lipids in VLDL	0.5400	0.2094	1.3925	0.2023	0.4902	-0.0243	-0.1963	0.1477	0.7814	0.8963
Total Lipids in LDL	0.4063	0.1509	1.0939	0.0747	0.3713	0.0326	-0.0781	0.1432	0.5636	0.8181
Total Lipids in HDL	0.2325	0.0032	17.0539	0.5056	0.6890	-0.0197	-0.0520	0.0127	0.2329	0.7030
Total Concentration of Lipoprotein Particles	NA	NA	NA	NA	NA	-0.0001	-0.0004	0.0001	0.3390	0.7818
Concentration of VLDL Particles	NA	NA	NA	NA	NA	2.08E-06	-6.61E-06	1.08E-05	0.6392	0.8421
Concentration of LDL Particles	NA	NA	NA	NA	NA	2.43E-05	-2.91E-05	0.0001	0.3730	0.7818
Concentration of HDL Particles	NA	NA	NA	NA	NA	-0.0002	-0.0004	0.0001	0.1531	0.6370
Average Diameter for VLDL Particles	0.5455	0.3220	0.9240	0.0242	0.1876	-0.2104	-0.4186	-0.0022	0.0476	0.5392
Average Diameter for LDL Particles	0.8683	0.0002	4.43E+03	0.9741	0.9821	0.0060	-0.0099	0.0219	0.4597	0.7859
Average Diameter for HDL Particles	2.45E+03	23.8492	2.51E+05	0.0010	0.0142	0.0163	-0.0064	0.0390	0.1589	0.6370

Triglycerides to Phosphoglycerides ratio	0.4609	0.0134	15.9056	0.6682	0.8148	-0.0201	-0.0560	0.0158	0.2720	0.7529
Total Cholines	0.1071	0.0088	1.2983	0.0793	0.3781	-0.0041	-0.0625	0.0543	0.8908	0.9574
Phosphatidylcholines	0.1179	0.0081	1.7230	0.1182	0.4561	-0.0088	-0.0637	0.0461	0.7533	0.8786
Sphingomyelins	0.0009	2.26E-09	3.55E+02	0.2859	0.5045	0.0066	-0.0032	0.0164	0.1875	0.6370
Apolipoprotein B	0.1436	0.0054	3.8014	0.2456	0.4923	0.0194	-0.0181	0.0569	0.3106	0.7818
Apolipoprotein A1	0.0001	2.39E-08	0.7943	0.0442	0.2829	-0.0142	-0.0311	0.0027	0.1000	0.6370
Apolipoprotein B to Apolipoprotein A1 ratio	0.2435	0.0044	13.6090	0.4913	0.6732	0.0360	0.0066	0.0654	0.0165	0.4563
Total Fatty Acids	0.7866	0.5673	1.0907	0.1501	0.4755	-0.0883	-0.5916	0.4149	0.7307	0.8747
Degree of Unsaturation	0.0077	8.12E-07	73.7042	0.2983	0.5046	0.0046	-0.0100	0.0191	0.5374	0.8109
Omega-3 Fatty Acids	0.0221	0.0005	0.9284	0.0456	0.2829	0.0014	-0.0416	0.0444	0.9480	0.9691
Omega-6 Fatty Acids	0.4184	0.1277	1.3704	0.1500	0.4755	-0.0039	-0.1238	0.1160	0.9497	0.9691
Polyunsaturated Fatty Acids	0.4021	0.1695	0.9539	0.0387	0.2669	-0.0024	-0.1450	0.1401	0.9733	0.9812
Monounsaturated Fatty Acids	0.5329	0.2062	1.3774	0.1939	0.4902	-0.0289	-0.2109	0.1530	0.7551	0.8786
Saturated Fatty Acids	0.6219	0.2823	1.3702	0.2386	0.4923	-0.0570	-0.2638	0.1499	0.5893	0.8311
Linoleic Acid	0.5190	0.1672	1.6109	0.2564	0.4973	0.0059	-0.1171	0.1290	0.9250	0.9597
Docosahexaenoic Acid	0.0001	9.43E-10	11.4027	0.1214	0.4561	0.0020	-0.0134	0.0175	0.7985	0.9079
Omega-3 Fatty Acids to Total Fatty Acids percentage	0.6236	0.3592	1.0826	0.0933	0.4061	0.0167	-0.2773	0.3107	0.9112	0.9597

Polyunsaturated Fatty Acids to Total Fatty Acids percentage	1.0246	0.8579	1.2238	0.7884	0.8668	0.3037	-0.4158	1.0231	0.4078	0.7859
Monounsaturated Fatty Acids to Total Fatty Acids percentage	0.9286	0.7128	1.2097	0.5829	0.7560	-0.1320	-0.6069	0.3429	0.5857	0.8311
Saturated Fatty Acids to Total Fatty Acids percentage	1.0356	0.7388	1.4516	0.8393	0.9010	-0.1717	-0.5526	0.2092	0.3768	0.7818
Linoleic Acid to Total Fatty Acids percentage	1.0455	0.8519	1.2831	0.6702	0.8148	0.3193	-0.2947	0.9334	0.3079	0.7818
Docosahexaenoic Acid to Total Fatty Acids percentage	0.5323	0.1545	1.8345	0.3179	0.5087	0.0204	-0.1073	0.1481	0.7541	0.8786
Polyunsaturated Fatty Acids to Monounsaturated Fatty Acids ratio	1.2729	0.1599	10.1315	0.8196	0.8876	0.0256	-0.0328	0.0839	0.3902	0.7844
Omega-6 Fatty Acids to Omega-3 Fatty Acids ratio	1.0936	1.0055	1.1894	0.0368	0.2604	0.3354	-0.5167	1.1875	0.4402	0.7859
Alanine	0.0017	8.41E-08	34.9214	0.2084	0.4902	-0.0152	-0.0302	-0.0002	0.0466	0.5392
Glutamine	0.0255	1.03E-05	63.2722	0.3576	0.5441	0.0036	-0.0127	0.0200	0.6619	0.8532
Glycine	1.3488	1.81E-05	1.00E+05	0.9583	0.9821	-0.0011	-0.0108	0.0086	0.8210	0.9209
Histidine	0.0000	2.76E-48	1.59E+12	0.2462	0.4923	-0.0005	-0.0024	0.0014	0.6283	0.8366

Total Concentration of Branched-Chain Amino Acids (Leucine + Isoleucine + Valine)	18.2130	0.0065	5.09E+04	0.4735	0.6598	-0.0044	-0.0191	0.0103	0.5561	0.8145
Isoleucine	1.28E+11	0.0009	1.85E+25	0.1241	0.4594	0.0002	-0.0028	0.0033	0.8928	0.9574
Leucine	1.84E+04	3.98E-07	8.53E+14	0.4332	0.6210	-0.0011	-0.0059	0.0038	0.6599	0.8532
Valine	9.0012	8.29E-07	9.78E+07	0.7904	0.8668	-0.0035	-0.0111	0.0040	0.3558	0.7818
Phenylalanine	3.26E+03	4.87E-15	2.18E+21	0.6993	0.8327	-0.0015	-0.0038	0.0007	0.1883	0.6370
Tyrosine	1.64E+13	0.0003	8.46E+29	0.1212	0.4561	0.0003	-0.0025	0.0031	0.8454	0.9398
Glucose	0.3504	0.1626	0.7553	0.0074	0.0710	0.1021	-0.1151	0.3194	0.3565	0.7818
Lactate	0.8774	0.4952	1.5547	0.6541	0.8031	-0.1872	-0.4267	0.0523	0.1255	0.6370
Pyruvate	1.06E-09	3.53E-22	3.20E+03	0.1587	0.4800	-0.0004	-0.0070	0.0063	0.9159	0.9597
Citrate	1.34E+04	5.56E-18	3.24E+25	0.7052	0.8327	0.0005	-0.0020	0.0030	0.6991	0.8606
3-Hydroxybutyrate	0.0553	1.25E-08	2.45E+05	0.7109	0.8355	0.0071	-0.0051	0.0193	0.2529	0.7322
Acetate	3.27E-07	2.61E-50	4.09E+36	0.7680	0.8658	-0.0007	-0.0031	0.0017	0.5531	0.8145
Acetoacetate	4.18E-24	8.74E-67	2.00E+19	0.2830	0.5045	0.0011	-0.0011	0.0032	0.3221	0.7818
Acetone	4.37E+07	2.12E-47	9.03E+61	0.7828	0.8668	0.0001	-0.0010	0.0012	0.9169	0.9597
Creatinine	0.0021	3.76E-28	1.17E+22	0.8320	0.8971	-0.0016	-0.0039	0.0006	0.1604	0.6370
Albumin	0.7788	0.6416	0.9454	0.0115	0.1056	-0.4367	-1.0891	0.2157	0.1894	0.6370
Glycoprotein Acetyls	0.0171	1.58E-05	18.4674	0.2535	0.4973	0.0011	-0.0207	0.0229	0.9234	0.9597

Concentration of Chylomicrons and Extremely Large VLDL Particles	NA	NA	NA	NA	NA	-1.11E-07	-4.21E-07	1.99E-07	0.4815	0.7888
Total Lipids in Chylomicrons and Extremely Large VLDL	0.1603	0.0050	5.0996	0.2997	0.5046	-0.0164	-0.0586	0.0258	0.4451	0.7859
Phospholipids in Chylomicrons and Extremely Large VLDL	2.84E-06	4.61E-16	1.74E+04	0.2667	0.5045	-0.0022	-0.0086	0.0042	0.4942	0.7939
Cholesterol in Chylomicrons and Extremely Large VLDL	0.0001	1.59E-13	1.68E+04	0.3237	0.5113	-0.0021	-0.0104	0.0063	0.6250	0.8366
Cholesteryl Esters in Chylomicrons and Extremely Large VLDL	4.18E-08	3.18E-23	5.51E+07	0.3388	0.5285	-0.0009	-0.0055	0.0036	0.6932	0.8606
Free Cholesterol in Chylomicrons and Extremely Large VLDL	1.56E-10	5.02E-27	4.82E+06	0.2437	0.4923	-0.0012	-0.0050	0.0027	0.5531	0.8145
Triglycerides in Chylomicrons and Extremely Large VLDL	0.0719	0.0004	14.2148	0.3291	0.5166	-0.0121	-0.0400	0.0158	0.3938	0.7844
Concentration of Very Large VLDL Particles	NA	NA	NA	NA	NA	-1.51E-07	-6.02E-07	3.01E-07	0.5134	0.8006
Total Lipids in Very Large VLDL	0.0110	1.19E-05	10.2044	0.1958	0.4902	-0.0098	-0.0365	0.0170	0.4743	0.7873
Phospholipids in Very Large VLDL	6.79E-10	7.31E-25	6.30E+05	0.2299	0.4923	-0.0013	-0.0064	0.0037	0.6104	0.8311

Cholesterol in Very Large VLDL	1.69E-09	1.26E-22	2.27E+04	0.1903	0.4902	-0.0005	-0.0058	0.0048	0.8536	0.9444
Cholesteryl Esters in Very Large VLDL	9.85E-18	2.89E-42	3.35E+07	0.1742	0.4881	3.82E-05	-0.0026	0.0027	0.9772	0.9812
Free Cholesterol in Very Large VLDL	3.23E-17	5.92E-44	1.77E+10	0.2267	0.4923	-0.0005	-0.0033	0.0022	0.7032	0.8606
Triglycerides in Very Large VLDL	6.36E-04	8.03E-09	50.3959	0.2009	0.4902	-0.0080	-0.0248	0.0089	0.3546	0.7818
Concentration of Large VLDL Particles	NA	NA	NA	NA	NA	-3.17E-07	-1.41E-06	7.74E-07	0.5684	0.8181
Total Lipids in Large VLDL	0.0294	0.0002	4.8700	0.1761	0.4881	-0.0122	-0.0472	0.0228	0.4939	0.7939
Phospholipids in Large VLDL	1.34E-07	1.42E-17	1.26E+03	0.1768	0.4881	-0.0021	-0.0097	0.0055	0.5828	0.8311
Cholesterol in Large VLDL	2.05E-06	2.09E-14	2.00E+02	0.1629	0.4867	-0.0007	-0.0094	0.0081	0.8811	0.9574
Cholesteryl Esters in Large VLDL	4.25E-11	1.39E-26	1.30E+05	0.1893	0.4902	0.0004	-0.0038	0.0046	0.8572	0.9444
Free Cholesterol in Large VLDL	1.86E-12	1.67E-28	2.07E+04	0.1519	0.4755	-0.0011	-0.0057	0.0036	0.6585	0.8532
Triglycerides in Large VLDL	0.0022	1.90E-07	26.1920	0.2016	0.4902	-0.0094	-0.0287	0.0099	0.3381	0.7818
Concentration of Medium VLDL Particles	NA	NA	NA	NA	NA	4.64E-07	-1.86E-06	2.79E-06	0.6952	0.8606
Total Lipids in Medium VLDL	0.0529	0.0018	1.5140	0.0859	0.3943	0.0007	-0.0394	0.0407	0.9744	0.9812
Phospholipids in Medium VLDL	2.80E-05	7.29E-12	1.08E+02	0.1754	0.4881	0.0022	-0.0068	0.0112	0.6316	0.8366
Cholesterol in Medium VLDL	0.0030	9.46E-08	98.2930	0.2741	0.5045	0.0067	-0.0053	0.0187	0.2732	0.7529
Cholesteryl Esters in Medium VLDL	0.0004	1.72E-10	807.7199	0.2889	0.5045	0.0047	-0.0023	0.0117	0.1877	0.6370

Free Cholesterol in Medium VLDL	1.84E-07	6.67E-18	5.07E+03	0.2061	0.4902	0.0020	-0.0035	0.0075	0.4703	0.7859
Triglycerides in Medium VLDL	0.0029	1.56E-06	5.5330	0.1297	0.4662	-0.0083	-0.0321	0.0156	0.4974	0.7939
Concentration of Small VLDL Particles	NA	NA	NA	NA	0.4923	3.01E-07	-2.22E-06	2.82E-06	0.8149	0.9182
Total Lipids in Small VLDL	0.0203	0.0001	5.8259	0.1771	0.4881	0.0029	-0.0228	0.0285	0.8267	0.9230
Phospholipids in Small VLDL	4.73E-08	5.74E-17	38.9232	0.1073	0.4426	0.0018	-0.0039	0.0075	0.5361	0.8109
Cholesterol in Small VLDL	2.01E-04	3.18E-10	126.6371	0.2115	0.4902	0.0044	-0.0052	0.0140	0.3686	0.7818
Cholesteryl Esters in Small VLDL	2.68E-06	1.92E-15	3.73E+03	0.2324	0.4923	0.0028	-0.0033	0.0089	0.3702	0.7818
Free Cholesterol in Small VLDL	2.39E-11	5.55E-25	1.03E+03	0.1268	0.4626	0.0016	-0.0019	0.0051	0.3735	0.7818
Triglycerides in Small VLDL	0.0007	2.09E-09	223.8312	0.2607	0.4973	-0.0033	-0.0161	0.0094	0.6078	0.8311
Concentration of Very Small VLDL Particles	NA	NA	NA	NA	NA	1.89E-06	-7.85E-07	4.57E-06	0.1660	0.6370
Total Lipids in Very Small VLDL	0.4111	0.0005	318.9397	0.7934	0.8668	0.0105	-0.0068	0.0279	0.2343	0.7030
Phospholipids in Very Small VLDL	0.2239	2.14E-10	2.34E+08	0.8877	0.9289	0.0031	-0.0023	0.0084	0.2592	0.7420
Cholesterol in Very Small VLDL	0.4917	8.27E-07	2.92E+05	0.9167	0.9472	0.0071	-0.0019	0.0160	0.1237	0.6370
Cholesteryl Esters in Very Small VLDL	0.4786	1.58E-09	1.45E+08	0.9410	0.9684	0.0051	-0.0012	0.0113	0.1111	0.6370
Free Cholesterol in Very Small VLDL	0.0383	1.95E-19	7.53E+15	0.8725	0.9168	0.0020	-0.0009	0.0048	0.1743	0.6370

Triglycerides in Very Small VLDL	0.0002	3.04E-16	2.02E+08	0.5529	0.7333	0.0004	-0.0043	0.0051	0.8642	0.9480
Concentration of IDL Particles	NA	NA	NA	NA	NA	1.15E-05	-2.25E-06	2.52E-05	0.1011	0.6370
Total Lipids in IDL	0.4092	0.0551	3.0412	0.3826	0.5648	0.0340	-0.0149	0.0828	0.1732	0.6370
Phospholipids in IDL	0.0547	1.61E-06	1.86E+03	0.5853	0.7560	0.0074	-0.0041	0.0188	0.2094	0.6771
Cholesterol in IDL	0.2686	0.0176	4.1068	0.3448	0.5312	0.0250	-0.0104	0.0604	0.1664	0.6370
Cholesteryl Esters in IDL	0.1387	0.0035	5.5567	0.2941	0.5046	0.0174	-0.0087	0.0435	0.1908	0.6370
Free Cholesterol in IDL	0.0343	9.42E-08	1.25E+04	0.6058	0.7612	0.0076	-0.0020	0.0172	0.1212	0.6370
Triglycerides in IDL	0.0202	3.68E-11	1.11E+07	0.7038	0.8327	0.0016	-0.0042	0.0074	0.5918	0.8311
Concentration of Large LDL Particles	NA	NA	NA	NA	NA	1.56E-05	-1.75E-05	4.87E-05	0.3546	0.7818
Total Lipids in Large LDL	0.2782	0.0582	1.3296	0.1089	0.4426	0.0217	-0.0459	0.0893	0.5291	0.8082
Phospholipids in Large LDL	0.0017	3.23E-07	8.6462	0.1428	0.4755	0.0069	-0.0079	0.0217	0.3605	0.7818
Cholesterol in Large LDL	0.1821	0.0228	1.4570	0.1084	0.4426	0.0133	-0.0369	0.0635	0.6046	0.8311
Cholesteryl Esters in Large LDL	0.0857	0.0050	1.4806	0.0910	0.4049	0.0072	-0.0302	0.0445	0.7061	0.8606
Free Cholesterol in Large LDL	0.0072	6.23E-07	84.1161	0.3021	0.5046	0.0061	-0.0073	0.0194	0.3715	0.7818
Triglycerides in Large LDL	0.0047	4.13E-12	5.36E+06	0.6145	0.7658	0.0015	-0.0043	0.0073	0.6054	0.8311
Concentration of Medium LDL Particles	NA	NA	NA	NA	NA	5.60E-06	-8.82E-06	2.00E-05	0.4463	0.7859
Total Lipids in Medium LDL	0.0169	2.52E-04	1.1265	0.0569	0.3278	0.0066	-0.0248	0.0380	0.6784	0.8532
Phospholipids in Medium LDL	1.61E-07	8.85E-14	0.2945	0.0335	0.2443	0.0018	-0.0062	0.0097	0.6636	0.8532

Cholesterol in Medium LDL	0.0025	1.29E-05	0.4949	0.0263	0.1979	0.0047	-0.0175	0.0269	0.6752	0.8532
Cholesteryl Esters in Medium LDL	0.0002	5.97E-08	0.7608	0.0428	0.2829	0.0026	-0.0141	0.0193	0.7616	0.8820
Free Cholesterol in Medium LDL	2.23E-08	3.69E-16	1.3446	0.0539	0.3183	0.0022	-0.0038	0.0081	0.4780	0.7882
Triglycerides in Medium LDL	2.94E-10	1.79E-35	4.83E+15	0.4588	0.6428	0.0001	-0.0022	0.0025	0.9087	0.9597
Concentration of Small LDL Particles	NA	NA	NA	NA	NA	3.04E-06	-4.08E-06	1.02E-05	0.4024	0.7859
Total Lipids in Small LDL	0.0004	6.16E-08	2.6485	0.0814	0.3808	0.0043	-0.0083	0.0168	0.5073	0.7995
Phospholipids in Small LDL	4.86E-10	2.29E-22	1.03E+03	0.1386	0.4710	0.0016	-0.0020	0.0053	0.3764	0.7818
Cholesterol in Small LDL	3.39E-06	5.74E-12	1.9952	0.0632	0.3405	0.0028	-0.0056	0.0112	0.5144	0.8006
Cholesteryl Esters in Small LDL	1.82E-08	1.74E-16	1.8907	0.0584	0.3278	0.0016	-0.0047	0.0079	0.6195	0.8366
Free Cholesterol in Small LDL	3.40E-15	6.33E-37	1.82E+07	0.1919	0.4902	0.0012	-0.0012	0.0036	0.3295	0.7818
Triglycerides in Small LDL	3.61E-23	6.60E-75	1.98E+29	0.3952	0.5800	-0.0002	-0.0014	0.0011	0.7754	0.8939
Concentration of Very Large HDL Particles	NA	NA	NA	NA	NA	0.0000	-2.75E-06	2.09E-05	0.1326	0.6370
Total Lipids in Very Large HDL	3.37E+08	8.41E+05	1.35E+11	1.36E-10	3.75E-09	0.0075	-0.0023	0.0173	0.1328	0.6370
Phospholipids in Very Large HDL	9.43E+15	2.20E+11	4.04E+20	1.39E-11	4.93E-10	0.0040	-0.0015	0.0095	0.1584	0.6370
Cholesterol in Very Large HDL	1.12E+20	8.94E+13	1.39E+26	1.15E-10	3.58E-09	0.0036	-0.0007	0.0079	0.1035	0.6370
Cholesteryl Esters in Very Large HDL	9.28E+26	1.93E+19	4.46E+34	5.96E-12	2.96E-10	0.0027	-0.0005	0.0058	0.0963	0.6370

Free Cholesterol in Very Large HDL	2.16E +75	4.09E+3 3	1.14E+11 7	0.0004	0.0100	0.0009	-0.0003	0.0020	0.1324	0.6370
Triglycerides in Very Large HDL	6.24E +20	6.08E-50	6.40E+90	0.5605	0.7393	0.0000	-0.0006	0.0006	0.9930	0.9930
Concentration of Large HDL Particles	NA	NA	NA	NA	NA	3.77E-05	-1.52E-0 5	0.0001	0.1620	0.6370
Total Lipids in Large HDL	5.77E +04	1.13E+0 2	2.96E+07	0.0006	0.0129	0.0135	-0.0081	0.0351	0.2214	0.6979
Phospholipids in Large HDL	3.43E +10	1.88E+0 4	6.27E+16	0.0010	0.0142	0.0043	-0.0054	0.0140	0.3812	0.7844
Cholesterol in Large HDL	3.28E +08	2.23E+0 7	4.82E+09	2.09E-4 6	5.19E-44	0.0097	-0.0027	0.0221	0.1253	0.6370
Cholesteryl Esters in Large HDL	8.65E +10	4.57E+0 4	1.64E+17	0.0006	0.0132	0.0077	-0.0020	0.0173	0.1192	0.6370
Free Cholesterol in Large HDL	9.85E +33	4.61E+2 8	2.11E+39	7.41E-3 6	6.12E-34	0.0020	-0.0009	0.0050	0.1771	0.6370
Triglycerides in Large HDL	1.19E +11	4.73E-08	2.98E+29	0.2381	0.4923	-0.000 5	-0.0029	0.0019	0.6740	0.8532
Concentration of Medium HDL Particles	NA	NA	NA	NA	NA	-0.000 1	-0.0001	1.40E-05	0.1147	0.6370
Total Lipids in Medium HDL	0.0002	1.49E-07	0.3339	0.0242	0.1876	-0.017 4	-0.0384	0.0037	0.1053	0.6370
Phospholipids in Medium HDL	1.70E-06	3.78E-12	0.7671	0.0455	0.2829	-0.009 3	-0.0206	0.0021	0.1085	0.6370
Cholesterol in Medium HDL	3.69E-13	6.80E-15	2.01E-11	8.22E-4 5	1.02E-42	-0.006 2	-0.0134	0.0009	0.0877	0.6370
Cholesteryl Esters in Medium HDL	3.72E-14	3.21E-23	4.32E-05	0.0037	0.0381	-0.005 3	-0.0114	0.0007	0.0840	0.6370
Free Cholesterol in Medium HDL	1.76E-51	1.38E-61	2.24E-41	7.20E-2 3	4.47E-21	-0.000 9	-0.0025	0.0006	0.2422	0.7095
Triglycerides in Medium HDL	0.0001	1.13E-19	2.91E+10	0.5719	0.7464	-0.001 9	-0.0059	0.0022	0.3723	0.7818

Concentration of Small HDL Particles	NA	NA	NA	NA	NA	-0.0002	-0.0004	0.0001	0.1868	0.6370
Total Lipids in Small HDL	0.0004	2.47E-06	0.0512	0.0017	0.0205	-0.0233	-0.0524	0.0058	0.1160	0.6370
Phospholipids in Small HDL	3.45E-06	7.75E-10	0.0153	0.0033	0.0360	-0.0148	-0.0318	0.0022	0.0871	0.6370
Cholesterol in Small HDL	7.06E-10	2.24E-15	0.0002	0.0011	0.0142	-0.0070	-0.0176	0.0035	0.1915	0.6370
Cholesteryl Esters in Small HDL	5.45E-11	3.56E-17	0.0001	0.0011	0.0142	-0.0056	-0.0140	0.0028	0.1919	0.6370
Free Cholesterol in Small HDL	4.03E-34	1.54E-58	1.05E-09	0.0073	0.0710	-0.0014	-0.0042	0.0013	0.3088	0.7818
Triglycerides in Small HDL	5.18E-13	8.41E-33	3.19E+07	0.2237	0.4923	-0.0015	-0.0050	0.0021	0.4201	0.7859
Phospholipids to Total Lipids in Chylomicrons and Extremely Large VLDL percentage	0.9278	0.7747	1.1112	0.4157	0.6028	-0.0850	-0.7863	0.6162	0.8121	0.9182
Cholesterol to Total Lipids in Chylomicrons and Extremely Large VLDL percentage	0.9455	0.8554	1.0452	0.2730	0.5045	1.4288	-0.8375	3.6950	0.2164	0.6909
Cholesteryl Esters to Total Lipids in Chylomicrons and Extremely Large VLDL percentage	0.9281	0.8079	1.0661	0.2914	0.5046	0.6834	-1.0025	2.3693	0.4267	0.7859
Free Cholesterol to Total Lipids in Chylomicrons and Extremely Large VLDL percentage	0.8720	0.6705	1.1340	0.3069	0.5050	0.7453	-0.0370	1.5277	0.0619	0.6065

Triglycerides to Total Lipids in Chylomicrons and Extremely Large VLDL percentage	1.0440	0.9719	1.1216	0.2384	0.4923	-1.3437	-3.9197	1.2323	0.3064	0.7818
Phospholipids to Total Lipids in Very Large VLDL percentage	0.9389	0.7293	1.2087	0.6248	0.7747	0.1941	-0.2737	0.6619	0.4159	0.7859
Cholesterol to Total Lipids in Very Large VLDL percentage	1.0231	0.9152	1.1438	0.6881	0.8283	1.2754	0.0735	2.4774	0.0376	0.5066
Cholesteryl Esters to Total Lipids in Very Large VLDL percentage	1.0184	0.8835	1.1740	0.8011	0.8714	0.9459	-0.0098	1.9016	0.0524	0.5672
Free Cholesterol to Total Lipids in Very Large VLDL percentage	1.2106	0.7759	1.8888	0.3997	0.5832	0.3295	0.0236	0.6354	0.0347	0.5066
Triglycerides to Total Lipids in Very Large VLDL percentage	0.9913	0.8994	1.0925	0.8595	0.9080	-1.4696	-2.8624	-0.0768	0.0387	0.5066
Phospholipids to Total Lipids in Large VLDL percentage	0.8934	0.7145	1.1170	0.3226	0.5113	-0.0335	-0.5354	0.4684	0.8958	0.9574
Cholesterol to Total Lipids in Large VLDL percentage	0.9993	0.8602	1.1609	0.9929	0.9929	0.8795	0.0239	1.7351	0.0439	0.5392
Cholesteryl Esters to Total Lipids in Large VLDL percentage	1.0317	0.8479	1.2554	0.7552	0.8629	0.6956	0.0528	1.3385	0.0339	0.5066

Free Cholesterol to Total Lipids in Large VLDL percentage	0.8235	0.5038	1.3459	0.4384	0.6229	0.1838	-0.0733	0.4409	0.1611	0.6370
Triglycerides to Total Lipids in Large VLDL percentage	1.0238	0.9133	1.1477	0.6866	0.8283	-0.8460	-1.9895	0.2975	0.1469	0.6370
Phospholipids to Total Lipids in Medium VLDL percentage	1.0813	0.7826	1.4940	0.6358	0.7844	0.5058	0.1559	0.8556	0.0046	0.3294
Cholesterol to Total Lipids in Medium VLDL percentage	1.0276	0.9297	1.1359	0.5939	0.7612	1.6176	0.4107	2.8246	0.0086	0.3589
Cholesteryl Esters to Total Lipids in Medium VLDL percentage	1.0363	0.9046	1.1872	0.6069	0.7612	1.1614	0.2685	2.0543	0.0108	0.3851
Free Cholesterol to Total Lipids in Medium VLDL percentage	1.1136	0.7692	1.6122	0.5687	0.7463	0.4563	0.1308	0.7817	0.0060	0.3294
Triglycerides to Total Lipids in Medium VLDL percentage	0.9793	0.9061	1.0584	0.5983	0.7612	-2.1234	-3.6551	-0.5917	0.0066	0.3294
Phospholipids to Total Lipids in Small VLDL percentage	0.9154	0.6533	1.2828	0.6078	0.7612	0.4089	0.0512	0.7665	0.0251	0.4784
Cholesterol to Total Lipids in Small VLDL percentage	0.9975	0.8699	1.1439	0.9716	0.9821	1.0407	0.1532	1.9283	0.0216	0.4784
Cholesteryl Esters to Total Lipids in Small VLDL percentage	1.0209	0.8109	1.2854	0.8601	0.9080	0.6093	0.0698	1.1488	0.0269	0.4784

Free Cholesterol to Total Lipids in Small VLDL percentage	0.9487	0.6912	1.3021	0.7444	0.8586	0.4315	0.0511	0.8120	0.0262	0.4784
Triglycerides to Total Lipids in Small VLDL percentage	1.0089	0.9145	1.1130	0.8604	0.9080	-1.4496	-2.6798	-0.2195	0.0209	0.4784
Phospholipids to Total Lipids in Very Small VLDL percentage	1.9284	0.8996	4.1340	0.0914	0.4049	-0.0712	-0.2134	0.0711	0.3268	0.7818
Cholesterol to Total Lipids in Very Small VLDL percentage	1.0271	0.8802	1.1987	0.7339	0.8505	0.7955	-0.0442	1.6352	0.0633	0.6065
Cholesteryl Esters to Total Lipids in Very Small VLDL percentage	1.0256	0.8600	1.2230	0.7784	0.8668	0.6966	-0.0346	1.4278	0.0618	0.6065
Free Cholesterol to Total Lipids in Very Small VLDL percentage	1.3832	0.4833	3.9588	0.5455	0.7273	0.0989	-0.0326	0.2304	0.1404	0.6370
Triglycerides to Total Lipids in Very Small VLDL percentage	0.9409	0.7916	1.1184	0.4897	0.6732	-0.7244	-1.5046	0.0559	0.0688	0.6118
Phospholipids to Total Lipids in IDL percentage	1.8953	0.8846	4.0608	0.1000	0.4277	-0.0766	-0.2412	0.0880	0.3615	0.7818
Cholesterol to Total Lipids in IDL percentage	0.9646	0.8067	1.1534	0.6926	0.8298	0.2748	-0.3134	0.8631	0.3596	0.7818

Cholesteryl Esters to Total Lipids in IDL percentage	0.9130	0.7453	1.1185	0.3796	0.5648	0.1222	-0.3386	0.5830	0.6032	0.8311
Free Cholesterol to Total Lipids in IDL percentage	1.3119	0.7283	2.3631	0.3659	0.5533	0.1527	-0.0840	0.3893	0.2060	0.6748
Triglycerides to Total Lipids in IDL percentage	0.9980	0.8059	1.2358	0.9850	0.9890	-0.1983	-0.7166	0.3201	0.4533	0.7859
Phospholipids to Total Lipids in Large LDL percentage	1.0500	0.4564	2.4155	0.9087	0.9429	0.1239	-0.0264	0.2741	0.1060	0.6370
Cholesterol to Total Lipids in Large LDL percentage	0.9664	0.7774	1.2014	0.7585	0.8629	-0.1126	-0.4766	0.2514	0.5442	0.8145
Cholesteryl Esters to Total Lipids in Large LDL percentage	0.8879	0.7606	1.0366	0.1324	0.4690	-0.2925	-0.5652	-0.0199	0.0355	0.5066
Free Cholesterol to Total Lipids in Large LDL percentage	1.3281	0.7660	2.3027	0.3122	0.5087	0.1800	-0.0784	0.4384	0.1721	0.6370
Triglycerides to Total Lipids in Large LDL percentage	1.0313	0.8252	1.2888	0.7865	0.8668	-0.0113	-0.4089	0.3864	0.9558	0.9714
Phospholipids to Total Lipids in Medium LDL percentage	1.9621	0.8106	4.7494	0.1351	0.4710	0.0288	-0.1188	0.1763	0.7020	0.8606
Cholesterol to Total Lipids in Medium LDL percentage	0.8188	0.6223	1.0773	0.1533	0.4755	0.0726	-0.2655	0.4106	0.6738	0.8532

Cholesteryl Esters to Total Lipids in Medium LDL percentage	0.6766	0.5352	0.8555	0.0011	0.0142	-0.1410	-0.4930	0.2111	0.4324	0.7859
Free Cholesterol to Total Lipids in Medium LDL percentage	1.1971	0.8420	1.7020	0.3162	0.5087	0.2134	-0.1625	0.5893	0.2656	0.7515
Triglycerides to Total Lipids in Medium LDL percentage	1.1371	0.8530	1.5157	0.3812	0.5648	-0.1013	-0.4375	0.2349	0.5545	0.8145
Phospholipids to Total Lipids in Small LDL percentage	1.5350	1.0575	2.2281	0.0242	0.1876	0.1441	-0.1894	0.4775	0.3969	0.7844
Cholesterol to Total Lipids in Small LDL percentage	0.7979	0.6032	1.0555	0.1137	0.4478	0.0830	-0.3079	0.4740	0.6771	0.8532
Cholesteryl Esters to Total Lipids in Small LDL percentage	0.6896	0.5026	0.9461	0.0213	0.1819	-0.1513	-0.5450	0.2425	0.4512	0.7859
Free Cholesterol to Total Lipids in Small LDL percentage	1.0558	0.7841	1.4217	0.7206	0.8430	0.2343	-0.1867	0.6553	0.2751	0.7529
Triglycerides to Total Lipids in Small LDL percentage	0.9941	0.7226	1.3675	0.9710	0.9821	-0.2270	-0.6044	0.1503	0.2382	0.7060
Phospholipids to Total Lipids in Very Large HDL percentage	1.2666	0.9985	1.6066	0.0514	0.3111	0.9033	-0.4050	2.2116	0.1758	0.6370

Cholesterol to Total Lipids in Very Large HDL percentage	0.9303	0.8205	1.0549	0.2598	0.4973	-0.4034	-1.4946	0.6879	0.4686	0.7859
Cholesteryl Esters to Total Lipids in Very Large HDL percentage	0.9236	0.8048	1.0600	0.2582	0.4973	-0.0568	-0.8283	0.7147	0.8851	0.9574
Free Cholesterol to Total Lipids in Very Large HDL percentage	0.8975	0.6352	1.2683	0.5400	0.7267	-0.3465	-0.9132	0.2202	0.2306	0.7030
Triglycerides to Total Lipids in Very Large HDL percentage	0.7941	0.5702	1.1060	0.1726	0.4881	-0.5000	-1.6022	0.6023	0.3738	0.7818
Phospholipids to Total Lipids in Large HDL percentage	0.9721	0.8744	1.0809	0.6015	0.7612	-0.9285	-1.5542	-0.3029	0.0037	0.3294
Cholesterol to Total Lipids in Large HDL percentage	1.0711	0.9267	1.2379	0.3526	0.5397	0.4078	-0.6182	1.4338	0.4357	0.7859
Cholesteryl Esters to Total Lipids in Large HDL percentage	1.0626	0.9084	1.2430	0.4478	0.6310	0.3207	-0.5807	1.2222	0.4854	0.7900
Free Cholesterol to Total Lipids in Large HDL percentage	1.4625	0.7738	2.7640	0.2418	0.4923	0.0871	-0.1474	0.3215	0.4665	0.7859
Triglycerides to Total Lipids in Large HDL percentage	0.9638	0.7814	1.1889	0.7308	0.8505	0.5207	-0.1487	1.1901	0.1273	0.6370

Phospholipids to Total Lipids in Medium HDL percentage	1.2303	0.6852	2.2090	0.4877	0.6732	-0.498 8	-0.7874	-0.2101	0.0007	0.1786
Cholesterol to Total Lipids in Medium HDL percentage	0.9796	0.8440	1.1371	0.7869	0.8668	-0.120 7	-0.6117	0.3702	0.6296	0.8366
Cholesteryl Esters to Total Lipids in Medium HDL percentage	0.9841	0.8328	1.1630	0.8510	0.9080	-0.142 4	-0.6294	0.3446	0.5665	0.8181
Free Cholesterol to Total Lipids in Medium HDL percentage	0.7357	0.2916	1.8566	0.5158	0.6990	0.0216	-0.0443	0.0875	0.5209	0.8057
Triglycerides to Total Lipids in Medium HDL percentage	0.9938	0.8998	1.0975	0.9016	0.9395	0.6195	0.1186	1.1205	0.0154	0.4563
Phospholipids to Total Lipids in Small HDL percentage	1.5887	0.8996	2.8056	0.1107	0.4426	-0.149 9	-0.3692	0.0694	0.1801	0.6370
Cholesterol to Total Lipids in Small HDL percentage	0.9228	0.7529	1.1312	0.4395	0.6229	0.0200	-0.3700	0.4100	0.9199	0.9597
Cholesteryl Esters to Total Lipids in Small HDL percentage	0.9198	0.7742	1.0929	0.3420	0.5301	-0.073 7	-0.4869	0.3396	0.7267	0.8742
Free Cholesterol to Total Lipids in Small HDL percentage	1.7356	0.7424	4.0575	0.2032	0.4902	0.0937	-0.0066	0.1940	0.0671	0.6118
Triglycerides to Total Lipids in Small HDL percentage	1.0039	0.8150	1.2364	0.9711	0.9821	0.1299	-0.1222	0.3821	0.3123	0.7818

[a]Models were adjusted for age, sex, ethnicity, Townsend deprivation index, smoking status, alcohol consumption, physical activity, education, waist circumference, hypertension status, diabetes status, ALT， HDL, and myopia status.

[b]Models were adjusted for age, sex, ethnicity, Townsend deprivation index, smoking status, alcohol consumption, physical activity, education, waist circumference, hypertension status, diabetes status, ALT and HDL.

HR Hazards ratio, CI Confidence interval, ALT Alanine aminotransferase, LDL Low-density lipoprotein, VLDL Very low-density lipoprotein, IDL Intermediate-density lipoprotein, HDL High-density lipoprotein, HDL-C High-density lipoprotein cholesterol.

NA denotes statistical models with convergence failure or sparse data.

Table S15. Selection of metabolites as potential mediators between high myopia and incident autoimmune hepatitis

Metabolites	HR[a]	CI_lower	CI_upper	P	FDR	Beta[b]	CI_lower	CI_upper	P	FDR
Total Cholesterol	1.5022	0.7024	3.2128	0.2941	0.9270	0.0607	-0.0928	0.2143	0.4380	0.7859
Total Cholesterol Minus HDL-C	1.5022	0.7024	3.2128	0.2941	0.9270	0.0607	-0.0928	0.2143	0.4380	0.7859
Remnant Cholesterol (Non-HDL, Non-LDL -Cholesterol)	2.2766	0.4988	10.3913	0.2882	0.9270	0.0399	-0.0364	0.1163	0.3052	0.7818
VLDL Cholesterol	3.1453	0.2417	40.9375	0.3814	0.9270	0.0149	-0.0313	0.0611	0.5262	0.8082
Clinical LDL Cholesterol	1.6377	0.6705	4.0002	0.2789	0.9270	0.0625	-0.0702	0.1951	0.3558	0.7818
LDL Cholesterol	2.1296	0.4864	9.3243	0.3157	0.9270	0.0208	-0.0593	0.1009	0.6108	0.8311
HDL Cholesterol	NA	NA	NA	NA	NA	3.61E-17	-2.29E-17	9.51E-17	0.2306	0.7030
Total Triglycerides	1.1833	0.3775	3.7091	0.7728	0.9270	-0.0415	-0.1638	0.0808	0.5062	0.7995
Triglycerides in VLDL	1.2200	0.3008	4.9485	0.7807	0.9270	-0.0407	-0.1414	0.0600	0.4283	0.7859
Triglycerides in LDL	33.0763	0.0002	7.18E+06	0.5768	0.9270	0.0015	-0.0078	0.0107	0.7550	0.8786
Triglycerides in HDL	0.2902	3.12E-07	2.70E+05	0.8599	0.9661	-0.0038	-0.0142	0.0065	0.4651	0.7859
Total Phospholipids in Lipoprotein Particles	1.9132	0.3666	9.9830	0.4415	0.9270	0.0033	-0.0715	0.0781	0.9318	0.9628
Phospholipids in VLDL	3.0928	0.0894	107.0320	0.5324	0.9270	0.0014	-0.0338	0.0367	0.9375	0.9646
Phospholipids in LDL	12.0228	0.1265	1.14E+03	0.2846	0.9270	0.0103	-0.0157	0.0363	0.4360	0.7859
Phospholipids in HDL	0.2959	0.0005	193.8530	0.7128	0.9270	-0.0158	-0.0385	0.0069	0.1713	0.6370
Total Esterified Cholesterol	1.7924	0.6010	5.3456	0.2952	0.9270	0.0401	-0.0669	0.1471	0.4624	0.7859
Cholesteryl Esters in VLDL	8.6568	0.1065	7.04E+02	0.3361	0.9270	0.0121	-0.0144	0.0386	0.3720	0.7818
Cholesteryl Esters in LDL	2.6728	0.3678	19.4217	0.3313	0.9270	0.0114	-0.0482	0.0710	0.7085	0.8606
Cholesteryl Esters in HDL	2.75E-07	4.49E-21	1.68E+07	0.3510	0.9270	-0.0006	-0.0041	0.0029	0.7468	0.8786
Total Free Cholesterol	3.7344	0.3152	44.2455	0.2962	0.9270	0.0206	-0.0264	0.0676	0.3897	0.7844
Free Cholesterol in VLDL	9.1449	0.0233	3582.0166	0.4675	0.9270	0.0029	-0.0176	0.0233	0.7847	0.8963

Free Cholesterol in LDL	19.8454	0.0772	5098.9165	0.2912	0.9270	0.0094	-0.0121	0.0310	0.3910	0.7844
Free Cholesterol in HDL	3.61E+06	5.87E-08	2.22E+20	0.3513	0.9270	0.0006	-0.0029	0.0041	0.7477	0.8786
Total Lipids in Lipoprotein Particles	1.1821	0.7988	1.7494	0.4028	0.9270	0.0225	-0.2875	0.3326	0.8866	0.9574
Total Lipids in VLDL	1.2368	0.5818	2.6290	0.5807	0.9270	-0.0243	-0.1963	0.1477	0.7814	0.8963
Total Lipids in LDL	1.7419	0.5990	5.0658	0.3082	0.9270	0.0326	-0.0781	0.1432	0.5636	0.8181
Total Lipids in HDL	0.4762	0.0049	46.0112	0.7504	0.9270	-0.0197	-0.0520	0.0127	0.2329	0.7030
Total Concentration of Lipoprotein Particles	1.9495	0.0000	2.22E+190	0.9976	0.9979	-0.0001	-0.0004	0.0001	0.3390	0.7818
Concentration of VLDL Particles	NA	NA	NA	NA	NA	2.08E-06	-6.61E-06	1.08E-05	0.6392	0.8421
Concentration of LDL Particles	NA	NA	NA	NA	NA	2.43E-05	-2.91E-05	0.0001	0.3730	0.7818
Concentration of HDL Particles	1.71E-36	4.31E-249	6.76E+176	0.7416	0.9270	-0.0002	-0.0004	0.0001	0.1531	0.6370
Average Diameter for VLDL Particles	1.0165	0.5417	1.9075	0.9592	0.9836	-0.2104	-0.4186	-0.0022	0.0476	0.5392
Average Diameter for LDL Particles	0.4866	0.0001	2.58E+03	0.8692	0.9661	0.0060	-0.0099	0.0219	0.4597	0.7859
Average Diameter for HDL Particles	4.8799	0.0499	4.78E+02	0.4979	0.9270	0.0163	-0.0064	0.0390	0.1589	0.6370
Phosphoglycerides	1.4023	0.1716	11.4590	0.7524	0.9270	-0.0114	-0.0724	0.0496	0.7145	0.8637
Triglycerides to Phosphoglycerides ratio	1.0777	0.0207	56.1245	0.9704	0.9836	-0.0201	-0.0560	0.0158	0.2720	0.7529
Total Cholines	1.7704	0.2086	15.0242	0.6006	0.9270	-0.0041	-0.0625	0.0543	0.8908	0.9574
Phosphatidylcholines	0.8281	0.0844	8.1234	0.8714	0.9661	-0.0088	-0.0637	0.0461	0.7533	0.8786
Sphingomyelins	4.06E+04	0.2646	6.23E+09	0.0816	0.9270	0.0066	-0.0032	0.0164	0.1875	0.6370
Apolipoprotein B	5.5090	0.2554	1.19E+02	0.2762	0.9270	0.0194	-0.0181	0.0569	0.3106	0.7818
Apolipoprotein A1	0.1194	0.0001	2.29E+02	0.5815	0.9270	-0.0142	-0.0311	0.0027	0.1000	0.6370
Apolipoprotein B to Apolipoprotein A1 ratio	1.8553	0.1846	18.6417	0.5996	0.9270	0.0360	0.0066	0.0654	0.0165	0.4563

Total Fatty Acids	1.0913	0.8394	1.4187	0.5141	0.9270	-0.0883	-0.5916	0.4149	0.7307	0.8747
Degree of Unsaturation	3.8997	0.0004	3.89E+04	0.7721	0.9270	0.0046	-0.0100	0.0191	0.5374	0.8109
Omega-3 Fatty Acids	1.7825	0.0777	40.8790	0.7176	0.9270	0.0014	-0.0416	0.0444	0.9480	0.9691
Omega-6 Fatty Acids	1.4861	0.5355	4.1240	0.4469	0.9270	-0.0039	-0.1238	0.1160	0.9497	0.9691
Polyunsaturated Fatty Acids	1.3986	0.5817	3.3625	0.4536	0.9270	-0.0024	-0.1450	0.1401	0.9733	0.9812
Monounsaturated Fatty Acids	1.2330	0.5820	2.6123	0.5845	0.9270	-0.0289	-0.2109	0.1530	0.7551	0.8786
Saturated Fatty Acids	1.2093	0.6386	2.2899	0.5596	0.9270	-0.0570	-0.2638	0.1499	0.5893	0.8311
Linoleic Acid	1.3450	0.4993	3.6230	0.5577	0.9270	0.0059	-0.1171	0.1290	0.9250	0.9597
Docosahexaenoic Acid	1.2195	0.0002	9.64E+03	0.9654	0.9836	0.0020	-0.0134	0.0175	0.7985	0.9079
Omega-3 Fatty Acids to Total Fatty Acids percentage	0.9881	0.6048	1.6145	0.9620	0.9836	0.0167	-0.2773	0.3107	0.9112	0.9597
Omega-6 Fatty Acids to Total Fatty Acids percentage	0.9712	0.7960	1.1849	0.7731	0.9270	0.2870	-0.4182	0.9921	0.4248	0.7859
Polyunsaturated Fatty Acids to Total Fatty Acids percentage	0.9715	0.8023	1.1764	0.7675	0.9270	0.3037	-0.4158	1.0231	0.4078	0.7859
Monounsaturated Fatty Acids to Total Fatty Acids percentage	1.0547	0.7893	1.4094	0.7186	0.9270	-0.1320	-0.6069	0.3429	0.5857	0.8311
Saturated Fatty Acids to Total Fatty Acids percentage	1.0198	0.7069	1.4712	0.9164	0.9712	-0.1717	-0.5526	0.2092	0.3768	0.7818
Linoleic Acid to Total Fatty Acids percentage	0.9630	0.7727	1.2002	0.7373	0.9270	0.3193	-0.2947	0.9334	0.3079	0.7818
Docosahexaenoic Acid to Total Fatty Acids percentage	0.7336	0.2124	2.53E+00	0.6243	0.9270	0.0204	-0.1073	0.1481	0.7541	0.8786
Polyunsaturated Fatty Acids to Monounsaturated Fatty Acids ratio	0.5313	0.0506	5.58E+00	0.5982	0.9270	0.0256	-0.0328	0.0839	0.3902	0.7844

Omega-6 Fatty Acids to Omega-3 Fatty Acids ratio	0.9898	0.8262	1.19E+00	0.9111	0.9702	0.3354	-0.5167	1.1875	0.4402	0.7859
Alanine	9.5973	0.0029	3.20E+04	0.5848	0.9270	-0.0152	-0.0302	-0.0002	0.0466	0.5392
Glutamine	0.0664	1.87E-05	2.35E+02	0.5153	0.9270	0.0036	-0.0127	0.0200	0.6619	0.8532
Glycine	54.1635	0.0014	2.17E+06	0.4603	0.9270	-0.0011	-0.0108	0.0086	0.8210	0.9209
Histidine	2.25E+25	172.9995	2.92E+48	0.0316	0.9270	-0.0005	-0.0024	0.0014	0.6283	0.8366
Total Concentration of Branched-Chain Amino Acids (Leucine + Isoleucine + Valine)	28.9633	0.0153	5.47E+04	0.3818	0.9270	-0.0044	-0.0191	0.0103	0.5561	0.8145
Isoleucine	1.03E+05	0.0000	1.07E+21	0.5394	0.9270	0.0002	-0.0028	0.0033	0.8928	0.9574
Leucine	6.37E+03	0.0000	1.72E+14	0.4747	0.9270	-0.0011	-0.0059	0.0038	0.6599	0.8532
Valine	1.91E+03	0.0007	5.02E+09	0.3167	0.9270	-0.0035	-0.0111	0.0040	0.3558	0.7818
Phenylalanine	4.29E+07	0.0000	9.95E+21	0.2977	0.9270	-0.0015	-0.0038	0.0007	0.1883	0.6370
Tyrosine	9.02E+13	0.0353	2.31E+29	0.0759	0.9270	0.0003	-0.0025	0.0031	0.8454	0.9398
Glucose	1.4604	0.8534	2.50E+00	0.1671	0.9270	0.1021	-0.1151	0.3194	0.3565	0.7818
Lactate	1.0763	0.6444	1.80E+00	0.7788	0.9270	-0.1872	-0.4267	0.0523	0.1255	0.6370
Pyruvate	0.0001	0.0000	8.38E+05	0.4335	0.9270	-0.0004	-0.0070	0.0063	0.9159	0.9597
Citrate	4.08E+18	0.0636	2.61E+38	0.0655	0.9270	0.0005	-0.0020	0.0030	0.6991	0.8606
3-Hydroxybutyrate	0.1020	6.91E-07	1.50E+04	0.7069	0.9270	0.0071	-0.0051	0.0193	0.2529	0.7322
Acetate	0.3774	1.44E-24	9.90E+22	0.9717	0.9836	-0.0007	-0.0031	0.0017	0.5531	0.8145
Acetoacetate	0.0025	5.33E-25	1.13E+19	0.8133	0.9470	0.0011	-0.0011	0.0032	0.3221	0.7818
Acetone	3.97E+04	1.14E-43	1.38E+52	0.8496	0.9661	0.0001	-0.0010	0.0012	0.9169	0.9597
Creatinine	1.46E+06	7.08E-15	3.03E+26	0.5520	0.9270	-0.0016	-0.0039	0.0006	0.1604	0.6370
Albumin	1.0170	0.8411	1.23E+00	0.8618	0.9661	-0.4367	-1.0891	0.2157	0.1894	0.6370
Glycoprotein Acetyls	1.59E+03	8.2298	3.07E+05	0.0061	0.9270	0.0011	-0.0207	0.0229	0.9234	0.9597

Concentration of Chylomicrons and Extremely Large VLDL Particles	NA	NA	NA	NA	NA	-1.11E-07	-4.21E-07	1.99E-07	0.4815	0.7888
Total Lipids in Chylomicrons and Extremely Large VLDL	0.9909	0.0227	4.32E+01	0.9962	0.9979	-0.0164	-0.0586	0.0258	0.4451	0.7859
Phospholipids in Chylomicrons and Extremely Large VLDL	0.0436	3.59E-13	5.29E+09	0.8099	0.9470	-0.0022	-0.0086	0.0042	0.4942	0.7939
Cholesterol in Chylomicrons and Extremely Large VLDL	3.6975	8.80E-08	1.55E+08	0.8839	0.9661	-0.0021	-0.0104	0.0063	0.6250	0.8366
Cholesteryl Esters in Chylomicrons and Extremely Large VLDL	670.2921	4.19E-11	1.07E+16	0.6748	0.9270	-0.0009	-0.0055	0.0036	0.6932	0.8606
Free Cholesterol in Chylomicrons and Extremely Large VLDL	0.0155	2.02E-20	1.18E+16	0.8427	0.9661	-0.0012	-0.0050	0.0027	0.5531	0.8145
Triglycerides in Chylomicrons and Extremely Large VLDL	1.0077	0.0030	3.41E+02	0.9979	0.9979	-0.0121	-0.0400	0.0158	0.3938	0.7844
Concentration of Very Large VLDL Particles	NA	NA	NA	NA	NA	-1.51E-07	-6.02E-07	3.01E-07	0.5134	0.8006
Total Lipids in Very Large VLDL	2.6044	0.0143	4.73E+02	0.7183	0.9270	-0.0098	-0.0365	0.0170	0.4743	0.7873
Phospholipids in Very Large VLDL	1.27E+02	1.51E-10	1.08E+14	0.7294	0.9270	-0.0013	-0.0064	0.0037	0.6104	0.8311
Cholesterol in Very Large VLDL	7.89E+03	2.72E-07	2.29E+14	0.4653	0.9270	-0.0005	-0.0058	0.0048	0.8536	0.9444
Cholesteryl Esters in Very Large VLDL	1.59E+10	1.40E-10	1.82E+30	0.3188	0.9270	3.82E-05	-0.0026	0.0027	0.9772	0.9812
Free Cholesterol in Very Large VLDL	4.01E+04	1.58E-17	1.02E+26	0.6734	0.9270	-0.0005	-0.0033	0.0022	0.7032	0.8606
Triglycerides in Very Large VLDL	2.6767	0.0005	1.43E+04	0.8221	0.9528	-0.0080	-0.0248	0.0089	0.3546	0.7818

Concentration of Large VLDL Particles	NA	NA	NA	NA	NA	-3.17E-07	-1.41E-06	7.74E-07	0.5684	0.8181
Total Lipids in Large VLDL	2.5657	0.0617	1.07E+02	0.6204	0.9270	-0.0122	-0.0472	0.0228	0.4939	0.7939
Phospholipids in Large VLDL	38.9927	8.74E-07	1.74E+09	0.6835	0.9270	-0.0021	-0.0097	0.0055	0.5828	0.8311
Cholesterol in Large VLDL	126.6258	0.0001	1.90E+08	0.5047	0.9270	-0.0007	-0.0094	0.0081	0.8811	0.9574
Cholesteryl Esters in Large VLDL	7.19E+04	2.79E-08	1.85E+17	0.4431	0.9270	0.0004	-0.0038	0.0046	0.8572	0.9444
Free Cholesterol in Large VLDL	2.41E+03	2.37E-09	2.45E+15	0.5809	0.9270	-0.0011	-0.0057	0.0036	0.6585	0.8532
Triglycerides in Large VLDL	4.4389	0.0046	4.30E+03	0.6710	0.9270	-0.0094	-0.0287	0.0099	0.3381	0.7818
Concentration of Medium VLDL Particles	NA	NA	NA	NA	NA	4.64E-07	-1.86E-06	2.79E-06	0.6952	0.8606
Total Lipids in Medium VLDL	3.5282	0.1787	6.97E+01	0.4074	0.9270	0.0007	-0.0394	0.0407	0.9744	0.9812
Phospholipids in Medium VLDL	3.87E+02	0.0008	1.83E+08	0.3715	0.9270	0.0022	-0.0068	0.0112	0.6316	0.8366
Cholesterol in Medium VLDL	1.95E+02	0.0126	3.00E+06	0.2839	0.9270	0.0067	-0.0053	0.0187	0.2732	0.7529
Cholesteryl Esters in Medium VLDL	1.39E+04	0.0007	2.83E+11	0.2663	0.9270	0.0047	-0.0023	0.0117	0.1877	0.6370
Free Cholesterol in Medium VLDL	3.10E+04	1.95E-05	4.94E+13	0.3387	0.9270	0.0020	-0.0035	0.0075	0.4703	0.7859
Triglycerides in Medium VLDL	3.7977	0.0177	8.14E+02	0.6261	0.9270	-0.0083	-0.0321	0.0156	0.4974	0.7939
Concentration of Small VLDL Particles	NA	NA	NA	NA	NA	3.01E-07	-2.22E-06	0.0000	0.8149	0.9182
Total Lipids in Small VLDL	4.0011	0.0343	4.67E+02	0.5680	0.9270	0.0029	-0.0228	0.0285	0.8267	0.9230
Phospholipids in Small VLDL	4.42E+03	5.36E-06	3.65E+12	0.4229	0.9270	0.0018	-0.0039	0.0075	0.5361	0.8109
Cholesterol in Small VLDL	1.63E+02	0.0008	3.40E+07	0.4146	0.9270	0.0044	-0.0052	0.0140	0.3686	0.7818
Cholesteryl Esters in Small VLDL	1.79E+03	7.90E-06	4.04E+11	0.4455	0.9270	0.0028	-0.0033	0.0089	0.3702	0.7818
Free Cholesterol in Small VLDL	4.00E+06	1.57E-08	1.02E+21	0.3691	0.9270	0.0016	-0.0019	0.0051	0.3735	0.7818
Triglycerides in Small VLDL	2.0571	4.96E-05	8.54E+04	0.8942	0.9702	-0.0033	-0.0161	0.0094	0.6078	0.8311

Concentration of Very Small VLDL Particles	NA	NA	NA	NA	NA	1.89E-06	-7.85E-07	4.57E-06	0.1660	0.6370
Total Lipids in Very Small VLDL	14.6654	0.0273	7.86E+03	0.4023	0.9270	0.0105	-0.0068	0.0279	0.2343	0.7030
Phospholipids in Very Small VLDL	6.81E+03	1.62E-05	2.87E+12	0.3837	0.9270	0.0031	-0.0023	0.0084	0.2592	0.7420
Cholesterol in Very Small VLDL	4.19E+02	0.0018	1.00E+08	0.3392	0.9270	0.0071	-0.0019	0.0160	0.1237	0.6370
Cholesteryl Esters in Very Small VLDL	5.10E+03	0.0001	3.31E+11	0.3524	0.9270	0.0051	-0.0012	0.0113	0.1111	0.6370
Free Cholesterol in Very Small VLDL	1.55E+08	6.56E-09	3.67E+24	0.3269	0.9270	0.0020	-0.0009	0.0048	0.1743	0.6370
Triglycerides in Very Small VLDL	41.0514	1.68E-10	1.00E+13	0.7813	0.9270	0.0004	-0.0043	0.0051	0.8642	0.9480
Concentration of IDL Particles	NA	NA	NA	NA	NA	1.15E-05	-2.25E-06	2.52E-05	0.1011	0.6370
Total Lipids in IDL	3.9961	0.3896	4.10E+01	0.2435	0.9270	0.0340	-0.0149	0.0828	0.1732	0.6370
Phospholipids in IDL	3.26E+02	0.0164	6.50E+06	0.2518	0.9270	0.0074	-0.0041	0.0188	0.2094	0.6771
Cholesterol in IDL	6.7776	0.2752	1.67E+02	0.2417	0.9270	0.0250	-0.0104	0.0604	0.1664	0.6370
Cholesteryl Esters in IDL	12.7404	0.1740	9.33E+02	0.2454	0.9270	0.0174	-0.0087	0.0435	0.1908	0.6370
Free Cholesterol in IDL	1.35E+03	0.0072	2.55E+08	0.2447	0.9270	0.0076	-0.0020	0.0172	0.1212	0.6370
Triglycerides in IDL	133.8973	0.0000	2.99E+10	0.6176	0.9270	0.0016	-0.0042	0.0074	0.5918	0.8311
Concentration of Large LDL Particles	NA	NA	NA	NA	NA	1.56E-05	-1.75E-05	4.87E-05	0.3546	0.7818
Total Lipids in Large LDL	2.5415	0.4448	1.45E+01	0.2942	0.9270	0.0217	-0.0459	0.0893	0.5291	0.8082
Phospholipids in Large LDL	72.0004	0.0244	2.12E+05	0.2941	0.9270	0.0069	-0.0079	0.0217	0.3605	0.7818
Cholesterol in Large LDL	3.4953	0.3332	3.67E+01	0.2967	0.9270	0.0133	-0.0369	0.0635	0.6046	0.8311
Cholesteryl Esters in Large LDL	5.2473	0.2229	1.24E+02	0.3037	0.9270	0.0072	-0.0302	0.0445	0.7061	0.8606
Free Cholesterol in Large LDL	115.4163	0.0164	8.14E+05	0.2936	0.9270	0.0061	-0.0073	0.0194	0.3715	0.7818
Triglycerides in Large LDL	296.9233	0.0000	3.76E+10	0.5498	0.9270	0.0015	-0.0043	0.0073	0.6054	0.8311
Concentration of Medium LDL Particles	NA	NA	NA	NA	NA	5.60E-06	-8.82E-06	2.00E-05	0.4463	0.7859

Total Lipids in Medium LDL	5.7698	0.1290	2.58E+02	0.3661	0.9270	0.0066	-0.0248	0.0380	0.6784	0.8532
Phospholipids in Medium LDL	2.73E+03	0.0008	9.38E+09	0.3027	0.9270	0.0018	-0.0062	0.0097	0.6636	0.8532
Cholesterol in Medium LDL	10.5295	0.0489	2.27E+03	0.3904	0.9270	0.0047	-0.0175	0.0269	0.6752	0.8532
Cholesteryl Esters in Medium LDL	17.8766	0.0141	2.27E+04	0.4290	0.9270	0.0026	-0.0141	0.0193	0.7616	0.8820
Free Cholesterol in Medium LDL	3.48E+04	4.95E-05	2.45E+13	0.3143	0.9270	0.0022	-0.0038	0.0081	0.4780	0.7882
Triglycerides in Medium LDL	8.32E+05	6.60E-17	1.05E+28	0.5996	0.9270	0.0001	-0.0022	0.0025	0.9087	0.9597
Concentration of Small LDL Particles	NA	NA	NA	NA	NA	3.04E-06	-4.08E-06	1.02E-05	0.4024	0.7859
Total Lipids in Small LDL	1.69E+02	0.0157	1.83E+06	0.2787	0.9270	0.0043	-0.0083	0.0168	0.5073	0.7995
Phospholipids in Small LDL	7.45E+07	1.63E-06	3.40E+21	0.2587	0.9270	0.0016	-0.0020	0.0053	0.3764	0.7818
Cholesterol in Small LDL	2.29E+03	0.0019	2.80E+09	0.2795	0.9270	0.0028	-0.0056	0.0112	0.5144	0.8006
Cholesteryl Esters in Small LDL	1.70E+04	0.0001	2.19E+12	0.3064	0.9270	0.0016	-0.0047	0.0079	0.6195	0.8366
Free Cholesterol in Small LDL	1.35E+13	4.81E-10	3.78E+35	0.2516	0.9270	0.0012	-0.0012	0.0036	0.3295	0.7818
Triglycerides in Small LDL	2.87E+08	7.18E-39	1.15E+55	0.7221	0.9270	-0.0002	-0.0014	0.0011	0.7754	0.8939
Concentration of Very Large HDL Particles	NA	NA	NA	NA	NA	9.05E-06	-2.75E-06	2.09E-05	0.1326	0.6370
Total Lipids in Very Large HDL	59.0839	0.0040	8.70E+05	0.4048	0.9270	0.0075	-0.0023	0.0173	0.1328	0.6370
Phospholipids in Very Large HDL	827.3974	1.63E-05	4.19E+10	0.4580	0.9270	0.0040	-0.0015	0.0095	0.1584	0.6370
Cholesterol in Very Large HDL	2.86E+04	1.13E-05	7.23E+13	0.3528	0.9270	0.0036	-0.0007	0.0079	0.1035	0.6370
Cholesteryl Esters in Very Large HDL	9.67E+05	7.70E-08	1.21E+19	0.3705	0.9270	0.0027	-0.0005	0.0058	0.0963	0.6370
Free Cholesterol in Very Large HDL	7.85E+16	9.56E-17	6.45E+49	0.3144	0.9270	0.0009	-0.0003	0.0020	0.1324	0.6370
Triglycerides in Very Large HDL	9.15E+13	1.58E-78	5.29E+105	0.7655	0.9270	-2.68E-06	-0.0006	0.0006	0.9930	0.9930
Concentration of Large HDL Particles	NA	NA	NA	NA	NA	3.77E-05	-1.52E-05	0.0001	0.1620	0.6370

Total Lipids in Large HDL	2.6186	0.0170	4.03E+02	0.7080	0.9270	0.0135	-0.0081	0.0351	0.2214	0.6979
Phospholipids in Large HDL	6.0359	4.70E-05	7.75E+05	0.7645	0.9270	0.0043	-0.0054	0.0140	0.3812	0.7844
Cholesterol in Large HDL	7.1799	0.0011	4.60E+04	0.6593	0.9270	0.0097	-0.0027	0.0221	0.1253	0.6370
Cholesteryl Esters in Large HDL	8.8517	0.0001	8.53E+05	0.7096	0.9270	0.0077	-0.0020	0.0173	0.1192	0.6370
Free Cholesterol in Large HDL	1.04E+05	4.85E-11	2.23E+20	0.5213	0.9270	0.0020	-0.0009	0.0050	0.1771	0.6370
Triglycerides in Large HDL	0.0569	1.64E-26	1.98E+23	0.9208	0.9717	-0.0005	-0.0029	0.0019	0.6740	0.8532
Concentration of Medium HDL Particles	NA	NA	NA	NA	NA	-0.0001	-0.0001	0.0000	0.1147	0.6370
Total Lipids in Medium HDL	0.0940	0.0003	3.32E+01	0.4296	0.9270	-0.0174	-0.0384	0.0037	0.1053	0.6370
Phospholipids in Medium HDL	0.0174	1.98E-07	1.53E+03	0.4854	0.9270	-0.0093	-0.0206	0.0021	0.1085	0.6370
Cholesterol in Medium HDL	0.0009	6.00E-10	1.38E+03	0.3349	0.9270	-0.0062	-0.0134	0.0009	0.0877	0.6370
Cholesteryl Esters in Medium HDL	0.0002	1.60E-11	2.11E+03	0.2998	0.9270	-0.0053	-0.0114	0.0007	0.0840	0.6370
Free Cholesterol in Medium HDL	5.06E-08	1.89E-44	1.35E+29	0.6946	0.9270	-0.0009	-0.0025	0.0006	0.2422	0.7095
Triglycerides in Medium HDL	0.0025	1.26E-18	4.89E+12	0.7385	0.9270	-0.0019	-0.0059	0.0022	0.3723	0.7818
Concentration of Small HDL Particles	7.77E-23	7.27E-230	8.31E+184	0.8342	0.9622	-0.0002	-0.0004	0.0001	0.1868	0.6370
Total Lipids in Small HDL	0.4875	0.0091	2.60E+01	0.7233	0.9270	-0.0233	-0.0524	0.0058	0.1160	0.6370
Phospholipids in Small HDL	0.2198	0.0002	2.34E+02	0.6700	0.9270	-0.0148	-0.0318	0.0022	0.0871	0.6370
Cholesterol in Small HDL	0.2518	7.88E-06	8.05E+03	0.7944	0.9382	-0.0070	-0.0176	0.0035	0.1915	0.6370
Cholesteryl Esters in Small HDL	0.0971	3.07E-07	3.07E+04	0.7181	0.9270	-0.0056	-0.0140	0.0028	0.1919	0.6370
Free Cholesterol in Small HDL	14.8852	1.87E-18	1.18E+20	0.9032	0.9702	-0.0014	-0.0042	0.0013	0.3088	0.7818
Triglycerides in Small HDL	0.1090	1.16E-18	1.02E+16	0.9115	0.9702	-0.0015	-0.0050	0.0021	0.4201	0.7859

Phospholipids to Total Lipids in Chylomicrons and Extremely Large VLDL percentage	0.8680	0.7457	1.0104	0.0677	0.9270	-0.0850	-0.7863	0.6162	0.8121	0.9182
Cholesterol to Total Lipids in Chylomicrons and Extremely Large VLDL percentage	1.0365	0.9987	1.0758	0.0587	0.9270	1.4288	-0.8375	3.6950	0.2164	0.6909
Cholesteryl Esters to Total Lipids in Chylomicrons and Extremely Large VLDL percentage	1.0535	1.0076	1.1014	0.0219	0.9270	0.6834	-1.0025	2.3693	0.4267	0.7859
Free Cholesterol to Total Lipids in Chylomicrons and Extremely Large VLDL percentage	1.0280	0.8883	1.1895	0.7112	0.9270	0.7453	-0.0370	1.5277	0.0619	0.6065
Triglycerides to Total Lipids in Chylomicrons and Extremely Large VLDL percentage	0.9752	0.9376	1.0144	0.2119	0.9270	-1.3437	-3.9197	1.2323	0.3064	0.7818
Phospholipids to Total Lipids in Very Large VLDL percentage	1.2556	0.9084	1.7354	0.1682	0.9270	0.1941	-0.2737	0.6619	0.4159	0.7859
Cholesterol to Total Lipids in Very Large VLDL percentage	1.0716	0.9896	1.1605	0.0886	0.9270	1.2754	0.0735	2.4774	0.0376	0.5066
Cholesteryl Esters to Total Lipids in Very Large VLDL percentage	1.0890	0.9842	1.2050	0.0985	0.9270	0.9459	-0.0098	1.9016	0.0524	0.5672
Free Cholesterol to Total Lipids in Very Large VLDL percentage	1.3064	0.9469	1.8025	0.1036	0.9270	0.3295	0.0236	0.6354	0.0347	0.5066
Triglycerides to Total Lipids in Very Large VLDL percentage	0.9394	0.8785	1.0046	0.0677	0.9270	-1.4696	-2.8624	-0.0768	0.0387	0.5066

Phospholipids to Total Lipids in Large VLDL percentage	1.0522	0.8517	1.3000	0.6369	0.9270	-0.0335	-0.5354	0.4684	0.8958	0.9574
Cholesterol to Total Lipids in Large VLDL percentage	1.0837	0.9733	1.2066	0.1426	0.9270	0.8795	0.0239	1.7351	0.0439	0.5392
Cholesteryl Esters to Total Lipids in Large VLDL percentage	1.1133	0.9597	1.2914	0.1565	0.9270	0.6956	0.0528	1.3385	0.0339	0.5066
Free Cholesterol to Total Lipids in Large VLDL percentage	1.2809	0.8974	1.8283	0.1727	0.9270	0.1838	-0.0733	0.4409	0.1611	0.6370
Triglycerides to Total Lipids in Large VLDL percentage	0.9510	0.8788	1.0292	0.2127	0.9270	-0.8460	-1.9895	0.2975	0.1469	0.6370
Phospholipids to Total Lipids in Medium VLDL percentage	1.2162	0.8700	1.7001	0.2522	0.9270	0.5058	0.1559	0.8556	0.0046	0.3294
Cholesterol to Total Lipids in Medium VLDL percentage	1.0762	0.9566	1.2108	0.2219	0.9270	1.6176	0.4107	2.8246	0.0086	0.3589
Cholesteryl Esters to Total Lipids in Medium VLDL percentage	1.1034	0.9393	1.2962	0.2312	0.9270	1.1614	0.2685	2.0543	0.0108	0.3851
Free Cholesterol to Total Lipids in Medium VLDL percentage	1.2962	0.8572	1.9600	0.2188	0.9270	0.4563	0.1308	0.7817	0.0060	0.3294
Triglycerides to Total Lipids in Medium VLDL percentage	0.9452	0.8639	1.0341	0.2193	0.9270	-2.1234	-3.6551	-0.5917	0.0066	0.3294
Phospholipids to Total Lipids in Small VLDL percentage	1.2644	0.8758	1.8255	0.2106	0.9270	0.4089	0.0512	0.7665	0.0251	0.4784
Cholesterol to Total Lipids in Small VLDL percentage	1.0968	0.9400	1.2798	0.2406	0.9270	1.0407	0.1532	1.9283	0.0216	0.4784

Cholesteryl Esters to Total Lipids in Small VLDL percentage	1.1594	0.9023	1.4899	0.2476	0.9270	0.6093	0.0698	1.1488	0.0269	0.4784
Free Cholesterol to Total Lipids in Small VLDL percentage	1.2163	0.8544	1.7314	0.2772	0.9270	0.4315	0.0511	0.8120	0.0262	0.4784
Triglycerides to Total Lipids in Small VLDL percentage	0.9341	0.8366	1.0430	0.2258	0.9270	-1.4496	-2.6798	-0.2195	0.0209	0.4784
Phospholipids to Total Lipids in Very Small VLDL percentage	1.4871	0.6764	3.2693	0.3235	0.9270	-0.0712	-0.2134	0.0711	0.3268	0.7818
Cholesterol to Total Lipids in Very Small VLDL percentage	1.0307	0.8732	1.2166	0.7207	0.9270	0.7955	-0.0442	1.6352	0.0633	0.6065
Cholesteryl Esters to Total Lipids in Very Small VLDL percentage	1.0144	0.8455	1.2171	0.8778	0.9661	0.6966	-0.0346	1.4278	0.0618	0.6065
Free Cholesterol to Total Lipids in Very Small VLDL percentage	3.4916	0.7685	15.8635	0.1054	0.9270	0.0989	-0.0326	0.2304	0.1404	0.6370
Triglycerides to Total Lipids in Very Small VLDL percentage	0.9438	0.7813	1.1402	0.5486	0.9270	-0.7244	-1.5046	0.0559	0.0688	0.6118
Phospholipids to Total Lipids in IDL percentage	0.8691	0.4310	1.7524	0.6950	0.9270	-0.0766	-0.2412	0.0880	0.3615	0.7818
Cholesterol to Total Lipids in IDL percentage	1.0733	0.8407	1.3704	0.5702	0.9270	0.2748	-0.3134	0.8631	0.3596	0.7818
Cholesteryl Esters to Total Lipids in IDL percentage	1.0634	0.8006	1.4124	0.6713	0.9270	0.1222	-0.3386	0.5830	0.6032	0.8311
Free Cholesterol to Total Lipids in IDL percentage	1.2332	0.6271	2.4250	0.5435	0.9270	0.1527	-0.0840	0.3893	0.2060	0.6748
Triglycerides to Total Lipids in IDL percentage	0.9154	0.6693	1.2522	0.5804	0.9270	-0.1983	-0.7166	0.3201	0.4533	0.7859

Phospholipids to Total Lipids in Large LDL percentage	1.0544	0.4462	2.4913	0.9040	0.9702	0.1239	-0.0264	0.2741	0.1060	0.6370
Cholesterol to Total Lipids in Large LDL percentage	1.1261	0.6784	1.8692	0.6461	0.9270	-0.1126	-0.4766	0.2514	0.5442	0.8145
Cholesteryl Esters to Total Lipids in Large LDL percentage	1.0370	0.6582	1.6335	0.8756	0.9661	-0.2925	-0.5652	-0.0199	0.0355	0.5066
Free Cholesterol to Total Lipids in Large LDL percentage	1.1483	0.6378	2.0674	0.6449	0.9270	0.1800	-0.0784	0.4384	0.1721	0.6370
Triglycerides to Total Lipids in Large LDL percentage	0.9036	0.5878	1.3891	0.6441	0.9270	-0.0113	-0.4089	0.3864	0.9558	0.9714
Phospholipids to Total Lipids in Medium LDL percentage	1.3900	0.5801	3.3305	0.4602	0.9270	0.0288	-0.1188	0.1763	0.7020	0.8606
Cholesterol to Total Lipids in Medium LDL percentage	1.0288	0.7015	1.5090	0.8843	0.9661	0.0726	-0.2655	0.4106	0.6738	0.8532
Cholesteryl Esters to Total Lipids in Medium LDL percentage	0.9599	0.6863	1.3425	0.8111	0.9470	-0.1410	-0.4930	0.2111	0.4324	0.7859
Free Cholesterol to Total Lipids in Medium LDL percentage	1.0977	0.7251	1.6619	0.6594	0.9270	0.2134	-0.1625	0.5893	0.2656	0.7515
Triglycerides to Total Lipids in Medium LDL percentage	0.8874	0.5565	1.4152	0.6159	0.9270	-0.1013	-0.4375	0.2349	0.5545	0.8145
Phospholipids to Total Lipids in Small LDL percentage	0.9885	0.6942	1.4076	0.9489	0.9836	0.1441	-0.1894	0.4775	0.3969	0.7844
Cholesterol to Total Lipids in Small LDL percentage	1.0992	0.7809	1.5471	0.5878	0.9270	0.0830	-0.3079	0.4740	0.6771	0.8532

Cholesteryl Esters to Total Lipids in Small LDL percentage	1.0127	0.7340	1.3973	0.9385	0.9836	-0.1513	-0.5450	0.2425	0.4512	0.7859
Free Cholesterol to Total Lipids in Small LDL percentage	1.1239	0.7449	1.6956	0.5778	0.9270	0.2343	-0.1867	0.6553	0.2751	0.7529
Triglycerides to Total Lipids in Small LDL percentage	0.8641	0.5471	1.3647	0.5310	0.9270	-0.2270	-0.6044	0.1503	0.2382	0.7060
Phospholipids to Total Lipids in Very Large HDL percentage	0.9703	0.9153	1.0286	0.3118	0.9270	0.9033	-0.4050	2.2116	0.1758	0.6370
Cholesterol to Total Lipids in Very Large HDL percentage	1.0028	0.8736	1.1511	0.9685	0.9836	-0.4034	-1.4946	0.6879	0.4686	0.7859
Cholesteryl Esters to Total Lipids in Very Large HDL percentage	0.9191	0.7862	1.0745	0.2899	0.9270	-0.0568	-0.8283	0.7147	0.8851	0.9574
Free Cholesterol to Total Lipids in Very Large HDL percentage	1.1451	0.9507	1.3793	0.1535	0.9270	-0.3465	-0.9132	0.2202	0.2306	0.7030
Triglycerides to Total Lipids in Very Large HDL percentage	1.0430	0.9862	1.1031	0.1406	0.9270	-0.5000	-1.6022	0.6023	0.3738	0.7818
Phospholipids to Total Lipids in Large HDL percentage	1.0520	0.8572	1.2912	0.6274	0.9270	-0.9285	-1.5542	-0.3029	0.0037	0.3294
Cholesterol to Total Lipids in Large HDL percentage	0.9884	0.8694	1.1238	0.8590	0.9661	0.4078	-0.6182	1.4338	0.4357	0.7859
Cholesteryl Esters to Total Lipids in Large HDL percentage	0.9779	0.8441	1.1329	0.7661	0.9270	0.3207	-0.5807	1.2222	0.4854	0.7900

Free Cholesterol to Total Lipids in Large HDL percentage	1.1255	0.5748	2.2037	0.7303	0.9270	0.0871	-0.1474	0.3215	0.4665	0.7859
Triglycerides to Total Lipids in Large HDL percentage	0.9806	0.7843	1.2259	0.8632	0.9661	0.5207	-0.1487	1.1901	0.1273	0.6370
Phospholipids to Total Lipids in Medium HDL percentage	1.0090	0.7498	1.3577	0.9529	0.9836	-0.4988	-0.7874	-0.2101	0.0007	0.1786
Cholesterol to Total Lipids in Medium HDL percentage	1.0858	0.7908	1.4910	0.6108	0.9270	-0.1207	-0.6117	0.3702	0.6296	0.8366
Cholesteryl Esters to Total Lipids in Medium HDL percentage	1.0472	0.7810	1.4043	0.7578	0.9270	-0.1424	-0.6294	0.3446	0.5665	0.8181
Free Cholesterol to Total Lipids in Medium HDL percentage	4.3551	0.7019	27.0207	0.1141	0.9270	0.0216	-0.0443	0.0875	0.5209	0.8057
Triglycerides to Total Lipids in Medium HDL percentage	0.8775	0.5492	1.4022	0.5849	0.9270	0.6195	0.1186	1.1205	0.0154	0.4563
Phospholipids to Total Lipids in Small HDL percentage	0.8777	0.4772	1.6142	0.6748	0.9270	-0.1499	-0.3692	0.0694	0.1801	0.6370
Cholesterol to Total Lipids in Small HDL percentage	1.0959	0.7461	1.6097	0.6407	0.9270	0.0200	-0.3700	0.4100	0.9199	0.9597
Cholesteryl Esters to Total Lipids in Small HDL percentage	1.0106	0.7515	1.3589	0.9445	0.9836	-0.0737	-0.4869	0.3396	0.7267	0.8742
Free Cholesterol to Total Lipids in Small HDL percentage	1.5365	0.7607	3.1035	0.2311	0.9270	0.0937	-0.0066	0.1940	0.0671	0.6118
Triglycerides to Total Lipids in Small HDL percentage	0.9168	0.5095	1.6498	0.7720	0.9270	0.1299	-0.1222	0.3821	0.3123	0.7818

[a]Models were adjusted for age, sex, ethnicity, Townsend deprivation index, smoking status, alcohol consumption, physical activity, education, waist circumference, hypertension status, diabetes status, ALT, HDL, and myopia status.

[b]Models were adjusted for age, sex, ethnicity, Townsend deprivation index, smoking status, alcohol consumption, physical activity, education, waist circumference, hypertension status, diabetes status, ALT and HDL.

HR Hazards ratio, CI Confidence interval, ALT Alanine aminotransferase, LDL Low-density lipoprotein, VLDL Very low-density lipoprotein, IDL Intermediate-density lipoprotein, HDL High-density lipoprotein, HDL-C High-density lipoprotein cholesterol.

NA denotes statistical models with convergence failure or sparse data.

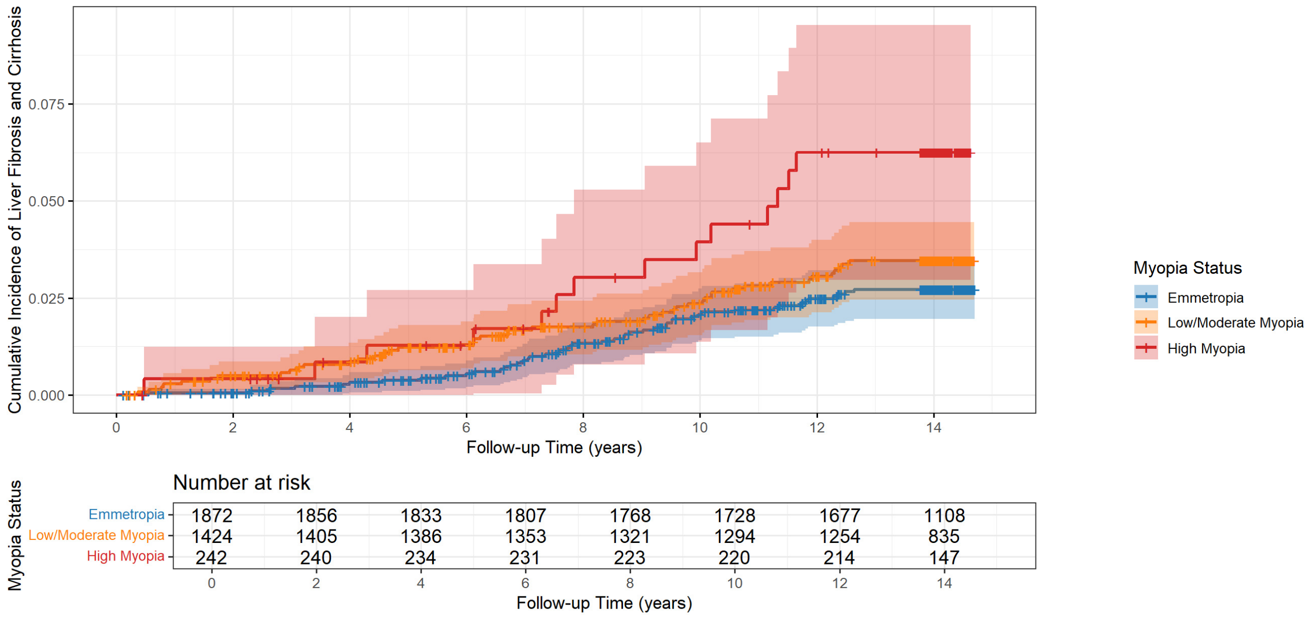

Figure S1. Kaplan–Meier curves for cumulative incidence of liver fibrosis and cirrhosis according to myopia status
